# Adult ADHD and Comorbid Somatic Disease: A Systematic Literature Review

**DOI:** 10.1177/1087054716669589

**Published:** 2016-09-01

**Authors:** Johanne Telnes Instanes, Kari Klungsøyr, Anne Halmøy, Ole Bernt Fasmer, Jan Haavik

**Affiliations:** 1 Department of Biomedicine, University of Bergen, Norway; 2 Department of Global Public Health and Primary Care, University of Bergen, Norway; 3 K.G. Jebsen Centre for Neuropsychiatric Disorders, University of Bergen, Norway; 4 Norwegian Institute of Public Health, Bergen, Norway; 5 Haukeland University Hospital, Bergen, Norway; 6 Department of Clinical Medicine, University of Bergen, Norway

**Keywords:** adult ADHD, asthma, migraine, sleep disorders, review

## Abstract

**Objective:** To systematically review, synthesize, and appraise available evidence, connecting adult ADHD with somatic disease. **Method:** Embase, Psychinfo, and Medline databases were searched for studies published from 1994 to 2015 addressing adult ADHD and somatic comorbidity. Somatic conditions were classified according to International Classification of Diseases (ICD-10) codes. Levels of evidence were graded as inconclusive, tentative, or well documented. **Results:** Most of the 126 studies included in the qualitative synthesis were small and of modest quality. Obesity, sleep disorders, and asthma were well-documented comorbidities in adult ADHD. Tentative evidence was found for an association between adult ADHD and migraine and celiac disease. In a large health registry study, cardiovascular disease was not associated with adult ADHD. **Conclusion:** There are few large systematic studies using standardized diagnostic criteria evaluating adult ADHD and somatic comorbidities. Significant associations are found between adult ADHD and several somatic diseases, and these are important to consider when assessing and treating either adult ADHD or the somatic diseases.

ADHD is a common neuropsychiatric disorder defined by a persistent pattern of inattention and/or hyperactivity/impulsivity that interferes with functioning or development (American Psychiatric Association [APA], 2013). The first systematic studies of ADHD focused on school-aged boys (Still, 1902). Later, it was recognized that many girls have similar problems, and that symptoms persist into adulthood in the majority of cases, with worldwide prevalence estimates of ADHD around 2.5% to 3% in the adult population (Polanczyk, de Lima, Horta, Biederman, & Rohde, 2007; Simon, Czobor, Balint, Meszaros, & Bitter, 2009).

In addition to the core clinical symptoms of ADHD, psychiatric and non-psychiatric coexisting problems and clinical conditions have been described in ADHD patients (Angold, Costello, & Erkanli, 1999). In particular, psychiatric comorbid conditions are recognized in both children and adults, and pose considerable clinical and public health challenges (Angold et al., 1999; Halmoy et al., 2010).

Recognition of medical/somatic conditions is also a key component in the routine clinical assessment of psychiatric patients. Failure to diagnose medical conditions can lead to misdiagnosis or incorrect treatment, with potentially serious consequences. According to the current diagnostic criteria for ADHD in the *Diagnostic and Statistical Manual of Mental Disorders* (5th ed.; *DSM-5*; APA, 2013), the diagnosis of ADHD is only considered appropriate if the disturbance is not judged to be the direct pathophysiological consequence of a specific medical condition (e.g., multiple sclerosis, stroke, hypothyroidism). However, in the most recent version of the International Classification of Diseases and Related Health Problems (ICD-10; World Health Organization, 1992), it is also emphasized that psychiatric syndromes may be causally related to cerebral and systemic diseases, and that proper diagnosis will require two codes: one for the psychopathological syndrome and the other for the underlying disorder.

Compared with the extensive descriptions of psychiatric comorbidity, somatic comorbidity in ADHD has received less attention in the research literature, particularly among adults. This discrepancy is obvious in the recent diagnostic definition of ADHD (APA, 2013), where many psychiatric disorders are listed either as possible differential diagnoses or as comorbid conditions. The only non-psychiatric disorder specifically mentioned is medication-induced symptoms of ADHD. Associated medical conditions have been studied more in other psychiatric disorders, where they are also considered to contribute to a lower quality of life and reduced life expectancy. In schizophrenia, it is known that weight gain, diabetes, metabolic syndrome, and cardiovascular disease are common, and it is speculated that a shared vulnerability for psychosis and medical conditions can explain some of this comorbidity (Ringen, Engh, Birkenaes, Dieset, & Andreassen, 2014). Population-based prospective studies have documented an increased risk of premature death and reduced life expectancy also for ADHD patients (Dalsgaard, Ostergaard, Leckman, Mortensen, & Pedersen, 2015), but it is unclear if this risk is mediated by coexisting medical diseases.

The primary objective of this review was to obtain an overview of, and evaluate, the literature covering this topic during the past 20 years. Secondary objectives were to inform clinicians on the most common somatic comorbid conditions to enhance optimal patient evaluation and treatment, and to identify particular areas of research that should be further investigated.

## Method

### Literature Search Strategies and Data Sources

We performed a systematic review of the literature addressing adult ADHD and somatic comorbidity. The search strategy was developed in collaboration with a university librarian experienced in systematic medical literature searches. The electronic databases Embase, Psychinfo, and Medline were searched in December 2014 and January 2015, limiting the search to study participants above 18 years of age. The search was finalized on January 26, 2015, retrieving 4,091 papers. The detailed electronic search strategy is provided in Supplementary 1.

After removing duplicates and studies published prior to 1994 when the *Diagnostic and Statistical Manual of Mental Disorders* (4th ed.; *DSM-IV*; APA, 1994) was introduced, J.T.I. screened title and abstract in the remaining studies, excluding papers that clearly did not fulfill the inclusion criteria listed below. Furthermore, reference lists of the retrieved papers were hand searched to identify additional relevant articles. Other papers of interest found in manual search published January 2015 to February 2016 were also included. In total, 208 papers were assessed in full text by at least one of the authors, depending on their experience and fields of expertise, and all papers were discussed by at least two. Extraction of data was checked and harmonized by two authors (J.T.I. and K.K). Of the 208 papers, 82 were excluded using the criteria listed below. Of the 126 remaining papers, 98 contained original data, 26 were classified as reviews, one a letter to the editor and one an annotation.

**Figure 1.**
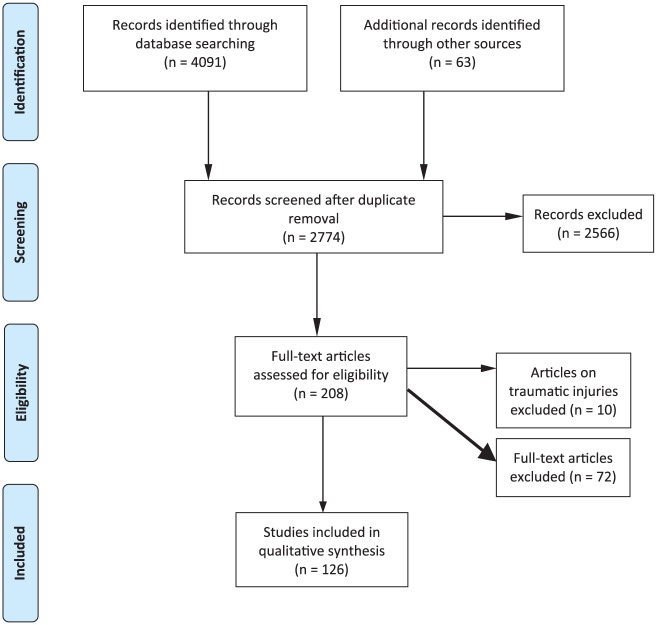
Flow diagram (from PRISME). *Source.* Moher, Liberati, Tetzlaff, and Altman (2009).

The specific number of included and excluded papers at each step is provided in a PRISMA flow chart (Figure 1).

All the 126 studies, both the individual studies and reviews, are referred to in the text. Sources of bias in the 98 individual studies were considered to be mainly related to study design, the selection of participants, the size of the study, and the methods used to define ADHD and the comorbid disorders. All these characteristics were therefore assessed and are tabulated in a supplementary table (Supplementary 2). Reviews are omitted from this table (Supplementary 4 for list). Some additional studies not fulfilling the inclusion criteria have been mentioned as part of the discussion.

### Study Selection Criteria

#### Inclusion criteria

Studies focusing on the comorbidity between somatic disease and ADHD in adults (i.e. 18 years or older) were included. ADHD was defined according to ICD or DSM criteria (ICD-9/ICD-10/DSM-III/DSM-IV/DSM-5). As indicated in a supplementary table (Supplementary 2), different protocols have been used to classify individuals as having adult ADHD, that is, either (a) adult ADHD clinically diagnosed, included the use of semi-structured interviews; (b) ADHD medication used as a proxy for ADHD diagnosis; (c) symptoms of ADHD measured by a validated ADHD Symptom Rating scale; (d) information on ADHD cases from clinical databases. The properties and utility of some adult ADHD instruments have been reviewed (Haavik, Halmoy, Lundervold, & Fasmer, 2010) and are summarized in Supplementary 3.

For all studies, the reported diagnoses of somatic disease/comorbidity were fit into broad disease categories as defined in the ICD-10. In the cited studies, these diagnoses were obtained either after clinical evaluation or from self-reports. Due to the large number of somatic comorbidities studied and the different protocols involved, it was not feasible to systematically describe the inclusion criteria for each of the individual somatic comorbidities, but an overview of how the somatic diseases are defined, is summarized in Supplementary 2.

Instruments used for ADHD assessment and the most commonly used methods to assess for comorbid disorders described in the included studies are briefly described and listed in Supplementary 3.

#### Exclusion criteria

The following exclusion criteria were applied: (a) studies not including ADHD as described under inclusion criteria, (b) studies including *only* children and adolescents, (c) publications not subject to peer review, (d) non-English papers, (e) single case studies, (f) studies describing only psychiatric comorbidities as classified in *ICD-10* Chapter V: Mental and behavioral disorders, and (g) pharmacological trials, for example, focusing on specific treatment options and not on the comorbid disorder itself. We also excluded studies on traumatic incidents, as we consider these to be outside our main focus which is somatic diseases comorbid with adult ADHD.

### Classification of Studies

Based on available evidence, the 98 individual studies were classified into three categories (Table 1). Category 1 includes conditions where the association between ADHD and somatic disease is well established and described in meta-analysis or systematic reviews. Category 2 includes conditions where there is tentative evidence for an association, the associations being described in cohort or case-control studies with clinically diagnosed ADHD and the somatic diseases not only being based on self-report questionnaires. Comorbidities shown in large population-based studies with diagnoses retrieved from clinical databases were also included in this category. Category 3 includes conditions where the evidence was considered too weak to make conclusions, including associations described only in studies where ADHD and/or somatic comorbidities are not clinically diagnosed (i.e., based on self-report questionnaires only) or where the evidence is limited. This category also includes conditions where the combined results clearly showed conflicting results. Studies on conditions lacking information on diagnostic protocols or the age distribution of the ADHD participants were also categorized in Category 3.

**Table 1.** Name of Disease Category, ICD-10 Code, and Number of Individual Studies Investigating the Association Between Adult ADHD and Somatic Disease.

Diagnosis	ICD-10 code	Number of individual studies	Association and quality of evidence^a^
In general		4	
Resistance to thyroid hormone	E07.8	1	Association (3)
Hypothyroidism	E00-E03	1	Association (3)
Diabetes	E10-E14	3	No/negative association (3)
Nutritional diseases			
Obesity	E66	22	**Association (1)**
Metabolic disorders	E70-E90		
In general		1	Association (3)
Albinism	E70.3	1	Association (3)
Maple syrup urine disease	E71.0	1	Association (3)
Diseases of the nervous system			
Restless legs	G25	6	Association (3)
Dementia with Lewy bodies	G31.83	1	Association (3)
Epilepsy	G40	3	Association (3)
Migraine	G43	2	**Association (2)**
Sleep disorders	G47	25	**Association (1)**
Myotonic dystrophy	G71.1	2	Association (3)
Chronic fatigue syndrome	G93.3	2	Association (3)
Diseases of the circulatory system	Chapter IX	4	**No association (2)**
Allergic diseases			
In general		2	Association (3)
Allergic rhinitis	J30	1	Association (3)
Respiratory disorders	Chapter X		
In general		2	Association (3)
Asthma	J46	7	**Association (1)**
Diseases of the digestive system	Chapter K		
In general		1	Association (3)
Irritable bowel syndrome	K58	2	Association?^b^ (3)
Celiac disease	K90.9	3	**Association (2)**
Skin disorders	Chapter XII		
In general		1	No association (3)
Atopic dermatitis	L20	1	Association (3)
Alopecia areata	L63	1	No association (3)
Acne (ICD-10: L70)	L70	1	Association (3)
Musculoskeletal disorders	Chapter XIII		
In general		3	Association (3)
Rheumatoid arthritis	M05-M06	1	No association (3)
Systemic lupus erythematosus	M32	2	Association (3)
Fibromyalgia	M79.7	2	Association (3)
Calvé-Legg-Perthes	M91.1	1	Association (3)
Congenital syndromes and anomalies	Chapter XVII	12	
Symptoms/signs involving the urinary system	**R30-R39**		
In general		1	No association (3)
Enuresis	R32	3	Association?^b^ (3)

*Note.* ICD-10 = International Classification of Diseases; Conditions classified in (1) or ( 2) in bold.

a The reported studies were classified into conditions (1) where the association between ADHD and the somatic disease is well established, (2) where there is tentative evidence for an association, and (3) where evidence is still too weak to make conclusions.

b Conflicting evidence. One study shows no association, another study/studies show association. See text for more information.

The somatic diseases included in the present review have been broadly grouped using ICD-10 codes, although this classification in some instances may be arbitrary (e.g. classifications of sleep problems), due to the application of various diagnostic criteria.

### Measurements

#### ADHD scales

The Adult ADHD Self-Report Scale (ASRS) was developed in conjunction with the World Health Organization and is designed to measure current ADHD symptoms. A high symptom score on ASRS is not sufficient to clinically diagnose ADHD in adults but is frequently used in research literature to define study populations with possible ADHD (Kessler et al., 2005). The Wender Utah Rating Scale (WURS) retrospectively assesses symptoms of ADHD in childhood (Ward, Wender, & Reimherr, 1993). For additional measurements, see Supplement 3.

#### Measure of obesity

Body mass index (BMI) is defined as weight in kilograms divided by height in meters squared. In adults, <18.5 kg/m^2^ is defined as underweight, 18.5 to <25 kg/m^2^ is defined as normal, 25.0 to <30 kg/m^2^ is defined as overweight, and a BMI of ≥30 kg/m^2^ is defined as obese (WHO, 1995). BMI is as simple and easy way to evaluate obesity and is useful to evaluate obesity trends in the general population. However, BMI does not provide an accurate measurement of body fat, nor does it take sex, age, and ethnicity into account (Bhurosy & Jeewon, 2013). For additional measurements, see Supplement 3.

#### Sleep measurements

Polysomnography is used to record several physiologic parameters relevant to sleep, such as electroencephalography (EEG), electrooculography (EOG), electrocardiography (ECG), chin- and anterio tibialis electromyography (EMG), respiratory effort, airflow, and oximetry (Chesson et al., 1997). Polysomnography is used in assessing a number of different sleep-related disorders, such as restless legs syndrome periodic limb movements during sleep, central hypersomnias, circadian rhythm sleep disorder, and sleep-disordered breathing (Kushida et al., 2005). For additional measurements, see Supplement 3.

## Results

### Literature Search and Selection of Papers

A range of different medical conditions have been studied in connection with ADHD in adults, as shown in Table 1. Most studies represent small clinical samples of ADHD patients assessed for a limited number of comorbid conditions, or clinical studies of somatic diseases where comorbid adult ADHD or ADHD symptoms also were diagnosed. A limited number of population-based cohort studies have also been published during this 20-year period.

The diagnostic protocols and quality of the clinical assessments of ADHD varied between studies, as was the case for the somatic/medical comorbid conditions. However, for the purpose of this literature study, we did not consider it appropriate to limit this overview to a specific diagnostic protocol or classification system. Thus, the cited prevalences are not directly comparable.

ADHD comorbidity with sleep disorders or obesity has previously been reviewed. However, for the majority of conditions mentioned, the differences in research designs, the limited number of cases, and the fact that most conditions were only described in few studies made it unfeasible to perform meaningful meta-analyses of prevalences.

### Somatic Health in General

ADHD is associated with generally impaired somatic health (Nigg, 2013) and increased medical costs (Secnik, Swensen, & Lage, 2005) when compared with unaffected sex- and age-matched controls, even when no differences in health habits were identified (Spencer, Faraone, Tarko, McDermott, & Biederman, 2014). In a prospective U.S. study including 72 ADHD cases and 479 controls, ADHD was diagnosed through clinical interviews in adolescence. When reassessed at >10 years, having ADHD was significantly associated with impaired general physical health (Brook, Brook, Zhang, Seltzer, & Finch, 2013). When retrospectively investigating U.S. health care claims for 2006, matching adults with ADHD (*n* = 31,752) to non-ADHD (*n* = 95,256), adults with ADHD had more physical comorbidities and were more likely to use non-psychiatric health care compared with controls (Hodgkins, Montejano, Sasane, & Huse, 2011).

### Obesity (ICD-10: E66)

#### Clinical samples

Obesity is one of the most frequently reported comorbid medical conditions in adult ADHD. The prevalences of clinically diagnosed ADHD and suspected ADHD based on rating scales have been reported to be 10% to 32% in studies exploring adults with obesity or obesity treatment, mainly including female participants (Alfonsson, Parling, & Ghaderi, 2012; Altfas, 2002; Docet, Larranaga, Fernandez Sastre, & García-Mayor, 2010; Fleming, Levy, & Levitan, 2005; Levy, Fleming, & Klar, 2009; Pagoto et al., 2010; Vogel et al., 2015). Similarly, in a Dutch study with 202 clinically assessed adult ADHD patients and 189 controls, 16.8% of the ADHD patients had BMI 30 to 39, compared with only 3.7% of the controls (*p* < .001; Bijlenga, van der Heijden, et al., 2013). In contrast, a small U.S. study (137 ADHD participants, 124 controls) found no significant differences between participants in age-corrected BMI (Biederman, Spencer, Monuteaux, & Faraone, 2010).

It is unclear whether the association between ADHD and obesity is dependent on ADHD subtypes (Davis et al., 2009), but there are indications of higher proportions of inattentive symptoms/subtypes (Altfas, 2002; Fleming et al., 2005).

#### Non-clinical samples

The above studies were conducted in clinical settings. When examining population-based, non-clinical samples, results have been less consistent. In a population-based U.S. study, Pagoto et al. assessed 6,735 participants between 18 and 44 years (52% females; Pagoto et al., 2009). A diagnosis of adult ADHD was associated with increased risk of overweight and obesity, also when adjusting for demographic characteristics and major depressive disorder, but not when controlling for binge eating disorder in the past year. In a French study using ASRS to assess adult ADHD symptoms, the prevalence of being overweight and obese was approximately doubled for persons reporting ADHD symptoms (Caci, Morin, & Tran, 2014). In a German cross-sectional study including 1,622 residents between 18 and 64 years, the prevalence of ADHD based on self-reported symptoms in obese participants was 9.7% (de Zwaan et al., 2011). Similar to findings of Altfas and Pagoto et al. (Pagoto et al., 2009), the prevalence increased with the degree of obesity. The associations between estimated ADHD and obesity were significant when adjusting for sociodemographic characteristics, symptoms of anxiety and depression and also purging behaviors, indicating that the relationship between obesity and ADHD in adulthood is not fully explained by binge eating. A small non-clinical Canadian study also found associations between ADHD symptoms and overweight/obesity, independent of binge eating (Davis, Levitan, Smith, Tweed, & Curtis, 2006).

A population-based U.S. study included 34,653 participants who were asked about ADHD symptoms (Cortese, Faraone, Bernardi, Wang, & Blanco, 2013). In this study, remittent ADHD was not significantly associated with obesity, whereas there was an association in adults with persistent ADHD. However, after adjusting for mood and anxiety disorders, the association was no longer significant. In contrast, a 33-year follow-up study including 111 males, remittent, but not persistent ADHD was associated with obesity, also after adjusting for sociodemographic characteristics and lifetime mental disorders (Cortese, Ramos-Olazagasti, et al., 2013).

The prevalence and comorbidity of ADHD in older adults have generally been little explored. In a Dutch study including 231 random older participants from the population registries (*Mean*_age_ = 71.6 years), 23 of the participants were clinically diagnosed with ADHD (Semeijn et al., 2013). In this age group, there was no association between ADHD and BMI or waist circumference.

#### Meta-analyses

In a meta-analysis by Cortese et al., on the association between adult ADHD and obesity, 11 data sets with a total of 2,046 adult ADHD participants and 63,747 controls were analyzed, including previously unpublished studies (Cortese et al., 2015). Studies of individuals in bariatric clinics were excluded. The pooled prevalence of obesity was 28.2% (95% confidence interval [CI] = [22.8%, 34.4%]) in adults with ADHD relative to 16.4% [13.4%, 19.9%] in those without ADHD. When analyzing all age groups, age did not influence the association between ADHD and obesity, indicating that the relationship may be present from childhood (Cortese et al., 2016). This was supported by two prospective cohort studies from the United States (Anderson, Cohen, Naumova, & Must, 2006; Cortese, Ramos-Olazagasti, et al., 2013). The association between ADHD and obesity found in the meta-analysis by Cortese et al. remained significant when limiting to studies where ADHD was diagnosed by direct interview, using directly measured height and weight and after adjusting for confounding factors.

In a meta-analysis conducted by Nigg et al., a total of 43 population-based samples or case-control studies including 703,937 participants in all age groups were included (Nigg et al., 2016). The pooled effect size expressed as odds ratio (OR) was 1.22 [1.11, 1.34], increasing to 1.37 [1.19, 1.58] when limiting data to adults of 18 years or more, and was not significant for children.

#### Combined ADHD and obesity comorbid with other conditions

The combination of obesity and ADHD also shows comorbidity with other psychiatric disorders, for instance mood and anxiety disorders (Cortese, Faraone, et al., 2013), and disturbed eating behavior/binge eating (Alfonsson et al., 2012; Davis et al., 2006; Nazar et al., 2014; Strimas et al., 2008). Compared with obese adults without ADHD, obese people with ADHD symptoms are three times more likely to suffer from abnormal eating behaviors (Docet, Larranaga, Perez Mendez, & Garcia-Mayor, 2012). Obesity is also associated with excessive sleepiness, which may produce ADHD symptoms (Cortese, Konofal, & Lecendreux, 2008). A mediation analysis conducted as part of a clinical study, including 114 patients with obesity, 202 adult ADHD patients, and 154 controls, showed that both sleep duration and unstable eating patterns mediated the association between BMI and ADHD symptoms. A link between ADHD, obesity, and iron deficiency has also been discussed (Cortese & Angriman, 2014).

#### Implications for treatment

Several of the cited studies have emphasized the importance of recognizing comorbid conditions for planning optimal treatment of either ADHD or obesity. Treatment for obesity in people with ADHD may be less successful compared with obese people without ADHD (Altfas, 2002; Pagoto et al., 2010), and treatment of comorbid ADHD in obese individuals may improve the treatment for obesity (Cortese & Castellanos, 2014). Clinicians should also consider abnormal eating behaviors as contributing to obesity in ADHD patients (Cortese & Morcillo-Peñalver, 2010; Nazar et al., 2014).

Treating ADHD successfully might help people with obesity and ADHD to better manage overeating (Davis, 2009), reduce self-blame, and facilitate the process of regaining control for persons with abnormal eating behaviors (Cortese, Bernardina, & Mouren, 2007). Behavioral treatment may contribute to weight reduction, but this has not yet been investigated in well-controlled studies (Cortese & Morcillo-Peñalver, 2010). ADHD medication may act on brain pathways involving both ADHD and mediating abnormal eating behaviors (Cortese, Angriman, et al., 2008). It has been hypothesized that stimulant treatment may decrease impulsiveness and thus improve abnormal eating behaviors (Cortese & Morcillo-Peñalver, 2010). Treatment of comorbid ADHD in obese individuals may improve the otherwise poor effects of standard treatment strategies for obesity (Cortese & Angriman, 2014). This is supported by a small Canadian study where ADHD patients treated with stimulant medication had a significant weight reduction, whereas the weight increased in the non-medicated group (Levy et al., 2009). Furthermore, a meta-analysis by Cortese et al. limited to studies on unmedicated patients only showed a pooled estimate for obesity of OR = 1.43 [1.23, 1.67], compared with OR = 1.00 [0.87, 1.15] when limiting to medicated patients only (Cortese et al., 2016).

Finally, clinicians should be aware that comorbid anxiety and mood disorders may be more directly linked to obesity than to ADHD itself, and also take these disorders into account when planning treatment (Cortese, Faraone, et al., 2013).

### Restless Legs Syndrome (RLS; ICD-10: G25)

RLS is a neurological disorder which makes it difficult to fall asleep. RLS has a reported population prevalence of 3% to 34%, generally increasing by age and highest in women (Allen et al., 2005; Milligan & Chesson, 2002; Rijsman, Neven, Graffelman, Kemp, & de Weerd, 2004). It is characterized by an unpleasant feeling in the feet or other limbs, combined with an urge to move the limb to relieve the discomfort. The symptoms primarily occur when a person is relaxed or trying to sleep, and is often combined with paresthesias or dysesthesias. Poor quality of sleep associated with RLS can lead to hyperactivity and lack of concentration, and dopaminergic agents are used to treat the condition.

Two small studies showed that the prevalence of RLS is higher in persons with ADHD compared with controls (Schredl, Alm, & Sobanski, 2007; Zak, Fisher, Couvadelli, Moss, & Walters, 2009), and another small study showed that ADHD is also more common among patients with RLS compared with controls (Wagner, Walters, & Fisher, 2004). People with combined ADHD and RLS had more severe ADHD symptoms compared with those with ADHD without restless legs symptoms (Zak et al., 2009). In a German population- based sample (Roy et al., 2015), crude analysis showed that adult ADHD was associated with RLS. However, this association was no longer significant when adjusting for sleep disturbances. Pearson et al. reported a non-significant increase in the use ADHD medication (amphetamines) in 110 restless legs patients (*M*_age_ 61 years; *p* = .09) compared with 54 age- and race-matched controls (Pearson et al., 2008). Steinlechner (Steinlechner et al., 2011) found that parents of children with ADHD had an increased risk of RLS compared with the population prevalence. There is also evidence of increased psychiatric comorbidity and RLS in families with ADHD (Steinlechner et al., 2011), and that symptoms of restless legs are related to depressive symptoms among ADHD patients (Schredl et al., 2007). Appropriate management of RLS can in some cases cause improvement of the comorbid disorder (Becker & Novak, 2014).

### Epilepsy (ICD-10: G 40)

Epilepsy is a common neurological brain disorder defined as “an enduring predisposition to generate epileptic seizures” and “the neurobiologic, cognitive, psychological, and social consequences of this condition” (Fisher et al., p. 471, 2005).

The cognitive dysfunction and behavioral disturbances associated with epilepsy have similarities with both the core symptoms and adjunctive features of ADHD. The cognitive deficits may be a consequence of recurrent seizure activity in the brain, adverse effects of anti-epileptic drugs, or it could represent an inherent part of the syndrome.

The prevalence of epilepsy in the general population is estimated to be around 0.4% to 1% (Forsgren, Beghi, Oun, & Sillanpaa, 2005; Russ, Larson, & Halfon, 2012), with decreasing prevalence and incidence with age. Thus, like for ADHD, the majority of cases with childhood-onset epilepsy will remit over time, although accompanying symptoms, comorbidity, and impairment may remain (Sillanpaa et al., 2015).

A reciprocal comorbidity between ADHD and epilepsy is well known in pediatric populations (Davis et al., 2010; Socanski, Aurlien, Herigstad, Thomsen, & Larsen, 2013); however, less is known about the comorbidity between the two disorders in adults. We found only two studies investigating the prevalence and co-occurrence of ADHD in adult patients with epilepsy (from both the same group and survey [Ettinger et al., 2015; Ottman et al., 2011]) and no published study investigating the prevalence of epilepsy in adult patients with ADHD. In their population-based, longitudinal health survey including more than 172,000 adults aged 18 years or more, Ottman et al. (2011) found a prevalence ratio of ADHD of 2.4 (2.0-2.8) among adults with epilepsy relative to a control group without epilepsy. Both the diagnoses of epilepsy and ADHD were based on self-reported lifetime occurrence of the disorders. In a follow-up of these data, Ettinger et al. (2015) investigated the presence and impact of ADHD symptoms in adults with self-reported epilepsy (Ettinger et al., 2015). Using ASRS, they found that 18.4% of adults with epilepsy screened positive for ADHD. A positive screen for ADHD was associated with greater severity of epilepsy (frequency of seizures, more use of anti-epileptic drugs), more comorbidity with anxiety and depression, lower quality of life, and worse functioning/more disabilities in work and social life. A Dutch study found that 2.4% of patients with epilepsy admitted to a special clinic for epilepsy were diagnosed with ADHD (van der Feltz-Cornelis & Aldenkamp, 2006), compared with a 1% prevalence of ADHD in the Dutch population (Kooij et al., 2005).

The comorbidity between ADHD and epilepsy may have diagnostic, prognostic, and treatment implications for both disorders. Central stimulants may theoretically increase seizure susceptibility, although the documentation for this in patients with epilepsy is limited and shows conflicting results (Brown, Becker, Pollard, & Anderson, 2013; Gonzalez-Heydrich et al., 2010). We found only two small studies of methylphenidate (MPH) treatment in adults with epilepsy (Moore, McAuley, Long, & Bornstein, 2002; van der Feltz-Cornelis & Aldenkamp, 2006); none of these demonstrated adverse effects of this treatment.

### Migraine (ICD-10: G43)

Migraine is an episodic headache disorder, with attacks of pain and time-limited neurological dysfunction. Migraine is common in the general population and usually starts in adolescence or early adulthood. The prevalence is approximately 10% to 15%, and females are more often affected than males (Fasmer, Halmoy, Oedegaard, & Haavik, 2011). Thus, compared with ADHD, migraine has a very different profile regarding its prevalence, gender distribution, and age of onset. Both migraine and ADHD have a strong genetic basis, and a similar well-established comorbid connection with both mood and anxiety disorders is found in clinical and epidemiological studies (Fasmer et al., 2012). Cognitive dysfunction is not usually thought to be associated with migraine, apart from changes occurring during acute attacks.

Two large Norwegian studies showed an association between ADHD and migraine. Using data from the Norwegian Prescription Database, a positive and significant association between prescription of anti-migraine and ADHD medication was found for all age groups between 20 and 50 years and for both genders, with ORs ranging from 1.8 to 2.8 (Fasmer et al., 2012).

In a cross-sectional study of adult ADHD patients (*n* = 572) and community controls (*n* = 675), the prevalence of migraine was higher in the patient group compared with the controls (28.3% vs. 19.2%, *p* = .001) (Fasmer, Halmoy, Oedegaard, & Haavik, 2011). The difference from controls was more marked for men (22.5% vs. 10.7%, OR = 2.43, CI = [1.51, 3.90]) than for women (34.4% vs. 24.9%, OR = 1.58, CI = [1.13, 2.21], although not significanlty so. Among the controls, the presence of migraine was associated with higher scores on both ASRS and WURS.

### Sleep Disorders (ICD-10: G47)

ADHD and ADHD symptoms in adults are related to a variety of sleep problems and sleep-related disturbances, both in clinical and non-clinical samples (Boonstra et al., 2007; Fargason, Hollar, White, & Gamble, 2013; Gau et al., 2007; Kass, Wallace, & Vodanovich, 2003; Oosterloo, Lammers, Overeem, de Noord, & Kooij, 2006; Schredl et al., 2007; Surman et al., 2009; Vogel et al., 2015; Walters, Silvestri, Zucconi, Chandrashekariah, & Konofal, 2008; Yoon, Jain, & Shapiro, 2012). Fisher et al. (2014) found that 80% of adults with ADHD reported sleep problems, regardless of sex and ADHD subtype. Sleep problems were more common in adult ADHD than in controls, also when taking psychiatric comorbidity and psychotropic medication into account (Schredl et al., 2007; Surman et al., 2009). Furthermore, persons with sleep problems performed worse on neuropsychological testing for attention (Fisher et al., 2014). Subjectively, patients with ADHD (without current psychiatric comorbidity or ADHD pharmacotherapy) reported worse sleep quality than controls (Philipsen et al., 2005), with more insomnia and problems with the sleep–wake pattern (Schredl et al., 2007). In a clinical sample of ADHD patients without psychiatric comorbidity and denying having insomnia symptoms, the ADHD sample reported more sleep quality problems compared with controls (Fargason et al., 2013). Measured objectively by polysomnography, adults with ADHD showed increased nocturnal activity compared with controls (Kooij, Middelkoop, van Gils, & Buitelaar, 2001; Middelkoop, Van Gils, & Kooij, 1997; Philipsen et al., 2005; Sobanski, Schredl, Kettler, & Alm, 2008), although one study found no difference between the groups (Boonstra et al., 2007). Several studies show that people with ADHD have longer sleep latency than controls (Boonstra et al., 2007; Sobanski et al., 2008), but the results are conflicting (three studies showing no difference between ADHD patients and controls: Kooij et al., 2001; Middelkoop et al., 1997; Philipsen et al., 2005).

Excessive daytime sleepiness affects 37% of adults with ADHD (Oosterloo et al., 2006), and appears to be a predictor of academic and overall functional impairment among students with ADHD (Langberg, Dvorsky, Becker, & Molitor, 2014). Furthermore, sleepiness and inattention can correlate in ADHD patients (Oosterloo et al., 2006). However, a small study by Sangal and Sangal (2004) showed no correlation between self-reported sleepiness and current inattentive symptoms, concluding that sleepiness is not a major contributor to inattention in adult ADHD individuals. It is important to be aware of the possible diagnostic confusion between adult ADHD and hypersomnia or narcolepsy using self-report questionnaires, as there is a high degree of symptom overlap (Oosterloo et al., 2006).

#### Sleep and ADHD subtype

Studies investigating the association between ADHD and comorbid sleep disorders with respect to ADHD subtypes show diverging results. Both inattentive and hyperactive-impulsive ADHD symptoms have been associated with delayed sleep timing (Gamble, May, Besing, Tankersly, & Fargason, 2013). In a study of 62 students diagnosed with ADHD, students with the inattentive subtype did not differ from those with combined subtype on self-ratings of daytime sleepiness (Langberg et al., 2014). However, in two studies with a total of 62 non-medicated patients with ADHD, sleep problems were associated with having the combined ADHD subtype and symptoms of hyperactivity/impulsivity (Mahajan, Hong, Wigal, & Gehricke, 2010; Van Veen, Kooij, Boonstra, Gordijn, & Van Someren, 2010), and hyperactivity alone has been associated with decreased sleep duration (Gau et al., 2007). No significant associations were found between inattention and sleep quality, suggesting that sleep problems are connected with hyperactive-impulsive but not inattentive symptoms (Mahajan et al., 2010). In contrast to these results, which were based on small samples, other studies found that symptoms of inattention were most evidently associated with disturbed sleep, delayed circadian rhythm, and greater sleep need (Bae et al., 2010; Caci, Bouchez, & Bayle, 2009; Gau et al., 2007; Rybak, McNeely, Mackenzie, Jain, & Levitan, 2007; Voinescu, Szentagotai, & David, 2012).

#### Symptom severity

The severity of sleep problems is positively correlated with the number of ADHD symptoms, both among ADHD patients and in the general population (Gau et al., 2007; Mahajan et al., 2010; Schredl et al., 2007), also when taking ADHD comorbidity and medication into account (Schredl et al., 2007). The severity of daytime ADHD symptoms was also associated with the level of sleep problems (Schredl et al., 2007). Daytime sleepiness is associated with increased ADHD severity (Gamble et al., 2013), and is a predictor of academic and overall functional impairment among students with ADHD (Langberg et al., 2014).

#### Insomnia (ICD-10: G47.0)

Insomnia implies dissatisfaction with sleep quantity or quality due to difficulty initiating sleep, maintaining sleep or early-morning awakenings. The symptoms impair daily functioning and affect about 6% to 12% of the adult population when ascertained according to formal diagnostic systems (Pallesen, Sivertsen, Nordhus, & Bjorvatn, 2014). Insomnia is common in people with ADHD; one study showed that 78% of the 40 non-medicated ADHD participants included suffered from sleep-onset insomnia (Van Veen et al., 2010), another study showed that the higher reports of insomnia among ADHD patients compared with controls may be related to the presence of depressive symptoms (Schredl et al., 2007). Sleep-onset insomnia, defined as difficulty getting to sleep at the desired bedtime, is the most problematic sleep problem reported in ADHD (Fisher et al., 2014), and is also a prominent initial side effect of stimulant medication.

#### Circadian rhythm sleep disorder, delayed sleep phase type (ICD-10: G47.21)

Delayed sleep phase syndrome implies a disturbance in the normal circadian rhythm. It is characterized by a preference for late sleep and late rising, with sleep-onset insomnia when trying to get to sleep early and high activity in the late evening/night. The prevalence in the adult general population is estimated at 0.13% to 3.1% (Ando, Kripke, & Ancoli-Israel, 2002; Schrader, Bovim, & Sand, 1993). In a Dutch study by Bijlenga et al., including 202 adults with clinically diagnosed ADHD (18-65 years) and 189 controls, delayed sleep phase syndrome was more prevalent among adult ADHD patients (26%) than among controls (2%; Bijlenga, van der Heijden, et al., 2013). Adults with comorbid ADHD and insomnia were found to have significant circadian rhythm delay, the severity of ADHD symptoms and neuropsychological deficits correlating with the delay (Gamble et al., 2013; Rybak et al., 2007). In contrast to the controls, the patients with adult ADHD had the same prevalence of delayed sleep phase syndrome independent of age, the authors suggesting that delayed sleep phase syndrome in ADHD is not age related (Bijlenga, Van Someren, et al., 2013).

#### Hypersomnia (ICD-10: G47.1 and G47.4)

Central hypersomnias such as idiopathic hypersomnia (G47.1) and narcolepsy (G47.4) cause excessive daytime sleepiness not caused by disturbances in nocturnal sleep or circadian rhythm. In a study including 74 patients with narcolepsy (G47.4) or idiopathic hypersomnia (G47.1), 19% of the affected patients fulfilled the criteria for adult ADHD when using self-report measures (Oosterloo et al., 2006). The overlap between symptoms of hypersomnia and ADHD might lead to misdiagnosis of both diagnoses (Oosterloo et al., 2006). However, both ADHD and hypersomnias are treated using psychostimulant medication, indicating a relation between these disorders (Oosterloo et al., 2006).

#### Sleep-disordered breathing (ICD-10: G47.3 and G47.8)

Sleep-disordered breathing includes a spectrum of sleep-related abnormalities such as upper airway resistance syndrome (G47.8) and obstructive sleep hypopnea syndrome (G47.3), with symptoms such as snoring, episodes of breathing cessation during sleep, and excessive daytime sleepiness. Approximately 13% of men and 6% of women suffer from moderate to severe sleep-disordered breathing (Peppard et al., 2013). Of 78 severely obese adults with ADHD, 56% had sleep apnea (Levy et al., 2009). The cognitive and behavioral symptoms of obstructive sleep apnea such as inattention, poor planning, and restlessness, are similar to symptoms of ADHD (Ball, Wooten, & Crowell, 1999), and treatment may have a positive effect on ADHD symptoms (Youssef, Ege, Angly, Strauss, & Marx, 2011). In a case report of six adults with clinically diagnosed ADHD and impaired sleep quality, all had polysomnographic evidence of sleep-disordered breathing (Surman, Thomas, Aleardi, Pagano, & Biederman, 2006). One study indicated that sleep-disordered breathing symptoms are mainly associated with increased BMI and smoking, and not ADHD symptomatology as such (Schredl et al., 2007). In a Turkish study of 81 treatment-naïve obstructive sleep apnea patients and 32 controls, the prevalence of ADHD symptoms was similar in patients with obstructive sleep apnea and controls (Oguzturk, Ekici, Cimen, Ekici, & Senturk, 2013). One study found a correlation between low oxygen saturation and hyperactivity in patients with sleep-disordered breathing (Sangal & Sangal, 2004). In two small case studies with a total of nine adult ADHD patients, it was observed that treatment for sleep apnea relieved their ADHD symptoms, and some were rediagnosed as having sleep apnea instead of ADHD (Ball et al., 1999; Naseem, Chaudhary, & Collop, 2001). According to these results, sleep apnea may actually be misdiagnosed as ADHD.

#### Periodic limb movements during sleep (ICD-10: G47.61)

In the disorder called periodic limb movements during sleep, contractions of muscles during sleep causes periodic episodes of repetitive limb movements. Unmedicated patients with ADHD show increased periodic limb movements during sleep compared with controls (Philipsen et al., 2005; Sobanski et al., 2008).

#### Impact of stimulant medication

Sleep problems are present in unmedicated adults with ADHD, but stimulant treatment is also associated with dysregulation of sleep. Common initial side effect of stimulant medication is insomnia or delayed sleep-onset latency (Kirov & Brand, 2014; Kooij & Bijlenga, 2013). Atomoxetine may also cause insomnia as an adverse effect (Adler, Liebowitz, et al., 2009). It varies between individuals whether stimulants cause insomnia or not, and sleep problems such as sleep-onset latency may decrease with time as the medication is finished titrated and ADHD symptoms improve (Stein, Weiss, & Hlavaty, 2012). If ADHD medication affects the circadian rhythm, the effect on sleep may be less obvious and appear later (Stein et al., 2012).

Subjectively measured, ADHD participants using methylphenidatereported an improvement in sleep quality (Kooij et al., 2001). A study of the central stimulant lisdexamphetamine including 420 participants showed no difference in global sleep quality among adult ADHD patients receiving lisdexamphetamine compared with placebo, and daytime functioning in the stimulant treatment group improved compared with the adult ADHD group receiving placebo (study not included in the literature search, as it is not a study primarily on comorbidity; Adler, Goodman, Weisler, Hamdani, & Roth, 2009). A clinical study including 80 adult ADHD patients all denying insomnia symptoms (treated with stimulants, *n* = 39; with non-stimulants, *n* = 15 and with no medication, *n* = 26), showed significantly more sleep disturbance and prolonged sleep latency compared with controls (*n* = 25). This result indicated that medical treatment, including stimulant treatment, did not account for the sleep quality problems in the adult ADHD group (Fargason et al., 2013).

Objectively measured, sleep-onset latency increased (Boonstra et al., 2007) and sleep duration (Gamble et al., 2013) was reduced in patients treated with stimulant medication compared with those without such medication, although no change (Kooij et al., 2001) and less sleep latency were also reported (Sobanski et al., 2008). Objectively measured, sleep quality and efficiency improved (Boonstra et al., 2007; Sobanski et al., 2008) in ADHD participants using MPH compared with placebo (Boonstra et al., 2007) or compared with a premedication baseline (Sobanski et al., 2008). Also when adjusted for depression and anxiety symptoms, sleep was more consolidated with less interrupted sleep (Boonstra et al., 2007). Regarding the impact of MPH treatment on nocturnal activity, the results are conflicting: Sobanski et al. (2008) found unchanged number of periodic limb movements during sleep in contrast to Kooij et al. (2001) who found reduced nocturnal activity. Improvements in sleep quality may, however, not be directly related to stimulant medication, as the same proportion (one third) of a total of 831 ADHD participants (*n* = 831) experienced sleep improvement independent of receiving stimulant treatment or placebo (Surman & Roth, 2011).

#### Treatment

ADHD is a 24-hr disease, with symptoms appearing both at day- and nighttime (Stein et al., 2012). Before starting treatment for ADHD, patients should be screened for sleep disorders and sleep patterns, to more easily track changes in sleep associated with stimulant treatment (Stein et al., 2012). Sleep disorders are associated with cognitive impairment, thus ADHD symptomatology may improve if comorbid sleep disorders are adequately treated in addition to specific treatment for ADHD (Schredl et al., 2007). If the patient is using medical treatment for ADHD and has sleep problems, give advice on sleep hygiene and consider reducing the stimulant treatment in the late afternoon, add a small dose of stimulant treatment earlier in the evening or switch to non-stimulant medication (Brown & McMullen, 2001; Hvolby, 2015; Lecendreux & Cortese, 2007). Usually, insomnia as a side effect of stimulant treatment attenuates after 1 to 2 months treatment (Lecendreux & Cortese, 2007). When treating adult ADHD with delayed sleep phase syndrome, one can combine stimulant treatment with exogenous melatonin together with bright light therapy and good sleep hygiene; Kooij et al. describe this treatment in detail (Kooij & Bijlenga, 2013).

### Other Neurological Disorders (ICD-10: Chapter XI)

#### Dementia with Lewy bodies (ICD-10: G31.83)

Symptoms of dementia with Lewy bodies include mental decline, Parkinson-like motor symptoms, sleep disturbances, and hallucinations. In a study from Argentina including patients with Lewy body dementia (*n* = 109), Alzheimer’s disease (*n* = 251), and sex-, age-, and education-matched controls (*n* = 149), previous symptoms of adult ADHD were associated with risk of Lewy body dementia (Golimstok et al., 2011). The prevalence of previous ADHD symptoms was significantly higher than in both the Alzheimer group (OR = 4.9 [2.8, 8.4]) and the control group (OR = 5.1 [2.7, 9.6]). ADHD symptoms were tested according to *DSM-IV* criteria using WURS and ASRS, and in patients with cognitive impairment information was obtained from an informant knowing the patient for at least 10 years. Both ADHD and Lewy body dementia are related to a hypodopaminergic state; this being a possible explanation for the association (Golimstok et al., 2011).

#### Myotonic dystrophy 1 (DM1; ICD-10: G71.1)

Douniol and coauthors described the psychiatric phenotype of the juvenile form of DM1(Douniol et al., 2009), the most common inherited neuromuscular disease, with autosomal dominant transmission. The study included 28 people with juvenile DM1 from 7 to 24 years of age. In the total sample, including both children and adults, 28.6% had ADHD, all inattentive subtypes. ADHD was measured by ASRS in the adults. A study by Echenne et al. describes adult cases with comorbid ADHD and myotonic dystrophy, but it is not known how ADHD was diagnosed or if the participants were tested for ADHD as adults (Echenne et al., 2008).

#### Chronic fatigue syndrome (CFS; ICD-10: G93.3)

CFS is characterized by a combination of prolonged and severe fatigue with non-specific somatic manifestations and cognitive symptoms, including difficulties in concentration, short-term memory and thinking, impaired attention and slow processing speed (Valdizán Usón & Idiazábal Alecha, 2008). These cognitive symptoms may mimic symptoms of ADHD and possibly share some underlying pathophysiological mechanisms (Bellanti et al., 2005). Fatigue symptoms are also commonly reported in adult ADHD and may affect neuropsychological functioning (Fisher et al., 2014).

We found only one study on prevalence of adult ADHD in CFS patients (Sáez-Francàs et al., 2012). In their clinical sample of 158 adults with CFS, 97% women, Sáez-Francàs et al. found that 47 patients (29.7%) fulfilled diagnostic criteria for childhood ADHD assessed retrospectively, and 33 patients (20.9%) were found to still meet criteria for ADHD in adulthood. We found no studies on the prevalence of CFS in samples of adults with ADHD, nor any population-based studies on CFS and adult ADHD; thus, the possible relationship between these conditions and the magnitude of the problem is not clear. Young et al. (2013a) described three female cases with CFS (38-58 years), who were also found to fulfill criteria for ADHD dating back to childhood (Young, 2013a). In all three cases, symptoms of chronic fatigue and/or pain, and general and occupational functioning, improved after treatment with central stimulants.

Despite the limited amount of literature, the suggested association between ADHD and CFS is clinically interesting, as central stimulants, the first-line pharmacological treatment of ADHD, have shown positive effects on both the core symptom of CSF, that is, chronic fatigue (Blockmans, Persoons, Van Houdenhove, & Bobbaers, 2006), and the associated cognitive symptoms, such as executive dysfunction (Young, 2013b).

### Endocrine Diseases (ICD-10: E00-E35)

#### Resistance to thyroid hormone (RTH; ICD-10: E07.8)

RTH usually involves mutations in the thyroid hormone receptor β gene and is often transmitted as an autosomal dominant trait. Classical features include ADHD, tachycardia, and growth delay. Brucker-Davis et al. (1995) described 104 RTH patients and 114 unaffected participants, both children and adults. ADHD was found to be common among the RTH patients; more common in males (72%) than in females (43%). Among adults, 42% had ADHD in the RTH group compared with 4% in the non-RTH group. Full-scale IQ was lower among RTH patients than among controls, and 38% of the patients had IQ less than 1 standard deviation (*SD*) below the mean; however, there was no correlation between IQ and ADHD in the RTH patients.

#### Hypothyroidism (ICD-10: E00-E03)

Hypothyroidism is an endocrine disorder in which the thyroid gland does not produce enough thyroid hormone, leading to a large range of symptoms, including weight gain, fatigue, and poor ability to tolerate cold. In the previously mentioned study by Hodgkins et al., investigating U.S. health care claims for 2006 (adult ADHD: 31,752; non-ADHD: 95,256), hypothyroidism was significantly more common in adults with ADHD compared with those without (*p* ≤ .0001; Hodgkins et al., 2011).

#### Diabetes (ICD-10: E10-E14)

Pancreas insulin cells diabetes mellitus is a heterogeneous group of metabolic diseases characterized by high blood glucose levels over prolonged periods of time. Interestingly, diabetes (ICD-10: E10-E14) was significantly higher in the non-ADHD group compared with the ADHD group in the above-mentioned study investigating U.S. health care claims (*p* ≤ .0001; Hodgkins et al., 2011). However, a Dutch study including older adults with ADHD (*n* = 23) and controls (*n* = 208) found no difference in self-reported diabetes between the individuals with ADHD and controls (Semeijn et al., 2013). Furthermore, a U.S. study including adult patients with ADHD (*n* = 98) and controls (*n* = 100) showed no significant differences in the number of self-reported diabetes (Spencer et al., 2014).

### Metabolic Disorders (ICD-10: E70-E90)

Bijlenga et al. have reported a significantly increased frequency of self-reported metabolic disorders among adults with ADHD compared with controls (Bijlenga, van der Heijden, et al., 2013). We also identified studies describing the co-occurrence of adult ADHD or ADHD symptoms with several different inborn metabolic diseases:

#### Albinism (ICD-10: E70.3)

Albinism is an inherited disorder causing an absence or reduction of melanin in the hair, skin, and/or eyes. The prevalence of albinism worldwide is estimated to 1/17,000 (0.006%), although it varies considerably over different continents (Gronskov, Ek, & Brondum-Nielsen, 2007). In their study of albinism and comorbid ADHD, Kutzbach et al. found that 17 of 75 children (22.7%) and 3 of 44 adults (6.8%) met criteria for ADHD, and that the majority of these had the hyperactive/impulsive subtype (Kutzbach, Summers, Holleschau, King, & MacDonald, 2007).

#### Maple syrup urine disease (MSUD; ICD-10: E71.0)

MSUD is an inborn error of metabolism, with clinical features including neuropsychiatric disturbances and neurologic deterioration. Muelly et al. (2013) studied neuropsychiatric symptoms in 37 patients with MSUD aged 5 to 35 years; 26 treated with diet and 11 with liver transplantation. They found the cumulative lifetime incidence of ADHD to be 54% among MSUD patients on dietary therapy and 82% among patients with liver transplants. They concluded that neurochemical deficiencies correlated with neuropsychiatric morbidity (Muelly et al., 2013).

### Diseases of the Circulatory System (ICD-10: Chapter IX)

Possible increased risk of cardiovascular events due to stimulant treatment of ADHD is an important clinical issue. Long-term (≥12 months) stimulant treatment is associated with increased heart rate and increased blood pressure, but no evidence has so far indicated elevated risk of serious cardiovascular events (Hammerness, Karampahtsis, Babalola, & Alexander, 2015). Although this is a debated issue, only a few studies have investigated the comorbidity of ADHD and cardiovascular disorders per se; none of them focusing on cardiovascular disease alone.

The study by Bijlenga et al. on sleep patterns (202 adult ADHD patients, 189 controls) reported a significantly increased frequency of self-reported cardiovascular disease among adults with ADHD compared with controls (Bijlenga, van der Heijden, et al., 2013). In contrast to this, another Dutch study including older (*M*_age_ = 71.6) adults with ADHD (*n* = 23) and controls (*n* = 208) found no difference in self-reported hypertension and cardiovascular disease between the ADHD individuals and controls (Semeijn et al., 2013). In line with this result, no significant differences between adults with ADHD and controls were found concerning hypertension and other cardiovascular diseases in the previously mentioned study investigating U.S. health care claims for 2006 (31,752 adult ADHD matched with 95,256 non-ADHD individuals; Hodgkins et al., 2011). Furthermore, a U.S. study including adult patients with ADHD (*n* = 98) and controls (*n* = 100) showed no significant difference in the number of self-reported heart attacks (Spencer et al., 2014).

### Atopic Diseases/Allergic Diseases (Primarily ICD-10: Chapter X and Chapter XII)

The results from a systematic review including mainly studies on children concluded that atopic disease in general was not associated with ADHD, but that atopic eczema specifically appears to be independently associated with ADHD (Schmitt, Buske-Kirschbaum, & Roessner, 2010). For further information on atopic eczema, we refer to the paragraph describing skin disorders. Information on the allergic disorders asthma, allergic rhinitis, atopic dermatitis, and allergic conjunctivitis was collected in a study using data from the Taiwan National Health Insurance Research Database from 1996 to 2010. Patients with ADHD (*n* = 5,811; ICD-9-CM diagnosis), patients with tic disorder, patients with comorbid ADHD and tic disorder (*n* = 349), and age/gender-matched controls were retrieved (Chen et al., 2013). Most of the ADHD patients included were adolescents and young adults. Compared with the control group, the ADHD group showed a significantly increased risk of having allergic comorbidities after adjusting for age, gender, and comorbid psychiatric disorders. The comorbid ADHD and tic disorders group showed the highest prevalence of allergic disease. The results pointed to an additive effect of ADHD and tic disorder on the association with allergic comorbidities.

#### Allergic Rhinitis (ICD-10: J 30)

A German study focused on allergic rhinitis, and by collecting information from the German National Health Insurance beneficiaries, 111,394 patients with allergic rhinitis in 2005/2006 were retrieved (Schmitt, Stadler, Kuster, & Wustenberg, 2016). In addition, information on different comorbid disorders was collected, including hyperkinetic disorder (F 90). The results showed that ADHD was more prevalent among those with allergic rhinitis compared with those without, RR = 1.21 [1.13, 1.29]; however, specific information on adults with ADHD was not given.

### Respiratory Disorders ICD-10: Chapter X

#### Asthma (ICD-10: J 46)

Two studies reported an association between unspecific lung diseases and adult ADHD (Bijlenga, van der Heijden, et al., 2013; Semeijn et al., 2013), while there are several studies on adult ADHD and comorbid asthma (Chen et al., 2013; Fasmer, Halmoy, Eagan, Oedegaard, & Haavik, 2011; Fasmer, Riise, et al., 2011; Hodgkins et al., 2011; Karlstad, Nafstad, Tverdal, Skurtveit, & Furu, 2012; Secnik et al., 2005; Spencer et al., 2014). Asthma is an inflammatory disorder of the airways, following a chronic course, but with episodic worsening. Common symptoms are wheezing and coughing, caused by reversible airflow obstruction and bronchospasm (Handoyo & Rosenwasser, 2009). As is the case with ADHD, the disorder usually starts in childhood, and also similar to ADHD, psychiatric disorders, in particular mood and anxiety disorders, are often comorbid problems (Goodwin, Jacobi, & Thefeld, 2003). Tobacco smoking may be another factor that is common to these conditions (Thomson, Chaudhuri, & Livingston, 2004). ADHD patients have a higher smoking prevalence than the general population. It is uncertain if smoking is a cause of asthma, but it aggravates symptoms among people prone to asthma, and passive smoking in childhood and prenatal exposure are associated with an increased risk of asthma (Fasmer, Halmoy, Eagan, et al., 2011).

The relationship between adult ADHD and asthma has been investigated both in clinical samples and using registry data. In a U.S. database search, adults with ADHD were significantly more likely to have a comorbid diagnosis of asthma compared to controls (*p* < .01 (Secnik et al., 2005). Data from the Norwegian Prescription Database showed a higher-than-expected occurrence of ADHD in 20- to 29-year-olds treated for asthma compared with the general population (Karlstad et al., 2012). Similarly, another study using the Norwegian Prescription Database showed that patients prescribed central stimulants were also prescribed anti-asthmatic drugs more often than the remaining population (Fasmer, Riise, et al., 2011). In this study, a weaker relationship between ADHD and asthma was found in the younger age groups (<20 years) than in the older age groups (>20 years), although the associations were significant across all ages. In a cross-sectional questionnaire-based study of 594 adult ADHD patients compared with 719 persons from the general Norwegian population, the prevalence of self-reported asthma was significantly higher in the ADHD group than in controls (24.4% vs. 11.3%). In addition, controls with asthma had higher scores on ratings of ADHD symptoms (Fasmer, Halmoy, Eagan, et al., 2011). These studies point to a comorbidity of ADHD and asthma, apparently most pronounced for adult patients, although none of these four studies adjusted for smoking as a possible confounder.

### Diseases of the Digestive System (ICD-10: Chapter K)

#### Irritable bowel syndrome (IBS; ICD-10: K58)

IBS causes abdominal pain and bloating, and can lead to both diarrhea and constipation. In the previously mentioned U.S. database search by Secnik et al. (2005), 2,252 adults diagnosed with ADHD did not differ significantly from the corresponding large control group in the prevalence of IBS (Secnik et al., 2005). However, the study by Hodgkins et al. (2011), based on U.S. health care claims for 2006 (adult ADHD: 31,752; non-ADHD: 95,256), found that adults with ADHD reported significantly more IBS compared with those without (*p* ≤ .0001; Hodgkins et al., 2011).

#### Celiac disease (CD; ICD-10: K90.9)

CD is an autoimmune disease where the ingestion of the wheat protein gluten leads to damage and subsequent atrophy of the intestinal villi, and thus may compromise nutrient absorption. The primary symptoms are diarrhea, abdominal pain, or discomfort, with weight loss and anemia being common complications. CD is estimated to affect 1% to 2% of the population, with increasing prevalence in later years due to new screening methods and the detection of asymptomatic patients.

Zelnik, Pacht, Obeid, and Lerner (2004) studied the prevalence of several neurological disorders in a sample of 111 young patients with CD (*M*_age_ = 20 years, 42% men), and found that 23 patients (20.7%) had a learning disability (LD) and/or ADHD, compared with 10.5% in a control group without CD, recruited from the same pediatric gastroenterological clinic (Zelnik et al., 2004). Interestingly, the gender distribution of LD/ADHD was very even in the CD group (20.3% females and 21.2% males), whereas male participants were more affected in the control group (12.9% vs. 8.7%), as expected in the general population.

Niederhofer & Pittschieler (2006) found an overrepresentation of ADHD symptoms in patients with CD and investigated possible effects of a gluten-free diet on ADHD symptoms in a sample of patients with CD consisting of both children and adults (*n* = 78, age = 3-57 years [*M* = 19.3]; (Niederhofer & Pittschieler, 2006). Interestingly, although results should be interpreted with caution due to the small sample size and open study design, they found a significant reduction of ADHD-like symptomatology after at least 6 months of gluten-free diet. The reduction of ADHD symptoms further correlated with pain reduction. The same authors also investigated the presence of CD in a primary sample of patients with ADHD (*n* = 67, 52 males, age = 7-42 years [*M* = 11.4]), and found that 10 of the 67 patients were positive for CD (seven males, 13.5%, and three females, 20.0%), defined by the presence of CD-specific antibodies (antigliadine and antiendomysium) in blood serum. A gluten-free diet of at least 6 months was associated with improvement of ADHD symptoms also in this patient sample (Niederhofer, 2011).

### Skin Disorders (ICD-10: Chapter XII)

For unspecific skin disorders, the study by Bijlenga et al. on ADHD and sleep patterns showed no differences in self-reported skin disorders between 202 adult ADHD patients and 189 controls (Bijlenga, van der Heijden, et al., 2013).

#### Atopic dermatitis (ICD-10: L 20)

Atopic dermatitis is a chronic, pruritic inflammatory skin condition characterized by pruritus and red swollen skin. Several studies, mainly on children, have shown a positive association between atopic dermatitis and ADHD symptoms (Gee & Bigby, 2011). For adults, a Turkish study investigating 60 adult patients with atopic dermatitis and 50 non-atopic control participants found significantly more ADHD symptoms in patients with atopic dermatitis than in controls, the association being strongest in females (Cicek et al., 2009). A self-report scale showed that features of inattention, hyperactivity, and impulsivity were all associated with atopic dermatitis, and the authors concluded that co-occurrence of ADHD should be taken into consideration when treating patients with atopic dermatitis.

#### Alopecia areata (AA; ICD-10: L 63)

AA is a likely autoimmune disorder causing hair loss. A register-based study from Taiwan (*n* = 5,117 patients with AA and *n* = 20,468 controls) investigated psychiatric comorbidity in patients with AA and found no association with AA and adult ADHD (Chu et al., 2012).

#### Acne (ICD-10: L70)

Acne is a skin disorder characterized by inflammation of the pilo sebaceous follicle. A registry-based U.S. study including both children and adults showed that ADHD was twice as likely to be associated with acne relative to all other dermatological disorders (Gupta, Gupta, & Vujcic, 2014), also when adjusting for age, sex, atopic dermatitis, anxiety, depression, and stimulant medication. However, there were few participants >18 years.

### Musculoskeletal Disorders (ICD-10: Chapter XIII)

Adults with ADHD report chronic musculoskeletal and skeletal complaints, including fibromyalgia (FMS), more frequently than controls without ADHD (Bijlenga, van der Heijden, et al., 2013; Spencer et al., 2014). Stray and coauthors (2013) investigated motor regulation problems and reported musculoskeletal pain in 25 adults with ADHD (all responders to treatment with MPH) and 23 control individuals. The adults with ADHD scored higher on tests indicating more motor problems than control individuals. As much as 80% of the ADHD patients reported widespread pain; pain level was more severe and more often widespread than in the control individuals. The authors concluded that motor inhibition problems and heightened muscle tone are, as in children with ADHD, increased in adults with ADHD, and that the more widespread and higher pain levels may represent long-term secondary effects of these muscular problems.

#### Rheumatoid arthritis (ICD-10: M05-M06)

In the Dutch study including older adults with ADHD (*n* = 23) and controls (*n* = 208), no difference in self-reported rheumatoid arthritis (ICD-10: M05-M06) was found between the ADHD patients and controls (Semeijn et al., 2013).

#### Systemic lupus erythematosus (SLE; ICD-10: M32)

SLE (ICD-10: M32) is an autoimmune connective tissue disorder where many internal organs in the body, as well as the nervous system, may be affected. Neuropsychiatric symptoms are common, as described in a systematic review by Meszaros and coauthors in 2012 (Meszaros, Perl, & Faraone, 2012). In a recent Chinese study, Gao and coworkers investigated whether SLE patients (*n* = 117) had more ADHD symptoms than healthy age- and sex-matched controls (*n* = 64; Gao, Lo, & Mok, 2015). ADHD symptoms were assessed by the ASRS. Possible ADHD was found in 7.7% of SLE patients and 6.3% of controls (*p* = 1.0); however, SLE patients had more clinically significant items in the inattention domain of the ASRS than the controls (*p* = .006), especially if they had previous cerebral involvement (*p* = .004). Anxiety and depressive symptoms correlated with ADHD symptoms.

N-acetylcysteine (NAC) has been reported to improve psychiatric symptoms in various disorders (Berk et al., 2008; Bernardo et al., 2009). In a randomized- controlled trial, Garcia and coworkers (2013) investigated whether ADHD might serve as a marker for neuropsychiatric disease in SLE patients and as a target for treatment with NAC. They included 49 SLE patients and 46 matched healthy controls, and randomized 24 of the SLE patients to receive placebo or NAC in two dosages. The authors concluded that increased scores on the ASRS indicate previously unrecognized and clinically significant ADHD symptoms that respond to NAC treatment in SLE patients.

#### Fibromyalgic syndrome (FMS; ICD-10: M79.7)

Studies focusing on the comorbidity between adult ADHD and FMS are few, small, and still exploratory in nature. In a sample of 201 women with FMS, 32.3% fulfilled criteria of childhood ADHD, compared with 2.5% in an aged-matched control group of healthy women (Reyero et al., 2011).

Based on clinical reports of adult ADHD with co-occurring fibromyalgic complaints, who experienced relief of their complaints after medication for ADHD, Krause et al. conducted a German pilot study to investigate the comorbidity between ADHD and FMS. Twelve patients with FMS were compared with 12 patients with pain of other origin. The FMS patients had significantly higher symptom scores of ADHD (both past and present) than the other pain patients (Krause et al., 1998).

In a Dutch study including 44 patients with FMS, 11 (25%) of the patients met the criteria for ADHD after being clinically interviewed (Derksen, Vreeling, & Tchetverikov, 2015).

#### Legg-Calve-Perthes disease (LCPD; ICD-10: M91.1)

LCPD is a disease which leads to deformation of the femoral head, is diagnosed in children, and is associated with early hip dysfunction and osteoarthritis of the hip. Hailer and coauthors studied health-related quality of life, physical activity, and behavior patterns in 116 adult patients with LCPD, who had been treated at Uppsala University Hospital between 1978 and 1995 (Hailer, Haag, & Nilsson, 2014). The patients answered self-report questionnaires by interview using ASRS to assess ADHD symptoms. A total of 28% had ASRS scores corresponding to a likely ADHD diagnosis, and a higher ASRS score was associated with a lower score on quality of life questionnaires.

### Congenital Syndromes and Anomalies (Mainly ICD-10: Chapter XVII)

This is a heterogeneous disease entity where various organ systems are affected, either as isolated anomalies with largely unknown etiology occurring sporadically or as multiple anomalies which may or may not be part of known syndromes or associations. The anomalies may be associated with environmental exposures or have well-defined genetic causes. In all instances, ADHD symptoms may be an important feature of the condition, for some genetic syndromes even the presenting feature. If all the clinical criteria of ADHD are fulfilled, it is recommended to separately diagnose this as ADHD, irrespective of its association with other well defined and perhaps underlying illnesses (APA, 2013). Most of the research on syndromes and associated neuropsychiatric disorders such as ADHD and autism is based on children, while we focus on studies where adults are included.

*Tuberous sclerosis (ICD-10: Q85.1)* is an autosomal dominant genetic syndrome associated with neuropsychiatric manifestations such as mental retardation, autism spectrum disorders (ASD), and ADHD (de Vries et al., 2005). ADHD is assumed to be associated with brain lesions due to this disorder (Hunt, 1998). Muzykewicz et al. (2007) reported that 30% of 241 children and adults (average 20 years, range = 8 months - 63.4 years) with tuberous sclerosis had ADHD symptoms (Muzykewicz et al., (2007). A similar fraction of patients had anxiety or depression.

*Chromosomal aberrations* may also be associated with ADHD, as well as with other psychiatric disorders. The *22q11.2 deletion syndrome* (ICD-10: D82.1; velo-cardio-facial [VCFS] or DiGeorge syndrome) is among the most studied genetic syndromes in psychiatry. Whereas high rates of ADHD have been reported in children, psychotic disorders may be the most prominent psychiatric disorders in adulthood (Murphy, 2005); however, psychiatric morbidity in adults is not yet adequately documented (Baker & Vorstman, 2012). The clinical phenotype of this relatively common syndrome (1/2,000-1/4,000 live births) is highly variable. In their comprehensive review of 1,402 participants with VCFS (age = 6-68 years), Schneider et al. (2014) reported that ADHD was the most frequent psychiatric disorder in children (37.1%) and among the most common in adults (15.6%). In contrast to the general population, where the combined type of ADHD is the most common, most cases of VCFS had the inattentive form of ADHD. A similar prevalence of ADHD was found in a smaller study by Tang et al. (2014), where 31% of 112 cases with VCFS (age = 8-45 years, 37% ≥18 years) had ADHD, and 11% had psychosis. There was no significant effect of age on the prevalence of ADHD in this group nor in a study by Niklasson et al. where in-depth neuropsychiatric assessments were done on 100 consecutive patients with VCFS (16% ≥17 years; Niklasson, Rasmussen, Oskarsdottir, & Gillberg, 2009). ADHD was diagnosed in 30 individuals; nine of these also had ASD. Gothelf et al. assessed 51 consecutive patients with VCFS, aged 16 to 30 years (Gothelf et al., 2004). Twenty-one patients (41.2%) were diagnosed with ADHD (*M*_age_ [*SD*] = 11.1 [6.9]), and this group also had a significantly greater prevalence of ADHD among their first-degree relatives. The authors concluded that ADHD in VCFS may have a genetic contribution, and that the VCFS-related developmental factors might play a lesser role.

Both children and adults with *trisomy 21* (Down’s syndrome; ICD-10: Q 90; Capone, Goyal, Ares, & Lannigan, 2006; Edvardson et al., 2014) and *fragile X syndrome* (FXS; ICD-10: Q 99.2; Dorn, Mazzocco, & Hagerman, 1994; Tranfaglia, 2011) have an increased prevalence of behavioral problems and comorbid diagnoses, including ADHD. FXS is the most common hereditary cause of intellectual disability in men and also affects women. Hyperactivity symptoms in FXS usually decline with age (Tranfaglia, 2011). Unlike conventional X-linked disorders, men can be carriers of the syndrome. These carriers (FXS premutation) may have normal intelligence but differ in response inhibition and selective attention, neuropsychiatric symptoms also found in ADHD (Cornish et al., 2008; Dorn et al., 1994). With age, individuals with FXS premutation may develop more severe problems with inhibitory control. A small study from 1994 using a family informant method on 24 daughters of FXS carrier fathers and 32 daughters of control fathers found a significantly higher proportion of adult ADHD as well as other psychopathology among FXS carrier fathers (Dorn et al., 1994). It has been proposed to screen for FXS carrier status in ADHD individuals whose male family members have intellectual disability (Hay, 2008).

As opposed to the mentioned chromosomal aberrations, only hyperactivity symptoms associated with ADHD were found more frequently in *Angelman syndrome (ICD-10: Q93.5)* when compared with a similar control group of individuals with intellectual disability (Berry, Leitner, Clarke, & Einfeld, 2005). *Cornelia de Lange syndrome* (CdLS; *ICD-10: Q87.1*) is a very rare genetic disorder usually caused by de novo mutations. In their study of 69 CdLS patients, Kline et al. (2007) have described their physical and psychiatric disturbances, among which ADHD is one of several psychiatric diagnoses where the symptoms often worsen with age.

Regarding ADHD and comorbid anatomical anomalies that are not part of a well-known syndrome, there are few studies in adults. A large registry-based study from 2012 found an increased risk of ADHD persisting to adulthood in individuals born with oral clefts (ICD-10: Q 35-37; Halmoy, Klungsoyr, Skjaerven, & Haavik, 2012). Another study describing 447 adults with Fallot’s tetralogy (TOF) found an increased prevalence of ADHD among TOF patients who had at least two additional “syndromic” features such as dysmorphic facies, learning disabilities, or voice abnormalities (Piran et al., 2011).

### Enuresis (ICD-10: R32)

The previously described study by Bijlenga et al. showed no differences in self-reported urinary symptoms in people with adult ADHD and controls (Bijlenga, van der Heijden, et al., 2013).

The diagnosis of enuresis in adult ADHD compared with controls is reported in two studies using information from U.S. claim databases. One study included 2,252 individuals diagnosed with ADHD according to ICD-9 during 1999-2001 matched with a similar number of controls (Secnik et al., 2005). Based on ICD-9 codes, there was no significant difference in the prevalence of enuresis between the groups. In a study investigating U.S. health care claims for 2006 (adult ADHD: 31,752; non-ADHD: 95,256), adults with ADHD were significantly more often diagnosed with enuresis compared with adults without ADHD (*p* < .05; Hodgkins et al., 2011).

In a French study including 1,171 adults, ASRS was used to measure adult ADHD. adult ADHD was significantly related to lifetime self-reported enuresis regardless of sex, OR = 5.8 [2.4, 14.1] (Caci et al., 2014).

### Other Disorders

Our search also identified some papers describing various other disorders, summarized in Table 2. For most of these disorders, no significant association with adult ADHD was reported. The exception was for photophobia, where 69% of the ADHD participants reported photophobia compared with 28% in the control group (*p* = .001; Kooij & Bijlenga, 2014).

**Table 2.** Other Comorbid Disorders With Limited Information on the Association With adult ADHD.

Comorbid disorder	Reference	Design	Study population	Result
STD	Hosain, Berenson, Tennen, Bauer, & Wu (2012)	Cross-sectional	462 females (between 18 and 30 years). ASRS to assess ADHD symptoms. Self-reported lifetime diagnosis of STD.	^a^No significant association
Cancer	Bijlenga, van der Heijden, et al. (2013)	Case control	202 clinically assessed adult ADHD patients (*M*_age_ = 34.9), 189 controls (*M*_age_ = 33.0).	No significant association
Cancer	Semeijn et al. (2013)	Case control	23 participants with adult ADHD assessed by semistructured diagnostic interview (mean age 72.0), 208 controls (mean age 68.0).	No significant association
Congenitalesotropia	Olson, Louwagie, Diehl, & Mohney, (2012)	Case control	42 congenitalesotropia patients, 20 controls. Age at ADHD diagnosis not specified	No significant association
Photophobia	Kooij & Bijlenga, (2014)	Online survey	231 people with self-reported ADHD diagnosis/ADHD symptoms (mean age 36.7), 263 controls (mean age 38.4).	Significant association

*Note.* STD = sexually transmitted diseases.

a 10% increased risk of being diagnosed with STD when comparing the ADHD symptom group with the control group, no longer statistically significant when adjusting for sociodemographic covariates.

## Discussion

In our systematic review, we have included 126 papers over the past 20 years mentioning adult ADHD in connection with somatic disease. We found a consistent association between adult ADHD and increased risk of obesity, sleep disorders, and asthma. Associations were also consistent for migraine and celiac disease. Less robust associations have been reported for a number of different disorders such as enuresis, irritable bowel syndrome, restless legs, epilepsy, chronic fatigue syndrome, fibromyalgic syndrome, systemic lupus erythematosus and atopic dermatitis. One large population-based study (adult ADHD: *n* = 31,752) and two smaller studies showed no association between adult ADHD and diseases of the circulatory system (Hodgkins et al., 2011; Semeijn et al., 2013; Spencer et al., 2014). In contrast, a small study by Bijlenga et al. showed an increased risk of self-reported cardiovascular disorder in adult ADHD. Many rare congenital syndromes/malformations, including tuberous sclerosis and FXS, are reported to increase the risk of ADHD. However, under such conditions the ADHD symptoms may also be considered part of the syndrome itself and not a proper comorbid disorder.

We noticed several methodological limitations in the evaluated studies. Most studies were small. Many studies compared a case group with a control/comparison group. However, while the case group might be well defined and characterized, the control group was often less well defined, often based on self-selection and from a different source population than the cases. Information on recruitment and participation rates was sparse, and selection bias was difficult to assess. While the diagnosis of ADHD was often based on clinical interviews or validated self-report questionnaires, the comorbid conditions were sometimes based entirely on a single question to the individual about the condition being present or not. An example is the study by Bijlenga et al., which includes 202 clinically diagnosed ADHD patients and 189 controls non-randomly recruited from students and their acquaintances, and self-selected by posters in libraries and municipal buildings (Bijlenga, van der Heijden, et al., 2013). The numerous somatic diseases described were based solely on self-report from a general health questionnaire.

The larger studies were often based entirely on self-report questionnaires or on registries and databases. Large population-based disease registries may be more suitable to study comorbidity across many different diagnoses, in particular for less prevalent conditions. However, such registries can also be subject to systematic bias, depending on the diagnostic traditions in different countries (Polanczyk et al., 2007) and the covered population.

For details on characteristics of the individual studies with their associated sources of bias, see table in Supplementary 2.

Many studies on psychiatric comorbidity in somatic conditions have explicitly excluded “childhood” psychiatric conditions such as ADHD, as they are not included in commonly used structured psychiatric diagnostic interviews, such as The Structured Clinical Interview for *DSM-IV* Axis I Disorders (SCID-I; First, Spitzer, Gibbon, & Williams, 2002) or The Mini-International Neuropsychiatric Interview (MINI; Sheehan et al., 1998). This systematic bias could give the false impression that ADHD is not a relevant psychiatric comorbidity in somatic disease among adults.

As mentioned in many of the cited articles, there are many reasons to systematically search for psychiatric disorders in somatic conditions and to screen for somatic diseases among psychiatric patients. For the individual patient, it is important to detect potentially treatable somatic diseases masking as a psychiatric disorder. The utility of this approach was illustrated in a recent report, showing that metabolic screening of individuals with psychosis revealed treatable metabolic disorders in a significant number of cases (Demily & Sedel, 2014).

ADHD is a prevalent condition, and patients with many different somatic conditions may exhibit ADHD symptoms either as a comorbid condition or as part of the somatic disease. Likewise, the lifestyle of ADHD patients may make them more vulnerable to certain somatic diseases. Thus, the identification of other treatable and possibly underlying conditions should be addressed in every diagnostic workup.

The presence of a known co-occurring somatic condition has important implications for ADHD treatment. For instance, stimulant therapy may be contraindicated or need careful monitoring in the presence of cardiac disease, hypertension, glaucoma, or liver failure (Kooij, 2012). However, stimulant therapy may have positive effects on the treatment of obesity, sleep disorders, CFS, and restless legs.

### Pathophysiology

Conditions classically thought to be disorders of the nervous system may also include alterations in other physiological systems, for example, involving immunological or endocrine signaling mechanisms (Qureshi & Mehler, 2013). Classical quantitative genetic studies, such as twin studies, and more recently genome-wide association studies with polygenic analyses have revealed genetic correlations between many different psychiatric and somatic disorders and traits. Elucidation of such shared genetic mechanism could lead to new therapies or diagnostic procedures. There is increasing evidence for inflammatory and autoimmune mechanisms in psychiatric disorders, including ADHD. In a recent epidemiological study of 48,000 Norwegian ADHD patients, a strong connection between immunological diseases in the mothers and ADHD in the offspring was observed (Instanes et al., 2015).

Different hypotheses regarding underlying pathophysiological mechanisms are further elaborated for some of the most commonly associated diseases.

Several mechanisms may account for the reported association between ADHD and obesity. Shared neurobiological or genetic mechanisms may be common to both ADHD and obesity (Nigg, 2013); ADHD and obesity being facets of the same underlying condition (Odent, 2010). Both ADHD and obesity are related to the dopamine system, and dopamine-related genes may affect body weight, eating patterns, and ADHD (Davis, 2009). It has been hypothesized that the urge for food intake can share the same mechanisms as ADHD-drug abuse (Cortese et al., 2007; Davis, 2010). Low tonic dopamine levels in the prefrontal cortex may lead to overeating as a kind of self-medication to increase the dopamine levels (Campbell & Eisenberg, 2007). Hypersensitivity to reward contributes to overeating because of an increased motivation in engaging in pleasurable activities (Davis, 2009). Other possible mechanisms involve the Brain Derived Neurotrophic Factor (Cortese & Morcillo-Peñalver, 2010), immune or inflammatory response (Nigg, 2013), and the melatonin system (Cortese & Morcillo-Peñalver, 2010; Kirov & Brand, 2014). A mediation analysis conducted in a clinical sample including 114 obese people, 202 adult ADHD patients, and 154 controls showed that both sleep duration and unstable eating patterns mediated the association between BMI and ADHD after controlling for current anxiety/depression and sociodemographics (Vogel et al., 2015).

Obesity and disorders related to obesity could also create symptoms resembling ADHD symptoms, such as sleep-disordered breathing, which can lead to inattentive symptoms during the day (Cortese & Morcillo-Peñalver, 2010). ADHD itself can lead to obesity rather than the other way around (Cortese, Ramos-Olazagasti, et al., 2013). The behavior associated with ADHD can lead to bad eating habits due to poor planning of the meals (Davis, 2009), or deficient inhibitory control or aversion to delay leading to increased consumption of palatable fast food (Cortese, Angriman, et al., 2008; Davis et al., 2006). Impulsivity associated with binge eating may contribute to impulsivity as a symptom of ADHD in obese patients, and abnormal eating behaviors may lead to symptoms of inattention and hyperactivity (Cortese, Angriman, et al., 2008). ADHD may also be characterized by motor clumsiness and poor energy regulation, resulting in periods of overactivity interspersed with underactivity, making it difficult for persons with ADHD to be a part of activities promoting fitness and weight loss that requires planning and sustained effort (Nigg, 2013). Positive correlations between symptoms of adult ADHD measured by ASRS, depression, anxiety, and disordered eating pattern have been shown (Alfonsson et al., 2012).

The cause of sleep problems in ADHD appears to be multifactorial and complex (Kirov & Brand, 2014), and the association between ADHD and sleep problems may be caused by different underlying pathways. It is not fully understood how obstructive sleep apnea, periodic limb movements during sleep, and RLS are connected to ADHD pathophysiology, or whether they can be viewed as comorbid sleep disorders in ADHD (Kirov & Brand, 2014). RLS share features with ADHD, and RLS and ADHD may be a part of the same symptom complex and share a central nervous dopaminergic dysfunction (Philipsen, Hornyak, & Riemann, 2006). As for ADHD, altered dopaminergic signaling and iron deficiency have been hypothesized to contribute to the pathophysiology of RLS (Cortese et al., 2005; Cortese, Lecendreux, et al., 2008). Hyperactive symptoms may directly cause sleep problems (Hvolby, 2015), and persons with ADHD can be more vulnerable to the effects of sleep disturbances (Hvolby, 2015). In itself, sleep problems may mimic ADHD symptomatology (Hvolby, 2015) and may also exacerbate underlying ADHD symptoms (Owens, 2005). Insomnia may cause inattention, a core symptom of ADHD (Voinescu et al., 2012). Without a thorough assessment, persons with sleep problems could be misdiagnosed as having ADHD (Gau et al., 2007). Furthermore, the stimulant treatment used for ADHD may result in sleep problems as a side effect (Owens, 2005). Common pathophysiology can lead to both ADHD and sleep disturbance (Brown & McMullen, 2001; Hvolby, 2015). Furthermore, a person with a delayed circadian rhythm may compensate being tired by binge eating during the day, which again leads to obesity (. Kooij & Bijlenga, 2013). A delayed sleep phase and sleep deficit can be risk factors for several disorders such as obesity, diabetes, and cardiovascular disorders (Kooij & Bijlenga, 2013).

As for ADHD, the dopaminergic system has also been suggested as a possible factor involved in the pathophysiology of asthma, as dopaminergic receptors are present in sensory nerves in the airways, and inhaled dopamine may induce bronchodilation during asthma attacks (Birrell et al., 2002; Cabezas, Lezama, & Velasco, 2001). Other common pathophysiological factors for these disorders may be inflammatory mechanisms (Barrios, Kheradmand, Batts, & Corry, 2006) and obesity (Cortese, Angriman, et al., 2008; Delgado, Barranco, & Quirce, 2008), as obesity leads to a proinflammatory state (Bazar, Yun, Lee, Daniel, & Doux, 2006).

There is substantial evidence that dopaminergic mechanisms are involved in migraine. It is therefore possible that changes in dopaminergic systems may represent common etiological factors for ADHD and migraine (Fasmer, Akiskal, Kelsoe, & Oedegaard, 2009).

### Suggestions for Further Research

A number of shortcomings have been identified in the literature cited in this review.

1. Different research designs have been utilized to explore the relationship between ADHD and somatic health. As each of these designs has their inherent limitations, there is a need to establish the prevalence of somatic diseases in representative samples of ADHD.
2. In many studies, the patients were simply screened for ADHD symptoms. We need to know what proportion of these patients has the full clinical syndrome, including impairment criteria. Harmonization of diagnostic protocols should increase reliability of such data.
3. It is suspected that many of the reported associated conditions are due to recognized or previously unknown confounders. Future research should investigate how much of the comorbidities are actually due to confounding factors, for example, that headache or sleep disorders may be confounding factors for the association between obesity and ADHD (Cortese & Angriman, 2014; Gau et al., 2007), or that iron deficiency in ADHD is explained by, at least partly, the elevated presence of obesity (Cortese & Angriman, 2014).
4. Many of the existing studies are small. To be able to reveal a true association between ADHD and comorbid somatic diseases with adequate control for potential confounders, large studies are needed. The possibilities of combining the use of population-based registries and common diagnostic protocols should be investigated.
5. From a practical perspective, we need to know how current diagnostic and treatment algorithms should be optimized to account for coexisting conditions.
6. Access to biomarkers from large samples of ADHD cases and its comorbid conditions will allow systematic studies of their shared pathophysiology. This information should also inform future decisions regarding diagnoses and treatment.
7. Even for the best documented conditions, the literature was dominated by a few authors, research groups, and study populations. More research should be conducted in different geographical areas and ethnic groups.

## Supplementary Material

Supplementary material
