# Supplementary material for: Adult ADHD and Comorbid Somatic Disease: A Systematic Literature Review
Source: J Atten Disord. 2016 Sep 1;22(3):203–28. doi: 10.1177/1087054716669589 (PMC5987989; doi:10.1177/1087054716669589)
Supplement: Supplementary material [file Supplementary_2.pdf]

"Adult ADHD and comorbid somatic disease. A systematic literature review. "

Supplementary 3.

SUPPLEMENTARY 1

Search strategies.

Search Strategy **MEDLINE 8.12.2014:**

1 Attention Deficit Disorder with Hyperactivity/ (21710)

2 (adhd or addh or attention deficit disorder or hyperkinetic disorder).tw. (17126)

3 1 or 2 (25533)

4 exp "diseases (non mesh)"/ (12226658)

5 Epidemiologic studies/ or exp case control studies/ or exp cohort studies/ (1604084)

6 Case control.tw. (85883)

7 (cohort adj (study or studies)).tw. (100884)

8 Cohort analy$.tw. (4268)

9 ((follow up or follow-up or followup) adj (study or studies)).tw. (40272)

10 (observational adj (study or studies)).tw. (51544)

11 (Longitudinal or retrospective or cross sectional).tw. (626298)

12 Cross-sectional studies/ (193902)

13 5 or 6 or 7 or 8 or 9 or 10 or 11 or 12 (2045054)

14 3 and 4 and 13 (2566)

15 limit 14 to "all adult (19 plus years)" (834)

16 limit 14 to "all child (0 to 18 years)" (2263)

17 15 and 16 (603)

18 16 not 17 (1660)

19 14 not 18 (906)

20 "review"/ (1985863)

21 review.tw. (994783)

22 20 or 21 (2395613)

23 19 and 22 (120)

24 3 and 4 and 22 (1922)

25 limit 24 to "all adult (19 plus years)" (356)

26 limit 24 to "all child (0 to 18 years)" (1210)

27 25 and 26 (280)

28 26 not 27 (930)

29 24 not 28 (992)

"Adult ADHD and comorbid somatic disease. A systematic literature review. "

Supplementary 3.

Search Strategy **PsycINFO 8.12.2014:**

1 exp attention deficit disorder/ (19335)

2 (adhd or addh or attention deficit disorder or hyperkinetic disorder).tw. (20785)

3 1 or 2 (23135)

4 exp congenital disorders/ or exp feeding disorders/ or exp physical disorders/ (404949)

5 exp symptoms/ (173417)

6 4 or 5 (507138)

7 3 and 6 (7838)

8 limit 7 to "300 adulthood <age 18 yrs and older>" (2144)

9 limit 7 to (100 childhood <birth to age 12 yrs> or 200 adolescence <age 13 to 17 yrs>) (5309)

10 8 and 9 (1112)

11 9 not 10 (4197)

12 7 not 11 (3641)

13 limit 12 to ("0430 followup study" or "0450 longitudinal study" or "0451 prospective study" or "0453 retrospective study") (238)

14 clinical trials/ (8138)

15 longitudinal studies/ (14979)

16 retrospective studies/ (347)

17 cohort analysis/ (1061)

18 Prospective Studies/ (438)

19 (Cohort adj (study or studies)).mp. (10998)

Supplementary 3.

20 (Case control adj (study or studies)).mp. (4806)

21 followup studies/ (12314)

22 ((follow up or follow-up or followup) adj (study or studies)).tw. (11745)

23 (Family adj (study or studies)).mp. (2806)

24 (observational adj (study or studies)).tw. (5761)

25 (epidemiologic$ adj (study or studies)).tw. (10301)

26 (cross sectional adj (study or studies)).tw. (13481)

27 14 or 15 or 16 or 17 or 18 or 19 or 20 or 21 or 22 or 23 or 24 or 25 or 26 (90626)

28 12 and 27 (166)

29 13 or 28 (343)

30 limit 29 to ("0200 book" or "0240 authored book" or "0280 edited book" or "0300 encyclopedia" or "0400 dissertation abstract") (35)

31 29 not 30 (308)

32 review.mp. [mp=title, abstract, heading word, table of contents, key concepts, original title, tests & measures] (303998)

33 12 and 32 (420)

34 33 not 30 (418)

35 limit 34 to yr="1994 -Current" (366)

"Adult ADHD and comorbid somatic disease. A systematic literature review. "

Supplementary 3.

Search Strategy EMBASE 26.01.2015:

1 attention deficit disorder/ (38493)

2 (adhd or addh or attention deficit disorder or hyperkinetic disorder).tw. (21522)

3 1 or 2 (40160)

4 exp physical disease/ (15651306)

5 3 and 4 (24757)

6 limit 5 to (adult <18 to 64 years> or aged <65+ years>) (5041)

7 limit 5 to (embryo <first trimester> or infant <to one year> or child <unspecified age> or preschool child <1 to 6 years> or school child <7 to 12 years> or adolescent <13 to 17 years>) (12157)

8 6 and 7 (2191)

9 7 not 8 (9966)

10 5 not 9 (14791)

11 Clinical study/ or Case control study/ or Family study/ or Longitudinal study/ or Retrospective study/ (633963)

12 Prospective study/ (271121)

13 Randomized controlled trials/ (63501)

14 12 not 13 (269332)

15 Cohort analysis/ (186105)

16 (Cohort adj (study or studies)).mp. (127928)

17 (Case control adj (study or studies)).tw. (79630)

18 ((follow up or follow-up or followup) adj (study or studies)).tw. (48562)

19 (observational adj (study or studies)).tw. (70739)

20 (epidemiologic$ adj (study or studies)).tw. (77107)

21 (cross sectional adj (study or studies)).tw. (93580)

22 cross-sectional study/ (128813)

23 11 or 14 or 15 or 16 or 17 or 18 or 19 or 20 or 21 or 22 (1346755)

24 10 and 23 (1274)

25 limit 24 to (book or book series or conference abstract) (187)

26 24 not 25 (1087)

27 review.mp. [mp=title, abstract, subject headings, heading word, drug trade name, original title, device manufacturer, drug manufacturer, device trade name, keyword] (2770704)

28 10 and 27 (5006)

29 limit 28 to (book or book series or conference abstract) (228)

30 28 not 29 (4778)

31 limit 30 to yr="1994 -Current" (4668)

32 *attention deficit disorder/ (21353)

33 (th or dt).fs. (3887116)

34 31 and 32 (1384)

35 34 not 33 (443)

Supplementary 2

"Adult ADHD and comorbid somatic disease. A systematic literature review. "

Supplementary 3.

Study characteristics of individual studies included in the systematic review. The table only describes somatic and psychiatric present review-article, i.e. the individual studies may include more outcomes.
For abbreviations on assessment tools (*) see supplementary 3.

Ref.	Country	Study design & selection	Total number	Somatic comorbid disorder	Adult ADHD	
			N	Diagnosis	N	Diagnosis
Brook et al., 2013 (Brook, Brook, Zhang, Seltzer, & Finch, 2013)	USA	Prospective, community based cohort study following adolescents into adulthood. Participation rate for original invitation not given. Adolescents assessed: 756; where 72.9% were followed to adulthood and had ADHD information	551	**General physical health:** assessed with self-report questionnaire.	72	Baseline: Assessment o ADHD in adolescence b structured interview. *DSM-III* criteria for AD
Hodgkins et al., 2011 (Hodgkins, Montejano, Sasane, & Huse, 2011)	USA	Retrospective analysis of health care claims and employer-rated health and productivity management databases in 2006. Adult individuals with ADHD were matched with non-ADHD controls and a group of patients with depression.	156,973	**General physical health:** Annual direct health care cost assessed by the utilization and expenditure data. Indirect costs: Absence from work, short-term disability and and workers' compensation. "Comorbidity burden" (Charlson Comorbidity Index) based on diagnosed medical/physical conditions and non-diagnostic claims during 2006 for obesity, diabetes, hypothyroidism, hypertension, other cardiovascular disease, asthma, enuresis and irritable bowel syndrome.	31,752	Criteria: At least 1 evaluation & manageme psychiatry claim with ar ADHD diagnosis in 200 2007; at least 1 confirm ADHD diagnosis withir next 12 months; evidenc continuing treatment for ADHD in 2006; and continuous enrollment i health plan with pharma benefits in 2006. Diagno based on *ICD-9-CM*.
Secnik et al., 2005(Secnik, Swensen, & Lage, 2005)	USA	Registry based matched case control study based on linked data from medical claims databases providing information on medical history and work.	Comorb-idity: 4504. Work and costs: 708	**General physical health:** Direct medical costs and productivity costs related to missed work days. Asthma; enuresis; irritable bowel syndrome. Based on registered ICD-9 diagnoses.	Co-morbidity: 2252 Work and costs: 354	Registered ICD-9 code the medical claims datal
Spencer et al., 2014 (Spencer, Faraone, Tarko, McDermott, & Biederman, 2014)	USA	Case control study. Selection of potential cases through referrals to authors' clinical programs, -participation rate not given. Self-selection of controls through advertisements in the greater Boston area.	198	**General physical health:** Health status based on self-reported history of diabetes, heart attacks, asthma, musculoskeletal complaints including fibromyalgia, and others. Health risk indicators based on self-report and clinical measurements. "Bad health habits" as measured by the Behavioral Risk Factor	98	Structured clinical inter for *DSM-IV* by trained l interviewers, blind for ADHD status. ASRS*.

				Surveillance System.		
Hosain et al., 2012 (Hosain, Berenson, Tennen, Bauer, & Wu, 2012)	USA	Cross sectional study based on baseline interviews of an ongoing longitudinal study among low-income women from family planning clinics. Eligible women: 885; Included: 462 (52%) without drug and alcohol abuse	462	**Sexually transmitted infections (A50-A64):** Interview assessment, using Sexual Risk Behavior Assessment Schedule.	N/A	ASRS*
Brucker-Davis et al., 1995) (Brucker-Davis et al., 1995)	USA	Case-control study. Cases:104 hospitalized patients with resistance to thyroid hormone (RTH) from 42 unrelated families . Controls: 114 unaffected relatives, including 29 persons married into families with RTH.	218 including 113 adults	**Resistance to thyroid hormone (E07.8):** Diagnosed by blood sample, and confirmed by DNA analysis.	Number not specified. Among adults with RTH: 42% Among adults without RTH: 4%	Diagnosed by psychiatr blind to the RTH diagno using appropriate psych interviews.
Hodgkins et al., 2011 (Hodgkins et al., 2011)	USA	Retrospective analysis of health care claims and employer-rated health and productivity management database in 2006; see above.	156,973	**Hypothyroidism (E00-E03) and Diabetes (E10-E14):** Information from databases, see above.	31,752	Diagnosis based on *ICD CM*; see above.
Semeijn et al.,2013 (Semeijn et al., 2013)	The Nether-lands	Case control study based on an ongoing longitudinal study with randomly selected samples from population registries. Response rates varying over phases. N=1494 screened for ADHD, 271 randomly selected for interview, 231 included in study.	231	**Diabetes (E10-E14):** Respondents asked about current diseases e.g.: cardiac diseases, hypertension, diabetes mellitus, chronic nonspecific lung disease, rheumatoid arthritis, cancer. If positive response, more detailed questions from a general health questionnaire. Information on chronic diseases also from general practitioners.	23	Hyperactivity disorder (ADHD) Screening List screening ADHD in old adults Aged 60-94 years DIVA*
Spencer et al., 2014 (Spencer et al., 2014)	USA	Case control study; see above	198	**Diabetes (E10-E14):** Self-reported history of diabetes; see above.	98	Structured clinical inter for *DSM-IV*, and ASRS see above
Alfonsson et al., 2012 (Alfonsson, Parling, & Ghaderi, 2012)	Sweden	Study based on self-report questionnaires. Patients referred to bariatric clinic invited. Participation rate 86%.	187	**Obesity (E66):** Measured height and weight.	19 (10.2%) with likely ADHD	ASRS-S*
Altfas et al., 2002 (Altfas, 2002)	USA	Retrospective, systematic review of clinical records of all patients treated for obesity at one bariatric clinic in 2000.	215	**Obesity (E66):** All patients were referred to bariatric clinic. Measured height and weight, grouped in BMI categories	ADHD: 59 (27.4%), all inattentive type. ADHD symptoms:	Semi-structured clinical interview according to *I IV* criteria

					72 (33.5%)	
Anderson, 2006 (Anderson, Cohen, Naumova, & Must, 2006)	USA	Prospective, community based cohort study (Children in the Community Study), following children (age 9.1-16.6 years) to adulthood (age 27.7-38.3 years). Participation rate not given.	655	**Obesity (E66):**<br>BMI from reported weight and height at each evaluation point. Standardized to BMI Z-scores.	466 (71.1%)	In-home structured, diagnostic interview by trained lay interviewers ages < 16.6 years, consi with *DSM IV* criteria fo ADHD, oppositional de disorder and conduct disorder. No specific information on adult AI
Biederman, 2010 (Biederman, Spencer, Monuteaux, & Faraone, 2010)	USA	Two longitudinal case-control studies, (boys and girls). Cases (ADHD patients) selected 1) from consecutive referrals to pediatric psycho-pharmacology unit, and 2) consecutive pediatric outpatients where patient records ascertained ADHD.<br>Controls were from outpatients receiving routine physical check-ups. Participation rates not given.	Baseline: 522,<br>At 10 years follow up: 404 (77.4%)<br>At follow up with growth data: 261 (50%)	**Obesity (E66):**<br>BMI: Measured weight and height at each wave.<br>Converted to standardized weight, height and BMI Z-scores	Baseline: 280	Consecutive children referred for ADHD to outpatient clinics. Diagn confirmed by telephone questionnaires to mothe then structured intervie trained interviewers, usi K-SADS-E* and SCID* Diagnosis based on *DSI* Diagnostic uncertainties resolved by psychiatrist Persistent ADHD if full subthreshold *DSM-IV* criteria met at last mont before 10 year follow up
Bijlenga et al., 2013 (Bijlenga, van der Heijden, et al., 2013)	The Nether-lands	Cross sectional analysis comparing ADHD patients with a control group.<br>ADHD cases recruited from an out-patient clinic for adult ADHD individuals. Controls mainly self-selected from public institutions, and a group of students "and one of their acquaintances".<br>Participation rate not given.	391	**Obesity (E66):**<br>BMI based on reported height and weight.<br><br>Self-report questionnaires and checklists covering:<br>Sleep, metabolic disorder, cardiovascular disorder, respiratory disorder, digestive system disorder, immune system disorder, skin disorder, skeletal disorder, urinary system disorder, cancer.	202	Diagnosed by trained psychologist/ psychiatri outpatient clinic for adu ADHD, using DIVA*. A met *DSM-IV* criteria.<br>ADHD combined type: 83.2%<br>ADHD inattentive type: 16.3%<br>ADHD hyperactive/impulsive ty 0.5%
Caci et al., 2014 (Caci, Morin, & Tran, 2014)	France	Parents of youths participating in a large study on ADHD symptoms in the community were asked to fill in questionnaires, response rate not given.	1171	**Obesity (E66):**<br>BMI based on reported height and weight.	ADHD defined by the 2-phase method: 2.99%	ASRS* filled in and sco in three ways: 6-item screener, all 18 items an screener followed by the remaining items (2-phas The 2-phase method use define an ADHD group
Cortese et al., 2014 (Cortese, Faraone, Bernardi, Wang, & Blanco,	USA	Cross sectional survey data from the National Epidemiologic Study on Alcohol and Related Conditions, a survey from a large, representative sample of the US population.	34,653	**Obesity (E66):**<br>BMI based on reported height and weight.	Persistent: 340<br>Remitted: 275	Face-to-face interviews experienced lay intervie with extensive training a supervision; using the Alcohol Use Disorder a Associated Disabilities Interview Schedule - *DS*

2013)						*IV* Version. ADHD diagnosed after *DSM-IV* criteria, but with sympto debut < 12 years of age. Remittent, persistent an lifetime ADHD assessed
Cortese et al., 2013 (Cortese, Ramos Olazagasti, et al., 2013)	USA	Longitudinal study. Cases: boys diagnosed at research clinic with ADHD, combined type, without comorbid behavior problems. Matched controls without ADHD and conduct disorder from same clinic. Reassessed as adults, included when height and weight measures present.	222	**Obesity (E66):** BMI based on reported height and weight.	Persistent: 24 Remitted: 87	Childhood ADHD meet the *DSM-IV- TR* criteria combined type. Adult ADHD defined as meeting the *DSM-IV-TR* criteria using The Assessment of Adult Attention Deficit Hyperactivity Disorder, SCID 1* and PRISM* clinicians blind to child diagnosis.
Cortese et al., 2015 (Cortese et al., 2015)		A systematic review including 42 studies describing the degree of association between overweight/obesity and ADHD, 41 of these included in a meta-analysis describing an association between obesity and ADHD.	728,136	**Obesity (E66):** Obesity defined either by 1) self-report or medical record diagnosis, 2) BMI based on reported or measured height and weight.	Total: 48161 Adults: 2046	1) ADHD as defined by *DSM* or hyperkinetic disorder as defined by *I* 2) scores above a sympt threshold on a validated ADHD rating scale, 3) a positive answer to the question: "did your doct ever tell you that you ha ADHD? 4) a medical re diagnoses of ADHD.
Davis et al., 2009 (Davis et al., 2009)	Canada	Case control study where cases with binge eating disorder BED) and controls were self-selected through posters in public institutions and newspaper ads. All were screened by telephone interview. Participation rate not given.	181	**Obesity (E66):** BMI based on measured height and weight.<br><br>Binge eating disorder based on modified *DSM-IV-TR* criteria. Telephone and structural clinical interview.	N/A	CAARS*, WURS*.
de Zwaan et al., 2011(de Zwaan et al., 2011)	Germany	Cross-sectional study with a representative sample of the general population. Cases with self-reported ADHD symptoms compared with a control group. Response rate 61.9%.	1633	**Obesity (E66):** BMI based on reported height and weight.	77	Self-reported: WURS* (short version) and ADH SR*.
Docet et al., 2010 (Maria F Docet, Larranaga, Fernandez Sastre, & García-Mayor,	Spain	Case-control study. Cases: obese attending a nutrition clinic. Controls: normal weight adults attending a pharmacy. Refused participation: 1.2% obesity group and 3.9% in the normal weight group.	243	**Obesity (E66):** BMI based on measured height and weight.	40	A symptom questionnai based on the *DSM-IV* cr and ASRS*, screener an positive full version.

2010)						
Docet et al., 2012 (M. F. Docet, Larranaga, Perez Mendez, & Garcia-Mayor, 2012)	Spain	Case-control study. Participants attending a nutrition clinic. Cases: positive screening for ADHD. Controls: negative screening for ADHD.<br>Refused participation: 1.9% for ADHD and 0.5% for nonADHD.	230	**Obesity (E66):**<br>Abnormal eating behaviors assessed by an eating pattern questionnaire during a clinical interview.	51	ASRS* administered du a clinical interview.
Fleming et al., 2005 (Fleming, Levy, & Levitan, 2005)	Canada	Prospective study of consecutive obese women referred to an obesity clinic.<br>33% drop-out.	75	**Obesity (E66):**<br>BMI ≥35. Details on assessment not given.	20	WURS*, CAARS*, BADDS*
Levy et al., 2009(Levy, Fleming, & Klar, 2009)	Canada	Longitudinal clinical intervention study over 466 days. A consecutive sample of severely obese patients referred to a medical specialist to treat refractory obesity, all evaluated for ADHD. The ADHD group divided into those using ADHD medication and those not.	242	**Obesity (E66):**<br>BMI based on measured height and weight, self-reported for 11 controls.<br>**Sleep apnea:** chart records and overnight sleep.	78	WURS*, ASRS*, clinic interview.
Nazar et al., 2014 (Nazar et al., 2014)	Brazil	Cross-sectional study. Clinical sample of obese women with eating disorder, both with and without ADHD. 22.8% were excluded from the sample of 171	132	**Obesity (E66):**<br>No information on measured or self-reported height and weight.<br>Eating disorders diagnosed by the Eating Disorders Module of the SCID-P.<br>Binge Eating Scale, BIS, Beck Depression Inventory	40	K-SADS* module for ADHD, adapted to adul
Nigg et al., 2016 (Nigg et al., 2016)	USA	Meta-analysis of 43 population based or case-control studies investigating an association between obesity and ADHD.	703,937	**Obesity (E66):**<br>Overweight and obesity defined by BMI.	Total: 69,669<br>Adult ADHD ≥ 18 years: no info.	Based on *DSM* criteria, measure of ADHD symptoms using a valid ADHD symptom rating scale, or in epidemiolog studies, identification of ADHD using a diagnost specific question, or by chart review.
Pagoto et al., 2009 (Pagoto et al., 2009)	USA	Cross-sectional analysis of data (2001-03) from two population based national representative surveys comparing cases (childhood or current ADHD) with controls (non- ADHD).<br>Response rate: Survey 1:71%, survey 2: 72%.	6735	**Obesity (E66):**<br>BMI based on reported height and weight.<br>Binge eating disorder: CIDI* (*DSM-IV* criteria with adjustments).	Childhood ADHD: 492<br>Adult ADHD: 243	Retrospective assessme childhood ADHD: retrospective version of *DSM-IV*.<br>Those with childhood symptoms assessed for aADHD by clinical interviews using ACDS ADHDRS–IV* and an adaptation ADHDRS–I

						Major depressive disord CIDI* (*DSM-IV* criteria SCID* for clinical reappraisal.
Pagoto et al., 2010 (Pagoto et al., 2010)	USA	Cohort study. All patients completing a 16-week behavioral weight loss program at a medical center invited to fill out questionnaires on ADHD and eating habits. Chart review pre- and post-treatment. Response rate: 40.6%	63	**Obesity (E66):** Information on height and weight pre- and post- treatment was retrospectively collected from medical charts.	19	ASRS
Semeijn et al.,2013 (Semeijn et al., 2013)	The Nether-lands	Case control study; see above.	231	**Obesity (E66):** BMI based on measured height and weight.	23	Screening list and DIVA see above
Strimas et al., 2008 (Strimas et al., 2008)	Canada	Self-report questionnaires and height and weight measurements from a group of healthy adult males. No information on how or from where participants were recruited. Response rate not given.	145	**Obesity (E66):** BMI based on measured height and weight. Overeating assessed by questionnaires: the Depression subscale of the Emotional Eating Scale, the Emotional Eating and External Eating subscales of the Dutch Eating Behavior Questionnaire and the Bingeing subscale of the Binge Eating Questionnaire.	N/A	WURS*, CAARS*, BIS
Vogel et al., 2015(Vogel et al., 2015)	The Netherlands	Case control study. Obese patients recruited from outpatient clinics for obesity and eating disorder, and from a lifestyle event for obese persons. Selection of ADHD patients and control group not described detailed in present paper. From reference: ADHD patients recruited from out-patient clinics, controls self-selected, recruited from posters on public institutions and among college students and their acquaintances.	Total: 470; Obese group: 114; Control group: 154	**Obesity (E66):** BMI based on reported height and weight. **Circadian rhythm disturbance (G47;** see below) defined by manifestations of sleep problems and unstable eating patterns.	202	Not described in detail, they were included "afte extensive diagnostic assessment at the PsyQ outpatient clinic".
Bijlenga et al., 2013 (Bijlenga, van der Heijden, et al., 2013)	The Nether-lands	Cross sectional analysis; see above.	391	**Metabolic disorders in general (E70-E90):** Self-report questionnaires; see above.	202	Diagnosed by trained psychologist/ psychiatri outpatient clinic for adu ADHD; see above
Kutzbach et al., 2007 (Kutzbach, Summers, Holleschau	USA	Case study of children and adults diagnosed with albinism. Participants recruited at a conference of the National Organization for Albinism and	Total: 122 Adults: 44	**Albinism (E70.3):** Diagnosed by ophthalmologist using clinical criteria. In 78% also grouped by type of albinism.	Total: 20 Adults: 3 (6.8%)	Adults: self-report questionnaire. Adult AD diagnosed by positive response to the Utah cri for ADHD when suppor

, King, & MacDonald , 2007)		Hypopigmentation in Minneapolis and from the University of Minnesota International Albinism Center. Self-selection				by the history and the physician interview.
Muelly et al., 2013 (Muelly et al., 2013)	USA	Case control study. No information on how or from where participants were recruited, or on participation rate.	63. Maple syrup urine disease (MSUD) cases:37, of which 26 treated with diet, 11 liver transplan t. Controls: 26	**Maple syrup urine disease (E71.0):** No information on how or where cases were diagnosed	No clear informatio n on number of adult ADHD patients. Lifetime cumulative incidence: MUSD diet: 13 (54%) MUSD transplant: 9 (82%) Controls: 7 (27%)	No clear information. Conner's Parent Rating *DSM-IV* Sub scores are tabulated, but otherwise info on diagnosis of AD
Pearson et al., 2008 (Pearson et al., 2008)	USA	Matched case control study. Cases were RLS patients diagnosed at sleep- or medical centers, controls recruited through the media, from same local community as cases. Participation rates not given.	164: 110 RLS patients and 54 controls.	**Restless legs (G25):** Diagnosis based on clinical interview by neurologist.	Not given	ADHD diagnosis: Base medication use
Roy et al., 2015 (Roy et al., 2015)	Germany	Self-report questionnaires from a randomly selected community-based sample. Invited: 4069; agreed: 2520 (61.9%); final sample of adults: 1632 (40.1%)	1632	**Restless legs (G25):** Restless legs (RLS) based on validated self- report questionnaires. Overweight and sleep disturbance also assessed.	Adult ADHD:76, of which 9 also had RLS	A German ADHD self-rating scale
Schredl et al., 2007 (Schredl, Alm, & Sobanski, 2007)	Germany	Case-control study. Cases: ADHD patients from an out-patient clinic. Controls: No information on recruitment. Participation rate not given.	564	**Restless legs (G25):** Sleep disorders (see below). Restless legs based on the LISST sleep questionnaire, where "movement disorders" make up one group (5 items)	Total:120 Free of medication and without comorbidit y: 61	All cases met *DSM-IV* diagnoses of ADHD. BADDS*.
Steinlechne r et al., 2011 (Steinlechn er et al., 2011)	Germany	Interview and self- report questionnaires from 37 parents of 26 children diagnosed with ADHD. Recruited from pediatric department, participation rate not given.	37	**Restless legs (G25):** RLS (N=11) diagnosed by neurologist. Lifetime clinical psychiatric (Axis I) and personality (Axis II) disorders assessed by the Structured clinical interview for *DSM-IV* by psychiatrists.	ADHD: 6. With concurrent RLS: 5.	Structured clinical inter for *DSM-IV* by psychiat
Wagner et al., 2004	USA	Comparison between restless leg syndrome (RLS) patients, patients	171	**Restless legs (G25):** Patients were diagnosed with	Inattention and hyper-	*DSM-IV* criteria for AD The BADDS* question

(Wagner, Walters, & Fisher, 2004)		with insomnia and controls with respect to occurrence of ADHD. Patients with RLS or insomnia included sequentially from neurology clinic. Healthy controls (N=77) recruited through advertisement in hospital newsletter. Initially included 195 individuals, 171 (87.7%) remained after exclusions.		RLS by one of the authors using criteria developed by the International Restless Legs Syndrome Study Group (IRLSSG). The IRLSSG Rating Scale (self-report questionnaire) used for severity score.	activity (*DSM-IV* sympt score): RLS: N=16 (26%); Insomnia: N=2 (6%); Healthy control: N=4 (5%)	(self- report). For patien with scores indicating probable ADHD, also structured interview and neuropsychiatric test ba by neuropsychologist
Zak et al., 2009(Zak, Fisher, Couvadelli, Moss, & Walters, 2009)	USA	Pilot study on prevalence of Restless leg syndrome (RLS) in 30 adult ADHD patients. Recruitment from a psychological service specialized in treating adult ADHD, and from a sleep clinic (patients referred for RLS not included).	30	**Restless legs (G25):** Diagnosis by John Hopkins Telephone Diagnostic Interview for RLS	30, all inattentive type	Screened for ADHD according to *DSM-IV-T* criteria through intervie a neuropsychologist, following neuro-psychological testing to confirm diagnosis. Conners' Adult ADHD Rating Scale (self-repor symptoms severity.
Golimstok et al., 2011 (Golimstok et al., 2011)	Argen-tina	Matched case control study. Patients with Dementia with Lewy bodies (DLB), with Alzheimer and controls without neurological diseases, consecutively selected from record database at the Italian Hospital Medical Care Program in Buenos Aires. Case selection in 2000-2005.	509	**Dementia with Lewy bodies (G31.83):** Diagnosis by trained neurologist, consensus criteria used for DLB diagnosis, Dementia Rating Scale and Mini Mental Status Exam-ination for severity.	Preceding ADHD in DLB: 47.8%, in Alzheimer 15.2%, in controls: 15.1%	Two trained neurologist blind to the study objec diagnosed the participan using WURS* and appl *DSM-IV* criteria for aAI If discordant diagnosis, third (blind) neurologist assessed the patient. Information collected fr direct informant for pati with dementia.
Ettinger et al., 2015 (Ettinger et al., 2015)	USA	Community-based cross sectional survey, as part of the Epilepsy Comorbidity and Health Study (EPIC) study. 11-item screening survey sent to a random 340 000 individuals' population sample. Return rate 51%. Follow-up postal survey to 7500 persons with self-reported epilepsy, return rate 68%.	1361	**Epilepsy (G40):** Answering "yes" to: "Have you ever been told by a health care professional that you have epilepsy/seizure disorder", in addition to self-reported use of anitepileptic medicine. Additional scales assessing general health, mental health, quality of life.	251 screen positive on ASRS	Adult ADHD Self-Repo Scale (ASRS-6).
Ottman et al., 2011 (Ottman et al., 2011)	USA	Community-based cross sectional survey, as part of the Epilepsy Comorbidity and Health Study (EPIC) study. 11-item screening survey sent to a general population sample, returned from 172, 959 (51%).	172, 959	**Epilepsy (G40):** Answering "yes" to: "Have you ever been told by a health care professional that you have… [list of 16 disorders]?'' including epilepsy/seizure disorder.	13.2%	Answering ''yes'' to: "I you ever been told by a health professional that you have… [list of disorders]?'' including ADHD
van der Feltz-	The Nether-	Consecutive patients with new seizures, referred to a tertiary care	156, of which	**Epilepsy (G40):** Diagnosed at a tertiary epilepsy	6, of which 3 had	Standardized psychiatric interview to establish D

Cornelis (2006) (van der Feltz-Cornelis & Aldenkamp , 2006)	lands	epilepsy clinic, subjected to standardized psychiatric interview. Six ADHD patients included in an open treatment trial of methylphenidate.	126 had epilepsy	clinic, with EEG and video registration.	epilepsy and 3 Psycho-genic non-epileptic seizures	*IV* diagnostic classificat
Fasmer et al., 2011 (Fasmer, Halmoy, Oedegaard, & Haavik, 2011)	Norway	Cross sectional study comparing a case and a control group. ADHD cases collected from a national registry of adults with ADHD in 1997–2005, after 2005 also from psychiatrists/ psychologists nationwide. Participation rate not given.<br>Controls randomly selected from a nationwide population based registry.	1247	**Migraine (G43):**<br>Self-report questionnaire. Diagnosis based on answering yes to the question: "Have you ever had migraine?"	572	ADHD diagnoses from national registry of adul ADHD patients were al verified by 1 of 3 nation expert committees. Additional patients diagnosed by psychiatri psychologists. *ICD-10* research criteria used, b allowing the inattentive subtype as sufficient for diagnosis. Self-report questionnaires, includin ASRS*, WURS* and M
Fasmer et al., 2012 (Fasmer et al., 2012)	Norway	Cross sectional analysis of prescription data covering the entire country during one year	Total: 4,640,21 9	**Migraine (G43):**<br>Number and percent of Norwegian population being dispensed anti-migraine drugs at least once during 2006.	18,481	Number and percent of Norwegian population b dispensed ADHD drugs least once during 2006.
Ball et al., 1999 (Ball, Wooten, & Crowell, 1999)	USA	Review of literature and case report of six patients with Obstructive Sleep Apnea (OSA) recruited from a sleep disorder center.	6	**Sleep disorders (G47):**<br>OSA diagnosed with polysomnograms, severity by Respiratory Disturbance Index	6	Patients previously diagnosed with ADHD, diagnostic criteria not g WURS* (completed for assess whether ADHD symptoms present from childhood
Bijlenga et al., 2013 (Bijlenga, van der Heijden, et al., 2013)	The Nether-lands	Cross sectional analysis; see above.	391	**Sleep disorders (G47):**<br>Dutch questionnaires based on MEQ*,<br>Munich Chronotype Questionnaire<br>and the Seasonal Pattern Assessment Questionnaire–Global Seasonality Score.	202	Diagnosed by trained psychologist/ psychiatri outpatient clinic for adu ADHD;<br>see above
Bijlenga et al., 2013 (Bijlenga, Van Someren, et al., 2013)	The Nether-lands	Case control study.<br>Cases: adult ADHD patients with delayed sleep phase syndrome (DLPS), recruited from an outpatient adult ADHD clinic. Matched, healthy controls recruited by e-mail invitations.	24	**Sleep disorders (G47):**<br>DLPS based on self- report questionnaires on sleep hygiene, actigraphy, measurements of core body and skin temperature, melatonin measurements from saliva, sleep logs.	12	Diagnosed by trained psychologist/psychiatris outpatient clinic for adu ADHD, using DIVA 2.0 (semi-structured diagno interview based on *DSM* criteria).

		Participation rates not given.				
Boonstra et al., 2007 (Boonstra et al., 2007)	The Nether-lands	1) Case control comparison, Cases: adults with ADHD, self-referred or referred by other clinicians to outpatient clinic for assessment of ADHD. Matched controls without ADHD or other psychiatric disorders, no information on how they were recruited. Participation rates not given. 2) Double blind, placebo-controlled, cross-over medication trial among 31 of the 33 ADHD cases	1) 72 (33 ADHD patients, 39 controls 2) 31 ADHD patients	**Sleep disorders (G47):** 1) Comparison of circadian rhythm disturbances. Measured by actigraphy, sleep and activity logs. 2) Assessing effect of methylphenidate on circadian rhythm and sleep quality in ADHD patients	1) 33 2) 31	Diagnosed by trained psychiatrist at outpatien clinic for adult ADHD, using semistructured diagnostic interviews ar self-report questionnair *DSM-IV* criteria appliec
Fargason et al., 2013 (Fargason, Hollar, White, & Gamble, 2013)	USA	Case-control study. Cases: sample of all new referrals and established patients seen in an ADHD clinic over a 6-week period who met the inclusion criteria (ADHD but not active psychiatric symptoms, insomnia or circadian rhythm sleep disorder). Healthy, matched controls without ADHD and sleep disorders. No information on how controls were recruited, or participation rates.	105	**Sleep disorders (G47):** Sleep quality and rhythm. PSQI*. Separate questionnaire with questions on sleep timing and medication. Hamilton Depression Scale and Hamilton Anxiety Scale to exclude people with residual anxiety and depression.	80 ADHD + stimulants: 39; ADHD + non-stimulants: 15; ADHD + no medicine: 26;	Clinical interview, MIN and ASRS*. All establis clinical participants wer medication and all new referrals were unmedica awaiting psychopharmacological treatment.
Fisher et al., 2014 (Fisher et al., 2014)	Canada	Chart review over 20 years of patients with ADHD from one outpatient clinic, with respect to neuropsychological tests, sleep disorders and health symptoms	1828, of which 1163 adults	**Sleep disorders (G47):** Sleep, cognitive function, fatigue. Neuropsychological test battery with standard tests used over time. Self-report questionnaires: Personal Problems Checklist for Adults, Personal History Checklist for Adults, Patient Behavior Checklist for ADHD Adults, Physical Complaints Checklist for ADHD Adults	1163 adults; 877 inattentive type; 286 hyper-active/ impulsive and comorbid disorders	Clinical assessment by trained neuropsycholog using same neuropsychological test battery over 20 years, ir addition to self-report questionnaires with resp to sleep, attention, psychiatric and somatic health symptoms
Gamble et al., 2013 (Gamble, May, Besing, Tankersly, & Fargason, 2013)	USA	Analysis of a 2-week baseline phase of a randomized, placebo controlled crossover trial on effect of ramelteon on sleep problems in ADHD adults. Matched controls without ADHD, psychiatric disorders or sleep problems. Recruitment by newspaper ads and from outpatient psychiatry clinic. Participation rates not given.	38	**Sleep disorders (G47):** Insomnia or Delayed Sleep Phase Disorder assessed by clinical interview, meeting *DSM-IV-TR* criteria.	24; Combined type: 10 (42%); Inattentive type: 5 (21%); Hyper-active-impulsive type: 3 (13%); Symptoms	ADHD according to *DS IV-TR* criteria. Clinical interview by experience psychiatrist. ADHD Rat Scale, MINI*, Hamilto Depression and Hamilto Anxiety Scales.

					controlled: 6 (25%)	
Gau et al., 2007 (Gau et al., 2007)	Taiwan	Cross sectional survey using self-report questionnaires among college students. 60.8% response rate.	2284	**Sleep disorders (G47):** Various sleep problems (current and lifetime) by self-report questionnaires, based on the Sleep Habit Questionnaire. Definitions of sleep problems according to *DSM-IV* criteria.	Highly likely ADHD: 64 (2.8%) Probable ADHD: 74 (3.2%) Possible ADHD: 447 (19.6%)	Chinese version of ASR after 2-way translation.
Kass et al., 2003 (Kass, Wallace, & Vodanovich, 2003)	USA	Cross sectional survey using self-report questionnaires among a sample of students. No information on response rate.	148	**Sleep disorders (G47):** Epworth Sleepiness Scale, the Athens Insomnia Scale and The Boredom Proneness Scale.	12	Attention deficit scores the ABC* indicating AI
Kooij et al., 2001 (Kooij, Middelkoop, van Gils, & Buitelaar, 2001)	The Netherlands	Open-label case-control study. Adult ADHD patients from an outpatient clinic and healthy matched controls. No information on from where the controls were selected, or participation rate.	16	**Sleep disorders (G47):** Sleep log and actimeter six consecutive nights.	8	Semi-structured intervie using *DSM-IV* criteria. Presence of ADHD symptoms in childhood confirmed by family rep
Langberg et al., 2014 (Langberg, Dvorsky, Becker, & Molitor, 2014)	USA	Prospective, longitudinal study including undergraduate students with ADHD. Flyers, poster and e-mails offering a free diagnostic evaluation of students with previous ADHD diagnosis or difficulties with concentration and attention. Responders: 139, after telephone screening 94 eligible, 62 taking at least three courses at university.	62	**Sleep disorders (G47):** The Pediatric Daytime Sleepiness Scale. The Barkley Functional Impairment Scale. The Behaviour Assessment System for Children 2nd ed., Self-Report of Personality - College Version.	Total: 62 Inattentive: 35 Combined: 27 ADHD medicine: 36	*DSM-IV* criteria. BAAI IV*, CAADID* to both student and parent/guar
Mahajan ., 2010 (Mahajan, Hong, Wigal, & Gehricke, 2010)	USA	Case study including unmedicated ADHD patients without any major health problems, recruited from local colleges and clinical referrals. Participation rate not given.	22	**Sleep disorders (G47):** PSQI (self-report questionnaire).	22 Inattentive: 10 Combined: 9 Hyper-active/ impulsive subtype: 3	Semi structured intervie expert clinicians: QUES SCID*.
Middelkoop et al., 1997 (Middelkoop, Van Gils, &	The Netherlands	Case-control comparison of sleep characteristics between unmedicated ADHD patients and controls reporting to be physically and mentally in good health without sleep complaints.	22	**Sleep disorders (G47):** Actimetry and sleep logs during six consecutive nights. Subjective sleep quality assessed by using a five-point scale.	11	Diagnostic interview according to the *DSM-I* criteria including childh history of ADHD confi by family members.

Kooij, 1997)		No information on recruitment or participation rate.				
Naseem et al., 2001 (Naseem, Chaudhary, & Collop, 2001)	USA	Case report describing 3 ADHD cases referred to a sleep center.	3	**Sleep disorders (G47):** Sleep apnea diagnosed at a sleep clinic. Polysomnography, clinical examination.	3	Clinically diagnosed. No further information.
Oguzturk et al., 2013 (Oguzturk, Ekici, Cimen, Ekici, & Senturk, 2013)	Turkey	Clinically based survey of 113 patients referred to a hospital for assessment of sleep apnea after clinical referral. Cases: diagnosed with sleep apnea. Controls without sleep apnea. No further information on how controls were selected. Participation rates not given.	113	**Sleep disorders (G47):** Obstructive sleep apnea: nocturnal polysomnography and ESS.	81	ADHD scale based on [illegible] *IV* criteria.
Oosterloo et al., 2006 (Oosterloo, Lammers, Overeem, de Noord, & Kooij, 2006)	The Nether-lands	Self-report questionnaire based study. The ADHDRS* and the ESS* were sent to 140 patients previously diagnosed with primary hypersomnia at a narcolepsy clinic (returned with both questionnaires completed from 52.9%) and given to to 61 ADHD patients from outpatient clinic specialized in adult ADHD (completed by 100%).	135	**Sleep disorders (G47):** ESS*	61	ADHD patients: ASRS[illegible] SGIK* Diagnosed by experienc[illegible] clinicians according to *DSM-IV* criteria**.** Investigator-based ADH[illegible] Rating Scale.
Philipsen et al., 2005 (Philipsen et al., 2005)	Germany	Case control study including aADHD patients from an outpatient ADHD clinic and sex- and age matched healthy controls. Controls assessed by psychiatrists to rule out psychopathology, and healthy on the basis of physical examination and routine blood counting. Participation rate not given.	40	**Sleep disorders (G47):** Subjective: PSQI*, the Schlaffragebogen A. Objective: polysomnography: 2 nights in a sleep laboratory. First night adaption and exclusion of sleep apnea syndrome.	20	Fulfilling *DSM-IV* or *IC[illegible] 10* criteria. WURS*. Severity of symptoms i[illegible] adulthood self-rated on [illegible] point Likert scale corresponding to *DSM-*[illegible] Psychiatric comorbidity[illegible] assessed by structural clinical interview by experienced clinicians.
Sangal & Sangal, 2004 (Sangal & Sangal, 2004)	USA	Retrospective analysis of medical records in a neurophysiology practice investigating consecutive patients presenting with symptoms of sleep disorders or ADHD. Consecutive patients presenting with snoring and sleepiness and consecutive patients presenting with childhood inattention to evaluate the relationship between sleepiness and inattention.	56	**Sleep disorders (G47):** ESS* Sleepy snorers (n=38): Polysomnography and multiple sleep latency test.	18	ADHDRS* in patients [illegible] presented inattention in childhood. They met *DS[illegible] IV* criteria for ADHD o[illegible] ADHD in partial remiss[illegible] Number of partial remis[illegible] N/A.

Schredl et al., 2007 (Schredl et al., 2007)	Germany	Case- control study with ADHD cases from an out-patient clinic. See above	564	Sleep disorders (G47): Sleep questionnaires: The Schlaffragebogen A The Schlaffragebogen B LISST*	120 See above.	All cases met *DSM-IV* diagnoses of ADHD. Se above.
Sobanski et al., 2008(Sobanski, Schredl, Kettler, & Alm, 2008)	Germany	Matched case-control study including consecutive non-medicatied aADHD patients referred to adult ADHD clinic and healthy controls. Controls: participated in different sleep studies (referenced in article).	68	Sleep disorders (G47): The Schlaffragebogen A The Schlaffragebogen B. Polysomnography.. Psychiatric comorbidity:	34	Clinical interview: Consensus on diagnosis between senior psychiat and senior child-and adolescent psychiatrist. ADHD during childhoo and present according to *DSM-IV* criteria. WURS BADDS*. Psychiatric comorbidity semi-structured clinical interview, not named.
Surman et al., 2006 (Surman, Thomas, Aleardi, Pagano, & Biederman, 2006)	USA	Case study of ADHD patients consecutively referred to an adult ADHD program at a major academic center.	6	**Sleep disorders (G47):** MEQ*, PSQI*, ESS*, polysomnography.	6 Inattentive: 2 Combined: 4	*DSM-IV* criteria. SCID* modules from Kiddie-SADS*.
Surman et al., 2009 (Surman et al., 2009)	USA	Case-control study of adults with and without ADHD. Participants recruited via advertisements in the greater Boston area. ADHD subjects also from referrals to a psychiatric hospital clinic. Response rate not given.	299	Sleep disorders (G47): Self-report survey including "own made" questions about sleeping habits, and the Children´s sleep behaviour scale.	182	Lay interviewers: SCID modules from Kiddie-SADS*. Committee of clinicians: reviewed the from the interviews and agreed on diagnosis.
Van Veen et al., 2010 (Van Veen, Kooij, Boonstra, Gordijn, & Van Someren, 2010)	The Nether-lands	Matched case- control study. Cases with aADHD consecutively recruited from an out-patient clinic, grouped in those with and without sleep-onset insomnia. Controls: physically healthy with no history or symptoms of mental or sleeping disorders. No information on recruitment. Total number of controls not specified: 38 controls with data on dim light melatonin onset, 24 with actigraphy data. Participation rates not specified.	Not specified.	Sleep disorders (G47): SDQ* (Dutch). Actigraphy measured 7 consecutive days/nights. Salivary melatonin samples (one night).	40	Lifetime ADHD with childhood onset accordi the *DSM-IV* criteria, diagnosed by experienc clinicians. Semi structu interview for ADHD an comorbidity.
Vogel et al., 2015 (Vogel et al., 2015)	The Nether-lands	Case control study, see above	470, see above	Sleep disorders (G47): Circadian rhythm disturbance and obesity. Chronotype and sleep characteristics assessed with the "Vragenlijst Ochtend/Avondmens"	202	Not described in detail, they were included "afte extensive diagnostic assessment at the PsyQ outpatient clinic", see ab

				(Questionnaire Morning /Evening type) and the Munich Chronotype Questionnaire		
Voinescu et al., 2012 (Voinescu, Szentagotai , & David, 2012)	Romania	Study based on self-report questionnaires from two samples, one consisting of students (sample 1) and one from the general population from all over Romania (sample 2). Participation by self-selection. Individuals with likely ADHD, based on questionnaire scores, matched with controls with low scores.	551 Sample 1: 301 Sample 2: 250	**Sleep disorders (G47):** Self-report: The Sleep Condition Indicator, The Sleep Timing Questionnaire, SDQ*, CMQ*.	46	ASRS*, BAARS-IV*
Douniol et al., 2009 (Douniol et al., 2009)	France	Case study of individuals diagnosed with myotonic dystrophy type 1 at a specialized institute on muscle diseases and assessed for psychiatric comorbidity. 47 invited, participation rate 59.6%.	28	**Myotonic dystrophy (G71.1):** Confirmed by molecular diagnosis and onset between 1-10 years.	8, all inattentive	MINI*, ASRS*
Echenne et al., 2008 (Echenne et al., 2008)	France and Canada	Retrospective follow-up study with chart review of patients with myotonic dystrophy, including congenital and infantile/juvenile forms, followed by the same neurologists over 7-28 years (median 17 years).	32	**Myotonic dystrophy (G71.1):** Diagnosed by molecular biology analysis in the patients themselves or in their family	11 Myotonic dystrophy type 1: N=7 Post-natal myotonic dystrophy: N= 4	No information on how ADHD was diagnosed o the subjects were tested ADHD as adults.
Saez-Francas et al., 2012 (Saez-Francas et al., 2012)	Spain	Clinical sample of consecutive adults referred to an outpatient program at a university hospital due to symptoms of chronic fatigue. Original sample 169, after exclusions: 93.5%.	158	**Chronic Fatigue Syndrome (G93.3):** Diagnosed according to the Centers for Disease Control and Prevention criteria, a complete clinical assessment. The Fatigue Severity Scale and Fatigue Impact Scale.	33	WURS*, CAADID*, ADHD-RS*, BIS*.
Young, 2013 (Young, 2013)	USA	Case study. Three cases with chronic fatigue syndrome (CSF) responding poorly to treatment and referred for psychiatric consultation.	3	**Chronic Fatigue Syndrome (G93.3):** No information on how CSF was diagnosed. ESS* and the Fatigue Severity scale to measure sleep and the severity of fatigue.	3	Comprehensive psychia interview. BADDS*, A-ADDES*.
Kooij & Bijlenga, 2014 (Kooij & Bijlenga, 2014)	The Netherlands	Online survey with self-selected participants who reported photophobia. Participants invited through ADHD patient organizations, authors' Facebook and Twitter accounts, and therapists from outpatient Adult ADHD clinic.	494	**Photophobia (H53.14)** Online survey: Question on having photophobia apart from any migraine episodes ("My eyes are sensitive to light," yes or no), and more detailed questions if positive answer.	Total ADHD group: 231 ADHD diagnosis: 149 ADHD	Online survey: A multiple choice quest on having diagnosed AI (I have a diagnosis of ADHD; I do not have a diagnosis but I do have ADHD symptoms; I do have ADHD).

					symptoms: 82	
Olson et al., 2012 (Olson, Louwagie, Diehl, & Mohney, 2012)	USA	Retrospective case-control study, using review of medical records for cases with congenital esotropia (CES), and matched controls. Selection of cases through the resources of Rochester Epidemiology Project (REP), a medical records database where all medical records from all health care delivered in the region are linked.	254	**Congenital esotropia (H50.00):**<br>Recorded with diagnosis code in the REP.	14<br><br>CES cases: 8<br>Controls: 6	Medical records reviewe for diagnoses of mental illness as defined by *DS IV*, diagnosed by psychiatrist, family physician or emergency physician. Age at ADHI diagnosis not specified.
Bijlenga et al., 2013 (Bijlenga, van der Heijden, et al., 2013)	The Nether-lands	Cross sectional analysis; see above.	391	**Diseases of the Circulatory System (Chapter IX):**<br>Self- report questionnaires; see above.	202	Diagnosed by trained psychologist/ psychiatri outpatient clinic for adu ADHD; see above
Hodgkins et al., 2011 (Hodgkins et al., 2011)	USA	Retrospective analysis of health care claims and employer-rated health and productivity management database in 2006; see above.	156,973	**Diseases of the Circulatory System (Chapter IX):**<br>Information from databases, see above.	31,752	Diagnosis based on *ICD CM*. See above.
Semeijn et al., 2013 (Semeijn et al., 2013)	The Netherlands	Case control study; see above.	231	**Diseases of the Circulatory System (Chapter IX):**<br>Respondents asked about cardiac diseases and hypertension. Information from general practitioners. See above.	23	Screening list and DIVA see above
Spencer et al., 2014 (Spencer et al., 2014)	USA	Case control study; see above	198	**Diseases of the Circulatory System (Chapter IX):**<br>Self-reported history and measurements; see above.	98	Structured clinical inter for *DSM-IV*, and ASRS see above
Bijlenga et al., 2013 (Bijlenga, van der Heijden, et al., 2013)	The Nether-lands	Cross sectional analysis; see above.	391	**Allergic diseases in general (Chapter X):**<br>Self-report questionnaires; see above.	202	Diagnosed by trained psychologist/ psychiatri outpatient clinic for adu ADHD; see above
Chen et al., 2013 (Chen et al., 2013)	Taiwan	Registry based cross- sectional comparison of cases with ADHD, cases with TIC disorder, cases with both ADHD and TIC and a matched, randomly chosen control group using data from the Taiwan National Health Insurance Research Database (NHIRD).	Total 39,880<br>Cases: 7976<br>Controls: 31,904	**Allergic diseases in general (Chapter X):**<br>Asthma, allergic rhinitis, atopic dermatitis, allergic conjunctivitis, based on *ICD-9-CM* codes registered in Taiwan NHIRD. Diagnoses given at least twice.	ADHD alone: 5811<br>ADHD and Tic disorder: 349	Based on *ICD-9-CM* diagnosis codes registe NHIRD, Diagnoses giv least twice

Schmitt et al., 2016 (Schmitt, Stadler, Kuster, & Wustenberg, 2016)	Germany	Cohort study with German National Health Insurance beneficiaries registered in a population-based administrative healthcare database covering 55% of the population in Saxony; age and gender representative for the region and for Germany.	Total cohort: 1,811,094	**Allergic Rhinitis (J30):** Based on the *ICD-10* code registered in the healthcare database at least twice between 2005 and 2011	Numbers not given, only % and only for those <18 years	Based on the ICD-10 co (F90) registered at least twice in the healthcare database between 2005 2011
Bijlenga et al., 2013 (Bijlenga, van der Heijden, et al., 2013)	The Nether-lands	Cross sectional analysis; see above.	391	**Respiratory Disorders in general (Chapter X):** Self-report questionnaires; see above.	202	Diagnosed by trained psychologist/ psychiatri outpatient clinic for adu ADHD; see above
Semeijn et al., 2013 (Semeijn et al., 2013)	The Netherlands	Case control study; see above.	231	**Respiratory Disorders in general (Chapter X):** Respondents asked about having chronic nonspecific lung disease (asthma, chronic bronchitis, pulmonary emphysema) Information from general practitioners. See above.	23	Screening list and DIVA see above.
Fasmer et al., 2011 (Fasmer, Halmoy, Eagan, Oedegaard, & Haavik, 2011)	Norway	Cross sectional study based on self-report questionnaires comparing an ADHD case group with a control group	1313	**Asthma (J46):** Based on positive response to the question: Have you ever had asthma?	594	340 patients recruited fr national registry of adul ADHD patients, all diagnoses verified by 1 national expert committ Remaining 254 recruite through psychiatrists / psychologists nation-wi Diagnosis according to *10-R* criteria with modifications allowing inattentive subtype to be sufficient for diagnosis.
Chen et al., 2013 (Chen et al., 2013)	Taiwan	Registry based cross- sectional comparison between cases with ADHD, cases with TIC disorder, and with both and a matched, randomly chosen control group, see above	See above	**Asthma (J46):** Based on *ICD-9-CM* codes registered in Taiwan NHIRD. See above	See above.	Based on *ICD-9-CM* co registered in Taiwan NHIRD. See above
Fasmer et al., 2011 (Fasmer, Riise, et al., 2011)	Norway	Regsitry based cross sectional study using data from the Norwegian Prescription Database	Source popu-lation: 4,640,219	**Asthma (J46):** Defined as individuals being dispensed anti-asthma drugs at least once in 2006	18,481	Defined as individuals b dispensed ADHD drugs least once in 2006
Hodgkins et al., 2011 (Hodgkins et al., 2011)	USA	Retrospective analysis of health care claims and employer-rated health and productivity management database in 2006; see above.	156,973	**Asthma (J46):** Information from databases, see above.	31,752	Diagnosis based on *ICD CM*. See above.
Karlstad et	Norway	Registry based cross sectional	Standard	**Asthma (J46):**	ADHD	Individuals being dispen

al., 2012 (Karlstad, Nafstad, Tverdal, Skurtveit, & Furu, 2012)		study based on linked data from Norwegian census data, the Central Population Registry of Norway and the Norwegian Prescription Database	pop.: 1,239,533 Study pop.: (asthma cases): 37,060	Defined as individuals being dispensed reimbursed drugs for asthma, reimbursement diagnosis based on *ICD-10* or *ICPC-2* codes.	20-29 years: Males: 89 (1.6%) Females: 108 (1.6%)	reimbursed drugs for ADHD, with reimburse codes based on *ICD-10* *ICPC-2*.
Secnik et al., 2005 (Secnik et al., 2005)	USA	Registry based matched case control study; see above.	See above	**Asthma (J46):** Registered *ICD-9* diagnoses; see above	See above	Registered *ICD-9* code; above
Spencer et al., 2014 (Spencer et al., 2014)	USA	Case control study; see above.	198	**Asthma (J46):** Self-reported history of asthma; see above.	98	Structured clinical inter for *DSM-IV* and ASRS* above.
Bijlenga et al., 2013 (Bijlenga, van der Heijden, et al., 2013)	The Nether-lands	Cross sectional analysis; see above.	391	**Diseases of the Digestive System in general (Chapter K):** Self-report questionnaires; see above.	202	Diagnosed by trained psychologist/ psychiatri outpatient clinic for adu ADHD; see above
Hodgkins et al., 2011 (Hodgkins et al., 2011)	USA	Retrospective analysis of health care claims and employer-rated health and productivity management databases in 2006; see above.	156,973	**Irritabel bowel syndrome (K58):** Information from databases, see above.	31,752	Diagnosis based on *ICD* *CM*. See above.
Secnik et al., 200 (Secnik et al., 2005)	USA	Registry based matched case control study; see above.	See above	**Irritabel bowel syndrome (K58):** Registered *ICD-9* diagnosis; see above.	See above	Registered *ICD*-9 code; above.
Nieder-hofer & Pittschieler 2006 (Niederhofer & Pittschieler, 2006)	Italy	Case report of ADHD symptoms in 78 patients (60% of invited) with celiac disease before and after starting gluten-free diet.	78	**Celiac disease (K90.9):** Diagnosis based on positive blood serum levels (endomysium antibodies and other biomarkers) and histological examination of jejunal or duodenal mucosa.	Only given ADHD–like symptoms assessed by Conner Scale Hype-scheme	ADHD symptoms were assessed by Conner Sca Hypescheme (based on *DSM-IV* criteria) before 6 months after starting gluten-free diet.
Niederhofer, 2011 (Niederhofer, 2011)	Italy	Case report of 67 ADHD patients (87% of invited) where 10 were diagnosed with celiac disease. ADHD symptoms in these 10 were evaluated before and 6 months after starting gluten-free diet.	67	**Celiac disease (K90.9):** Blood serum levels of all included patients checked for endomysium antibodies and other biomarkers	67	All included patients ha diagnosis of ADHD, bu unknown from where or based on what criteria. ADHD symptoms were assessed by Conner Sca Hypescheme (based on *DSM-IV* criteria) before 6 months after starting gluten-free diet.

Zelnik et al., 2004 (Zelnik, Pacht, Obeid, & Lerner, 2004)	Israel	Matched case control study. Celiac disease (CD) cases recruited from pediatric gastroenterology clinic, non-CD control group recruited from same clinic. Participation rates not given.	322	**Celiac disease (K90.9):** Diagnosis based on postivie blood serum levels (endomysium antibodies and other biomarkers) and intestinal biopsies	Only given combined with learning disabilities : CD cases: Females: 13 (20.3%); Males: 10 (21.2%) Controls: Females: 11 (8.7%); Males: 11 (12.9%)	Initially based on self-re questionnaires (no detai given), followed by full neurological examinatio those who reported neurological symptoms. ADHD diagnosis and learning disabilities bas the diagnostic criteria o *DSM-IV*
Bijlenga et al., 2013 (Bijlenga, van der Heijden, et al., 2013)	The Nether-lands	Cross sectional analysis; see above.	391	**Skin disorders in general (Chapter XII):** Self-report questionnaires; see above.	202	Diagnosed by trained psychologist/ psychiatri outpatient clinic for adu ADHD; see above.
Cicek et al., 2009 (Cicek et al., 2009)	Turkey	Case control study from a dermatology clinic. Cases: atopic dermatitis (AD) patients. Controls: non-atopic patients from same clinic. Participation rates not given.	110	**Atopic dermatitis (L20):** Diagnosed in accordance with Hanifin Rajka classification.	Inattention criterion: 19 AD cases, 3 controls. Hyper-activity / impulsivity 20 AD cases, no controls Both criteria: 12 AD cases, no controls	Clinical interview using *DSM-IV* criteria. Self-report by Adult ADD/ADHD DSM-IV- Based Diagnostic Scree and Rating Scale.
Chu et al., 2012 (Chu et al., 2012)	Taiwan	Registry based matched case control study using data from the Taiwan National Health Insurance Research Database (NHIRD) from 2000 to 2009	25,585	**Alopecia areata (L63):** Alopecia areata (AA) based on registered *ICD-9* code in the NHIRD, and diagnosed by a dermatologist.	Total: 93; AA patients: 19 (0.4%); Controls: 74 (0.4%)	Based on registered *ICI* codes in the NHIRD, an diagnosed by a psychiat
Gupta et al., 2014 (Gupta, Gupta, & Vujcic, 2014)	Canada	Registry-based retrospective cross sectional study comparing acne patients with all other dermatology patients in national databases (National Ambulatory Medical Care Survey and	55,825	**Acne (ICD-10: L70).** Based on registered *ICD9-CM* codes.	110 (total group), the majority < 18 years	Based on registered *ICI* *CM* codes

		National Hospital Ambulatory Medical Care Survey)				
Bijlenga et al., 2013 (Bijlenga, van der Heijden, et al., 2013)	The Nether-lands	Cross sectional analysis; see above.	391	**Musculoskeletal disorders in general (Chapter XIII):** Self-report questionnaires; see above.	202	Diagnosed by trained psychologist/ psychiatri outpatient clinic for adu ADHD; see above
Spencer et al., 2014 (Spencer et al., 2014)	USA	Case control study; see above.	198	**Musculoskeletal disorders in general (Chapter XIII):** Self-reported history; see above	98	Structured clinical inter for *DSM-IV* and ASRS* above
Stray et al., 2013 (Stray et al., 2013)	Norway	Case control study. Cases: ADHD patients from outpatient clinic. Participation rate not given. Controls: self-selection through word-of-mouth and flyers on bulletin boards to students and health care workers.	48	**Musculoskeletal disorders in general (Chapter XIII):** Motor regulation problems assessed by the Motor Function Neurological Assessment battery (MFNU). Pain assessed by the Pain Drawing and the Numerical Pain Rating Scale	25	All were outpatients at a addiction unit and had b diagnosed with ADHD, information given on ho diagnosed. All were responders to methylphenidate, none l Substance use disorder.
Semeijn et al., 2013 (Semeijn et al., 2013)	The Netherla nds	Case control study; see above.	231	**Rheumatoid arthritis (M05-M06):** Respondents asked about having rheumatoid arthritis. Information from general practitioners. See above.	23	Screening list and DIVA see above.
Gao et al., 2015 (Gao, Lo, & Mok, 2015)	China	Matched case control study. Cases: consecutive adult patients at university-affiliated lupus clinic (participation rate not given). Participating cases were asked to invite one friend or peer of same age and sex who had good past health as healthy control.	181 117 SLE cases; 64 healthy con-trols	Systemic lupus erythematosus (M32): Patients recruited from lupus clinic. No current or recently active disease. Disease activity assessed by SLE Disease Activity Index, clinical manifestations and autoantibody profile from medical records.	SLE patients: Possible ADHD: 9 (7.7%) Controls: Possible ADHD: 4 (6.3%)	Possible ADHD based o self-report questionnaire Chinese version of the ASRS*; Part A (Inatten and Part B (Hyperactivi Impulsivity)
Garcia et al., 2013 (Garcia et al., 2013)	USA	1) Matched case control study. Cases: Systemic lupus erythematosus (SLE) patients, no info on how recruited or participation rate; Controls: "healthy subjects" recruited when donating blood for immune-biologic studies, no info on participation rate. 2) Placebo controlled trial with N-acetylcysteine to evaluate effect on ASRS* scores in SLE patients	1) Total: 95 SLE cases: 49; Con-trols: 46 2) Clinical trial: 24 SLE patients	**Systemic lupus erythematosus (M32):** Disease activity assessed by British Isles Lupus Assessment Group Index, and SLE Disease Activity Index. Fatigue assessed by the Fatigue Assessment Scale	N/A	Assessed by ASRS* sel report questionnaire. Sc compared.
Derksen et al., 2015 (Derksen,	The Netherla nds	Case report. 89 consecutive fibromyalgia patients from an outpatient	44	**Fibromyalgia (M79.7):** Diagnosis met the 1990 American College of	11	Interview by trained psychiatrist or assistant psychiatrist; not further

Vreeling, & Tchetverikov, 2015)		rheumatology clinic were invited. 50 patients included, 44 patients completed a psychiatry interview (49.4%)		Rheumatology criteria.		specified.
Krause et al., 1998 (Krause, Krause, Magyarosy, Ernst, & Pongratz, 1998)	Germany	Pilot study evaluating effect of ADHD-drug (moclobemide) on subjective and objective findings in fibromyalgia (FM) patients	24	**Fibromyalgia (M79.7):** No information given, patients had «proven fibromyalgia»	FM patients: 7 "probable ADHD" and 5 "highly probable". Controls: 2 "probable ADHD", none "highly probable"	Scores on Brown ADD* WURS* compared betw FM patients and control
Hailer et al., 2014 (Hailer, Haag, & Nilsson, 2014)	Sweden	Case study (patients with Legg-Calve-Perthes disease (LCPD)) comparing results with published data from the Swedish general population. Participation rate 80%	116	**Calvé-Legg-Perthes (M91.1):** Patients diagnosed or treated at Uppsala University Hospital between 1978-1995.	29 likely ADHD based on ASRS* scores, 4 were previously diagnosed with ADHD and used medication	ASRS* symptoms chec by interview
Berry et al., 2005(Berry, Leitner, Clarke, & Einfeld, 2005)	Australia	Review of clinical records for cases of genetically confirmed Angelman syndrome (AS) and presumed AS from clinical features, from an AS clinic. Compared with matched individuals with intellectual disability (ID) from an epidemiological register.	431	**Congenital syndromes and anomalies (Chapter XVII):** Angelman syndrome, gentically confirmed (N=62) and presumed based on clinifical features (N=29). Behavior tested by questionnaires including questions from the Developmental Behavior Checklist (DBC)	N/A	Behavior patterns from DBC were grouped in "ADHD type" and "Foo related" behaviors
Cornish et al., 2008 (Cornish et al., 2008)	United Kingdom	Matched case control study Cases recruited through the UK Clinical Genetics Service and the UK Fragile X Society No information on participation rate or control selection	107	**Congenital syndromes and anomalies (Chapter XVII):** Fragile X Syndrome premutation (carriers) (FXSp), genetically tested. IQ and neuropsychological tests. Neurology questionnaire on tremor symptoms and problems with gait and lower extremities	N/A	Increasing problems wit response inhibition with and decreased selective attention in all ages in F
Dorn et al., 1994	USA	Family informant study, where 24 daughters of Fragile X syndrome	56	**Congenital syndromes and anomalies (Chapter XVII):**	N/A	FXS carrier and control fathers were interviewed

(Dorn, Mazzocco, & Hagerman, 1994)		(FXS) carrier fathers and 32 daughters of control fathers were interviewed of their fathers' behaviors retrospectively. Recruitment from a regional developmental assessment clinic at hospital serving FXS families nationwide. Participation rate not given.		FXS carrier status.<br>24 fathers with FXS carrier status determined by pedigree analysis and DNA analysis. Outcome variables assessed by the Family Informant Schedule Criteria, an abuse questionnaire, the Parental Bonding Instrument and the Adult Attention-Deficit Hyperactivity (A-ADHD) checklist		the Adult Attention-Def Hyperactivity (A-ADHI checklist (*DSM-III-R* criteria).
Edvardson et al., 2014 (Edvardson et al., 2014)	Israel	Case study. Participants recruited from a Center for Down syndrome at a University Medical Center, Jerusalem. Participation rate 97.6%.	83	**Congenital syndromes and anomalies (Chapter XVII):** Down syndrome recruited from a Center for Down syndrome	Overall: 26 (31.3%) Mostly inattentive; 17 (65.4%) Mostly impulsive-hyper-activity; 4 (15.4%) Combined: 5 (19.2%)	Telephone interview of parents and guardians u the ADHD module of th Autism-Tics, Attention- Deficit/Hyperactivity Disorder, and other Comorbidities (A-TAC) Questionnaire
Gothelf et al., 2004 (Gothelf et al., 2004)	Israel	Case study.<br>51 consecutive patients with Velocardiofacial syndrome (VCFS), age 6-30 years, recruited from the clinical genetic departments of two major hospitals, 2001-2003. Participation rate not given	51	**Congenital syndromes and anomalies (Chapter XVII):** Velodcardiofacial syndrome, all genetically diagnosed, and all sporadic de novo cases.	Overall: 21 (41.2%) Mostly inattentive: 7 (33.3%) Combined type: 14 (66.7%)	Child psychiatrist blind the psychiatric status of individual, interviewed parents using Schedule Affective Disorders and Schizophrenia for Schoo Age children (K-SADS) screen positive on ADH the full-module section the K-SADS was used.
Halmoy et al., 2012 (Halmoy, Klungsoyr, Skjaerven, & Haavik, 2012)	Norway	Registry based nested case-control study. Cases were adult ADHD patients who were found eligible for stimulant treatment after a systematic diagnostic evaluation by one of three regional Expert committees on ADHD. Included cases were born from 1967 and registered in the Medical Birth Registry of Norway (MBRN). Controls were the remaining population, born in the same years, registered in the MBRN and surviving to adulthood.	1,172,396	**Congenital syndromes and anomalies (Chapter XVII):** Congenital oral clefts as registered in the MBRN at birth or the following stay at the Neonatal intensive care unit.	2323	Adult patients with suspected ADHD, were referred to one of three regional Expert Commi of ADHD for assessmen central stimulant treatm in a national trial period 1997-2005. Based on a systematic diagnostic evaluation, 70% were fo eligible for stimulant treatment (diagnosis confirmed and no contra indications).
Muzykewicz et al., 2007 (Muzykewicz,	USA	Retrospective chart review of 241 patients meeting clinical criteria for tuberous sclerosis (TSC). Charts were first screened, then reviewed by a psychiatrist, and 43	241	**Congenital syndromes and anomalies (Chapter XVII):** Details on diagnosis of **Tuberous sclerosis (TSC)** not given other than "meeting	73	From chart review: "73 (30%) patients had a his of ADHD type behavior 9 (21%) of the 43 patier seen by psychiatrist had

Newberry, Danforth, Halpern, & Thiele, 2007)		of 241 patients were referred to and seen by at least one of two psychiatrists affiliated with the TSC clinic.		clinical criteria for tuberous sclerosis (TSC) and seen by a single neurologist".		ADHD, 8 combined typ and 1 predominantly inattentive type.
Niklasson et al., 2009 (Niklasson, Rasmussen, Oskarsdottir, & Gillberg, 2009)	Sweden	Case study.<br>In-depth neuro-psychiatric assessment of 100 consecutive patients with 22q11.2 deletion syndrome (VCFS), all genetically confirmed, referred to a child neuro-psychiatric clinic.	100	**Congenital syndromes and anomalies (Chapter XVII):** 22q11.2 deletion syndrome, all genetically confirmed.	30	Neuropsychiatric evalua by experienced psychiat using extensive structur and semi-structured interviews, in accordanc with *DSM-IV* criteria. Neurocpsychological te battery and questionnair
Piran et al., 2011 (Piran et al., 2011)	Canada	Matched case control study, comparing three groups of Tetralogy of Fallot (TOF) patients: those with 22q11.2 deletion syndrome, those with syndromic features and those without syndromic features. Adult patients prospectively included from a cardiac center, for screening and chart review. Initially 447 patients with TOF, 207 (51%) included in study	207	**Congenital syndromes and anomalies (Chapter XVII):** Tetralogy of Fallot: three groups (syndromic, non-syndromic and with 22q11 deletion syndrome. Extracardiac anomalies. Endocrine disorders, other somatic disorders	Total: 8<br>6 in the syndromic group,<br>2 in the 22q11DS group,<br>none in the non-syndromic group	No information on how ADHD diagnosis was se but there was "extensive chart reviews"
Schneider et al., 2014 (Schneider et al., 2014)	Inter-national con-sortium	Collaborative case study of 22q11.2 deletion syndrome cases, age span 6-68 years. Cohorts from 15 sites in North America, Europe, Australia, Middle East. Participation rates from the various sites not given.	1402	**Congenital syndromes and anomalies (Chapter XVII):** 22q11.2 deletion syndrome, all genetically confirmed.	Overall 253.<br>Prevalence among adults = 15.6%<br>Inattentive type: 63 %<br>Hyper-active-impulsive type: 6.5%<br>Combined type: 30.5%	Assessments by well-validated instruments, fo adults, including SCID* Schedules for Clinical Assessment in Neuropsychiatry (SCAN MINI* and more. Psychiatric diagnosis in accordance with *DSM-I* criteria.
Tang et al., 2014 (Tang et al., 2014)	USA	Case study. Participants recruited from a children's hospital and from social networks. Participation rate not given.	112	**Congenital syndromes and anomalies (Chapter XVII):** 22q11.2 deletion syndrome, all genetically confirmed.	Overall:<br>35 (31%)<br>Adults (18-23 years):<br>6 (27%)<br>Adults (>23 years):<br>4 (21%)	Assessments by validate instruments, e.g. Structu Interview for Prodromal Syndromes (SIPS), and SCID*. Interviews of probands and informant experienced interviewer Narratives of each case discussed on case conference attended by doctoral-level clinicians
Bijlenga et	The	Cross sectional analysis; see	391	**Symptoms/signs involving the**	202	Diagnosed by trained

al., 2013 (Bijlenga, van der Heijden, et al., 2013)	Netherlands	above.		**urinary system, in general:** Self-report questionnaires; see above.		psychologist/ psychiatri outpatient clinic for adu ADHD; see above
Caci et al., 2014 (Caci et al., 2014)	France	Questionnaires from parents of youths participating in a large study on ADHD symptoms in the community; see above..	1171	**Enuresis (R32):** Based on answering yes to a question about having enuresis.	ADHD defined by 2-phase method: 2.99%	ASRS* filled in and sco in three ways; see above
Hodgkins et al., 2011 (Hodgkins et al., 2011)	USA	Retrospective analysis of health care claims and employer-rated health and productivity management database in 2006; see above.	156,973	**Enuresis (R32):** Information from databases, see above.	31,752	Diagnosis based on *ICD CM*. See above.
Secnik et al., 200 (Secnik et al., 2005)	USA	Registry based matched case control study; see above.	See above	**Enuresis (R32):** Registered *ICD-9* diagnosis; see above.	See above.	Registered *ICD-9* code; above.
Bijlenga et al., 2013 (Bijlenga, van der Heijden, et al., 2013)	The Netherlands	Cross sectional analysis; see above.	391	**Cancer, unspecified:** Self-report questionnaires; see above.	202	Diagnosed by trained psychologist/ psychiatri outpatient clinic for adu ADHD; see above
Semeijn et al., 2013 (Semeijn et al., 2013)	The Nether-lands	Case control study, see above.	231	**Cancer, unspecified:** Respondents asked about having cancer. Information from general practitioners. See above.	23	Screening list and DIVA see above.

References:

Alfonsson, S., Parling, T., & Ghaderi, A. (2012). Screening of adult ADHD among patients presenting for bariatric surgery. *Obesity Surgery, 22*(6), 918-926.

Altfas, J. R. (2002). Prevalence of attention deficit/hyperactivity disorder among adults in obesity treatment. *BMC Psychiatry, 2*, 9.

Anderson, S. E., Cohen, P., Naumova, E. N., & Must, A. (2006). Relationship of childhood behavior disorders to weight gain from childhood into adulthood. *Ambulatory pediatrics: the official journal of the Ambulatory Pediatric Association, 6*(5), 297-301.

Supplementary 3.

Ball, J. D., Wooten, V., & Crowell, T. (1999). Adult ADHD and/or Sleep Apnea? Differential Diagnostic Considerations with Six Case Studies. *Journal of Clinical Psychology in Medical Settings*, *6*(3), 259-271.

Berry, R. J., Leitner, R. P., Clarke, A. R., & Einfeld, S. L. (2005). Behavioral aspects of Angelman syndrome: A case control study. *American Journal of Medical Genetics, 132 A*(1), 8-12.

Biederman, J., Spencer, T. J., Monuteaux, M. C., & Faraone, S. V. (2010). A naturalistic 10-year prospective study of height and weight in children with attention-deficit hyperactivity disorder grown up: sex and treatment effects. *Journal of Pediatrics, 157*(4), 635-640, 640.e631.

Bijlenga, D., van der Heijden, K. B., Breuk, M., van Someren, E. J., Lie, M. E., Boonstra, A. M., . . . Kooij, J. J. (2013). Associations between sleep characteristics, seasonal depressive symptoms, lifestyle, and ADHD symptoms in adults. *Journal of Attention Disorders, 17*(3), 261-275.

Bijlenga, D., Van Someren, E. J., Gruber, R., Bron, T. I., Kruithof, I. F., Spanbroek, E. C., & Kooij, J. J. (2013). Body temperature, activity and melatonin profiles in adults with attention-deficit/hyperactivity disorder and delayed sleep: a case-control study. *Journal of sleep research, 22*(6), 607-616.

Boonstra, A. M., Kooij, J. J., Oosterlaan, J., Sergeant, J. A., Buitelaar, J. K., & Van Someren, E. J. (2007). Hyperactive night and day? Actigraphy studies in adult ADHD: a baseline comparison and the effect of methylphenidate. *Sleep, 30*(4), 433-442.

Brook, J. S., Brook, D. W., Zhang, C., Seltzer, N., & Finch, S. J. (2013). Adolescent ADHD and adult physical and mental health, work performance, and financial stress. *Pediatrics, 131*(1), 5-13.

Brucker-Davis, F., Skarulis, M. C., Grace, M. B., Benichou, J., Hauser, P., Wiggs, E., & Weintraub, B. D. (1995). Genetic and clinical features of 42 kindreds with resistance to thyroid hormone. The National Institutes of Health Prospective Study. *Annals of Internal Medicine*, *123*(8), 572-583.

Caci, H. M., Morin, A. J., & Tran, A. (2014). Prevalence and correlates of attention deficit hyperactivity disorder in adults from a French community sample. *The Journal of nervous and mental disease, 202*(4), 324-332.

Chen, M. H., Su, T. P., Chen, Y. S., Hsu, J. W., Huang, K. L., Chang, W. H., & Bai, Y. M. (2013). Attention deficit hyperactivity disorder, tic disorder, and allergy: is there a link? A nationwide population-based study. *Journal of Child Psychology & Psychiatry & Allied Disciplines*, *54*(5), 545-551.

Chu, S. Y., Chen, Y. J., Tseng, W. C., Lin, M. W., Chen, T. J., Hwang, C. Y., . . . Liu, H. N. (2012). Psychiatric comorbidities in patients with alopecia areata in Taiwan: A case-control study. *British Journal of Dermatology, 166*(3), 525-531.

Cicek, D., Kandi, B., Dertlioglu, S. B., Gunay, S., Halisdemir, N., Turgay, A., & Colak, C. (2009). Investigation of attention deficit and hyperactivity disorder in adult patients with atopic dermatitis. *International journal of psychiatry in clinical practice, 13*(4), 292-297.

Cornish, K. M., Li, L., Kogan, C. S., Jacquemont, S., Turk, J., Dalton, A., . . . Hagerman, P. J. (2008). Age-dependent cognitive changes in carriers of the fragile X syndrome. *Cortex, 44*(6), 628-636.

Cortese, S., Faraone, S. V., Bernardi, S., Wang, S., & Blanco, C. (2013). Adult attention-deficit hyperactivity disorder and obesity: epidemiological study. *British Journal of Psychiatry, 203*(1), 24-34.

Cortese, S., Moreira-Maia, C. R., St Fleur, D., Morcillo-Peñalver, C., Rohde, L. A., & Faraone, S. V. (2015). Association Between ADHD and Obesity: A Systematic Review and Meta-Analysis. American Journal of Psychiatry,173(1), 34-43.

Cortese, S., Ramos Olazagasti, M. A., Klein, R. G., Castellanos, F. X., Proal, E., & Mannuzza, S. (2013). Obesity in men with childhood ADHD: a 33-year controlled, prospective, follow-up study. *Pediatrics, 131*(6), e1731-1738.

Davis, C., Patte, K., Levitan, R. D., Carter, J., Kaplan, A. S., Zai, C., . . . Kennedy, J. L. (2009). A psycho-genetic study of associations between the symptoms of binge eating disorder and those of attention deficit (hyperactivity) disorder. *Journal of Psychiatric Research, 43*(7), 687-696.

de Zwaan, M., Gruss, B., Muller, A., Philipsen, A., Graap, H., Martin, A., . . . Hilbert, A. (2011). Association between obesity and adult attention-deficit/hyperactivity disorder in a German community-based sample. *Obesity Facts, 4*(3), 204-211.

Derksen, M. T., Vreeling, M. J., & Tchetverikov, I. (2015). High frequency of adult attention deficit hyperactivity disorder among fibromyalgia patients in the Netherlands: should a

systematic collaboration between rheumatologists and psychiatrists be sought? *Clinical and experimental rheumatology, 33*(1 Suppl 88), S141.

Docet, M. F., Larranaga, A., Fernandez Sastre, J. L., & García-Mayor, R. V. (2010). High rate of attention deficit hyperactivity disorder in obese adults: a case-control study. *Obesity and Metabolism 6*, 121-124.

Docet, M. F., Larranaga, A., Perez Mendez, L. F., & Garcia-Mayor, R. V. (2012). Attention deficit hyperactivity disorder increases the risk of having abnormal eating behaviours in obese adults. *Eating & Weight Disorders: EWD, 17*(2), e132-136.

Dorn, M. B., Mazzocco, M. M., & Hagerman, R. J. (1994). Behavioral and psychiatric disorders in adult male carriers of fragile X. *Journal of the American Academy of Child & Adolescent Psychiatry, 33*(2), 256-264.

Douniol, M., Jacquette, A., Guile, J. M., Tanguy, M. L., Angeard, N., Heron, D., . . . Cohen, D. (2009). Psychiatric and cognitive phenotype in children and adolescents with myotonic dystrophy. *European Child & Adolescent Psychiatry, 18*(12), 705-715.

Echenne, B., Rideau, A., Roubertie, A., Sébire, G., Rivier, F., & Lemieux, B. (2008). Myotonic dystrophy type I in childhood: Long-term evolution in patients surviving the neonatal period. *European Journal of Paediatric Neurology, 12*(3), 210-223.

Edvardson, S., Msallam, N., Hertz, P., Malkiel, S., Wexler, I. D., & Tenenbaum, A. (2014). Attention deficit hyperactivity disorders symptomatology among individuals with Down syndrome. *Journal of Policy and Practice in Intellectual Disabilities, 11*(1), 58-61.

Ettinger, A. B., Ottman, R., Lipton, R. B., Cramer, J. A., Fanning, K. M., & Reed, M. L. (2015). Attention-deficit/hyperactivity disorder symptoms in adults with self-reported epilepsy: Results from a national epidemiologic survey of epilepsy. *Epilepsia, 56*(2), 218-224.

Fargason, R. E., Hollar, A. F., White, S., & Gamble, K. L. (2013). Adults with ADHD-without insomnia history have subclinical sleep disturbance but not circadian delay: an ADHD phenotype? *Journal ofAttention Disorders, 17*(7), 583-588.

Fasmer, O. B., Halmoy, A., Eagan, T. M., Oedegaard, K. J., & Haavik, J. (2011). Adult attention deficit hyperactivity disorder is associated with asthma. *BMC Psychiatry, 11*, 128. doi:10.1186/1471-244X-11-128

Fasmer, O. B., Halmoy, A., Oedegaard, K. J., & Haavik, J. (2011). Adult attention deficit hyperactivity disorder is associated with migraine headaches. *European Archives of Psychiatry & Clinical Neuroscience, 261*(8), 595-602.

Fasmer, O. B., Riise, T., Eagan, T. M., Lund, A., Dilsaver, S. C., Hundal, O., & Oedegaard, K. J. (2011). Comorbidity of asthma with ADHD. *Journal of Attention Disorders, 15*(7), 564-571.

Fasmer, O. B., Riise, T., Lund, A., Dilsaver, S. C., Hundal, O., & Oedegaard, K. J. (2012). Comorbidity of migraine with ADHD. *Journal of Attention Disorders,, 16*(4), 339-345.

Fisher, B. C., Garges, D. M., Yoon, S. Y., Maguire, K., Zipay, D., Gambino, M., & Shapiro, C. M. (2014). Sex differences and the interaction of age and sleep issues in neuropsychological testing performance across the lifespan in an ADD/ADHD sample from the years 1989 to 2009. *Psychological Reports, 114*(2), 404-438.

Fleming, J. P., Levy, L. D., & Levitan, R. D. (2005). Symptoms of attention deficit hyperactivity disorder in severely obese women. *Eating & Weight Disorders: EWD, 10*(1), e10-13.

Gamble, K. L., May, R. S., Besing, R. C., Tankersly, A. P., & Fargason, R. E. (2013). Delayed sleep timing and symptoms in adults with attention-deficit/hyperactivity disorder: a controlled actigraphy study. *Chronobiology international, 30*(4), 598-606.

Gao, Y., Lo, Y., & Mok, M. Y. (2015). Symptoms of attention deficit hyperactivity disorder in patients with systemic lupus erythematosus. Lupus, 24(14), 1498-1504. Garcia, R. J., Francis, L., Dawood, M., Lai, Z. W., Faraone, S. V., & Perl, A. (2013). Attention deficit and hyperactivity disorder scores are elevated and respond to N-acetylcysteine treatment in patients with systemic lupus erythematosus. *Arthritis and rheumatism, 65*(5), 1313-1318.

Garcia, R. J., Francis, L., Dawood, M., Lai, Z. W., Faraone, S. V., & Perl, A. (2013). Attention deficit and hyperactivity disorder scores are elevated and respond to N-acetylcysteine treatment in patients with systemic lupus erythematosus. *Arthritis Rheumatology, 65*(5), 1313-1318.

Gau, S. S., Kessler, R. C., Tseng, W. L., Wu, Y. Y., Chiu, Y. N., Yeh, C. B., & Hwu, H. G. (2007). Association between sleep problems and symptoms of attention-deficit/hyperactivity disorder in young adults. *Sleep, 30*(2), 195-201.

Golimstok, A., Rojas, J. I., Romano, M., Zurru, M. C., Doctorovich, D., & Cristiano, E. (2011). Previous adult attention-deficit and hyperactivity disorder symptoms and risk of dementia with Lewy bodies: a case-control study. *European Journal of Neurology, 18*(1), 78-84.

Gothelf, D., Presburger, G., Levy, D., Nahmani, A., Burg, M., Berant, M., . . . Weizman, A. (2004). Genetic, developmental, and physical factors associated with attention deficit hyperactivity disorder in patients with velocardiofacial syndrome. *American Journal of Medical Genetics Part B: Neuropsychiatric Genetics, 126B*(1), 116-121.

Gupta, M. A., Gupta, A. K., & Vujcic, B. (2014). Increased frequency of Attention Deficit Hyperactivity Disorder (ADHD) in acne versus dermatologic controls: analysis of an epidemiologic database from the US. *Journal of Dermatological Treatment, 25*(2), 115-118.

Hailer, Y. D., Haag, A. C., & Nilsson, O. (2014). Legg-Calve-Perthes disease: Quality of life, physical activity, and behavior pattern. *Journal of Pediatric Orthopaedics, 34*(5), 514-521.

Halmoy, A., Klungsoyr, K., Skjaerven, R., & Haavik, J. (2012). Pre- and perinatal risk factors in adults with attention-deficit/hyperactivity disorder. *Biological Psychiatry, 71*(5), 474-481.

Hodgkins, P., Montejano, L., Sasane, R., & Huse, D. (2011). Cost of illness and comorbidities in adults diagnosed with attention-deficit/hyperactivity disorder: A retrospective analysis. *Primary Care Companion to the Journal of Clinical Psychiatry, 13*(2).

Hosain, G., Berenson, A. B., Tennen, H., Bauer, L. O., & Wu, Z. (2012). Attention deficit hyperactivity symptoms and risky sexual behavior in young adult women. *Journal of Women's Health, 21*(4), 463-468.

Karlstad, O., Nafstad, P., Tverdal, A., Skurtveit, S., & Furu, K. (2012). Comorbidities in an asthma population 8-29 years old: a study from the Norwegian Prescription Database. *Pharmacoepidemiology and drug safety, 21*(10), 1045-1052.

Kass, S. J., Wallace, J. C., & Vodanovich, S. J. (2003). Boredom proneness and sleep disorders as predictors of adult attention deficit scores. *Journal of Attention Disorders, 7*(2), 83-91.

Kooij, J. J., & Bijlenga, D. (2014). High prevalence of self-reported photophobia in adult ADHD. *Frontiers in neurology, 5*, 256.

Kooij, J. J., Middelkoop, H. A., van Gils, K., & Buitelaar, J. K. (2001). The effect of stimulants on nocturnal motor activity and sleep quality in adults with ADHD: an open-label case-control study. *The Journal of clinical psychiatry, 62*(12), 952-956.

Krause, K.-H., Krause, J., Magyarosy, I., Ernst, E., & Pongratz, D. (1998). Fibromyalgia syndrome and attention deficit hyperactivity disorder: Is there a comorbidity and are there consequences for the therapy of fibromyalgia syndrome? *Journal of Musculoskeletal Pain, 6*(4), 111-116.

Kutzbach, B., Summers, C. G., Holleschau, A. M., King, R. A., & MacDonald, J. T. (2007). The prevalence of attention-deficit/hyperactivity disorder among persons with albinism. *Journal of Child Neurology, 22*(12), 1342-1347.

Langberg, J. M., Dvorsky, M. R., Becker, S. P., & Molitor, S. J. (2014). The impact of daytime sleepiness on the school performance of college students with attention deficit hyperactivity disorder (ADHD): a prospective longitudinal study. *Journal of sleep research, 23*(3), 318-325.

Levy, L. D., Fleming, J. P., & Klar, D. (2009). Treatment of refractory obesity in severely obese adults following management of newly diagnosed attention deficit hyperactivity disorder. *International Journal of Obesity, 33*(3), 326-334.

Mahajan, N., Hong, N., Wigal, T. L., & Gehricke, J. G. (2010). Hyperactive-impulsive symptoms associated with self-reported sleep quality in nonmedicated adults with ADHD. *Journal of Attention Disorders, 14*(2), 132-137.

Middelkoop, H., Van Gils, K., & Kooij, J. (1997). Adult attention-deficit hyperactivity disorder (ADHD): Actimetric evaluation of nocturnal motor activity and subjective sleep characteristics. *Sleep-Wake Research in The Netherlands, 8*, 87-90.

Muelly, E. R., Moore, G. J., Bunce, S. C., Mack, J., Bigler, D. C., Morton, D. H., & Strauss, K. A. (2013). Biochemical correlates of neuropsychiatric illness in maple syrup urine disease. *Journal of Clinical Investigation, 123*(4), 1809-1820.

Muzykewicz, D. A., Newberry, P., Danforth, N., Halpern, E. F., & Thiele, E. A. (2007). Psychiatric comorbid conditions in a clinic population of 241 patients with tuberous sclerosis complex. *Epilepsy & Behavior, 11*(4), 506-513.

Naseem, S., Chaudhary, B., & Collop, N. (2001). Attention deficit hyperactivity disorder in adults and obstructive sleep apnea. *Chest, 119*(1), 294-296.

Nazar, B. P., Suwwan, R., de Sousa Pinna, C. M., Duchesne, M., Freitas, S. R., Sergeant, J., & Mattos, P. (2014). Influence of attention-deficit/hyperactivity disorder on binge eating behaviors and psychiatric comorbidity profile of obese women. *Comprehensive Psychiatry, 55*(3), 572-578.

Niederhofer, H. (2011). Association of attention-deficit/hyperactivity disorder and celiac disease: a brief report. *The primary care companion to CNS disorders, 13*(3).

Niederhofer, H., & Pittschieler, K. (2006). A preliminary investigation of ADHD symptoms in persons with celiac disease. *Journal of Attention Disorders, 10*(2), 200-204.

Nigg, J. T., Johnstone, J. M., Musser, E. D., Long, H. G., Willoughby, M. T., & Shannon, J. (2016). Attention-deficit/hyperactivity disorder (ADHD) and being overweight/obesity: New data and meta-analysis. *Clinical Psychology Review, 43*, 67-79.

Niklasson, L., Rasmussen, P., Oskarsdottir, S., & Gillberg, C. (2009). Autism, ADHD, mental retardation and behavior problems in 100 individuals with 22q11 deletion syndrome. *Research in Developmental Disabilities*, 30(4), 763-773.

Oguzturk, O., Ekici, M., Cimen, D., Ekici, A., & Senturk, E. (2013). Attention deficit/hyperactivity disorder in adults with sleep apnea. *Journal of Clinical Psychology in Medical Settings, 20*(2), 234-239.

Olson, J. H., Louwagie, C. R., Diehl, N. N., & Mohney, B. G. (2012). Congenital esotropia and the risk of mental illness by early adulthood. *Ophthalmology, 119*(1), 145-149.

Oosterloo, M., Lammers, G. J., Overeem, S., de Noord, I., & Kooij, J. J. (2006). Possible confusion between primary hypersomnia and adult attention-deficit/hyperactivity disorder. *Psychiatry Research, 143*(2-3), 293-297.

Ottman, R., Lipton, R. B., Ettinger, A. B., Cramer, J. A., Reed, M. L., Morrison, A., & Wan, G. J. (2011). Comorbidities of epilepsy: results from the Epilepsy Comorbidities and Health (EPIC) survey. *Epilepsia, 52*(2), 308-315.

Pagoto, S. L., Curtin, C., Bandini, L. G., Anderson, S. E., Schneider, K. L., Bodenlos, J. S., & Ma, Y. (2010). Weight loss following a clinic-based weight loss program among adults with attention deficit/hyperactivity disorder symptoms. *Eating & Weight Disorders: EWD, 15*(3), e166-172.

Pagoto, S. L., Curtin, C., Lemon, S. C., Bandini, L. G., Schneider, K. L., Bodenlos, J. S., & Ma, Y. (2009). Association between adult attention deficit/hyperactivity disorder and obesity in the US population. *Obesity, 17*(3), 539-544.

Pearson, V. E., Gamaldo, C. E., Allen, R. P., Lesage, S., Hening, W. A., & Earley, C. J. (2008). Medication use in patients with restless legs syndrome compared with a control population. *European Journal of Neurology, 15*(1), 16-21.

Philipsen, A., Feige, B., Hesslinger, B., Ebert, D., Carl, C., Hornyak, M., . . . Riemann, D. (2005). Sleep in adults with attention-deficit/hyperactivity disorder: a controlled polysomnographic study including spectral analysis of the sleep EEG. *Sleep, 28*(7), 877-884.

Piran, S., Bassett, A. S., Grewal, J., Swaby, J. A., Morel, C., Oechslin, E. N., . . . Silversides, C. K. (2011). Patterns of cardiac and extracardiac anomalies in adults with tetralogy of Fallot. *American Heart Journal, 161*(1), 131-137.

Roy, M., de Zwaan, M., Tuin, I., Philipsen, A., Brahler, E., & Muller, A. (2015). Association Between Restless Legs Syndrome and Adult ADHD in a German Community-Based Sample. Journal of Attention Disorders. Advance online publication. doi:10.1177/1087054714561291

Saez-Francas, N., Alegre, J., Calvo, N., Antonio Ramos-Quiroga, J., Ruiz, E., Hernandez-Vara, J., & Casas, M. (2012). Attention-deficit hyperactivity disorder in chronic fatigue syndrome patients. *Psychiatry Research, 200*(2-3), 748-753.

Sangal, R. B., & Sangal, J. M. (2004). Rating scales for inattention and sleepiness are correlated in adults with symptoms of sleep disordered breathing syndrome, but not in adults with symptoms of attention-deficit/hyperactivity disorder. *Sleep Medicine, 5*(2), 133-135.

Schmitt, J., Stadler, E., Kuster, D., & Wustenberg, E. G. (2016). Medical care and treatment of allergic rhinitis. A population-based cohort study based on routine healthcare utilization data. *Allergy, 71*(6), 850-858.

Schneider, M., Debbane, M., Bassett, A. S., Chow, E. W., Fung, W. L., van den Bree, M., . . . Behavior in 22q11.2 Deletion, S. (2014). Psychiatric disorders from childhood to adulthood in 22q11.2 deletion syndrome: results from the International Consortium on Brain and Behavior in 22q11.2 Deletion Syndrome. *American Journal of Psychiatry, 171*(6), 627-639.

Schredl, M., Alm, B., & Sobanski, E. (2007). Sleep quality in adult patients with attention deficit hyperactivity disorder (ADHD). *Eur Arch Psychiatry Clin Neurosci, 257*(3), 164-168.

Secnik, K., Swensen, A., & Lage, M. J. (2005). Comorbidities and costs of adult patients diagnosed with attention-deficit hyperactivity disorder. *PharmacoEconomics, 23*(1), 93-102.

Semeijn, E. J., Kooij, J. J., Comijs, H. C., Michielsen, M., Deeg, D. J., & Beekman, A. T. (2013). Attention-deficit/hyperactivity disorder, physical health, and lifestyle in older adults. *Journal of the American Geriatrics Society, 61*(6), 882-887.

Sobanski, E., Schredl, M., Kettler, N., & Alm, B. (2008). Sleep in adults with attention deficit hyperactivity disorder (ADHD) before and during treatment with methylphenidate: a controlled polysomnographic study. *Sleep, 31*(3), 375-381.

Spencer, T. J., Faraone, S. V., Tarko, L., McDermott, K., & Biederman, J. (2014). Attention-deficit/hyperactivity disorder and adverse health outcomes in adults. *The Journal of nervous and mental disease, 202*(10), 725-731.

Steinlechner, S., Bruggemann, N., Sobottka, V., Benthien, A., Behn, B., Klein, C., . . . Lencer, R. (2011). Restless legs syndrome as a possible predictor for psychiatric disorders in parents of children with ADHD. *European archives of psychiatry and clinical neuroscience, 261*(4), 285-291.

Stray, L. L., Kristensen, O., Lomeland, M., Skorstad, M., Stray, T., & Tonnessen, F. E. (2013). Motor regulation problems and pain in adults diagnosed with ADHD. *Behavioral and Brain Functions, 9*, 18.

Strimas, R., Davis, C., Patte, K., Curtis, C., Reid, C., & McCool, C. (2008). Symptoms of attention-deficit/hyperactivity disorder, overeating, and body mass index in men. *Eating behaviors,* 9(4), 516-518.

Surman, C. B., Adamson, J. J., Petty, C., Biederman, J., Kenealy, D. C., Levine, M., . . . Faraone, S. V. (2009). Association between attention-deficit/hyperactivity disorder and sleep impairment in adulthood: evidence from a large controlled study. *Journal of Clinical Psychiatry, 70*(11), 1523-1529.

Surman, C. B., Thomas, R. J., Aleardi, M., Pagano, C., & Biederman, J. (2006). Adults with ADHD and sleep complaints: a pilot study identifying sleep-disordered breathing using polysomnography and sleep quality assessment. *Journal of Attention Disorders, 9*(3), 550-555.

Tang, S., Yi, J., Calkins, M., Whinna, D., Kohler, C., Souders, M., . . . Gur, R. (2014). Psychiatric disorders in 22q11.2 deletion syndrome are prevalent but undertreated. *Psychological Medicine, 44*(6), 1267-1277.

van der Feltz-Cornelis, C. M., & Aldenkamp, A. P. (2006). Effectiveness and safety of methylphenidate in adult attention deficit hyperactivity disorder in patients with epilepsy: an open treatment trial. *Epilepsy & Behavior, 8*(3), 659-662.

Van Veen, M. M., Kooij, J. J., Boonstra, A. M., Gordijn, M. C., & Van Someren, E. J. (2010). Delayed circadian rhythm in adults with attention-deficit/hyperactivity disorder and chronic sleep-onset insomnia. *Biological Psychiatry, 67*(11), 1091-1096.

Vogel, S. W., Bijlenga, D., Tanke, M., Bron, T. I., van der Heijden, K. B., Swaab, H., . . . Kooij, J. J. (2015). Circadian rhythm disruption as a link between Attention-Deficit/Hyperactivity Disorder and obesity? *Journal of Psychosomatic Research, 79*(5), 443-450.

Voinescu, B. I., Szentagotai, A., & David, D. (2012). Sleep disturbance, circadian preference and symptoms of adult attention deficit hyperactivity disorder (ADHD). *Journal of Neural Transmission, 119*(10), 1195-1204.

Wagner, M. L., Walters, A. S., & Fisher, B. C. (2004). Symptoms of attention-deficit/hyperactivity disorder in adults with restless legs syndrome. *Sleep, 27*(8), 1499-1504.

Young, J. L. (2013). Chronic fatigue syndrome: 3 cases and a discussion of the natural history of attention-deficit/hyperactivity disorder. *Postgraduate Medicine, 125*(1), 162-168.

Zak, R., Fisher, B., Couvadelli, B. V., Moss, N. M., & Walters, A. S. (2009). Preliminary study of the prevalence of restless legs syndrome in adults with attention deficit hyperactivity disorder. *Percept Mot Skills, 108*(3), 759-763.

Zelnik, N., Pacht, A., Obeid, R., & Lerner, A. (2004). Range of neurologic disorders in patients with celiac disease. *Pediatrics, 113*(6), 1672-1676.

SUPPLEMENTARY 3

Different diagnostic tools used to evaluate ADHD, other psychiatric disorders, and tools used to assess specific somatic disorder described in several articles in this review. Due to the large number of comorbid somatic disorders, only the most frequently used evaluating tools are described. We refer to each specific article for more information.

Type	Name	Abbreviation	Use
**ADHD**			
Self-report questionnaire			
	Adult ADHD clinical diagnostic scale	ACDS	Clinician-based, semi-structured interview consisting of 18 items investigating current adult symptoms of ADHD. Version 1.2 includes a retrospective assessment of all symptoms of childhood ADHD and assessment of recent (past 6 months) symptoms of adult ADHD (aADHD) covering both *DSM-IV* symptoms and 14 non-*DSM* symptoms believed to be relevant to aADHD such as mood lability (Adler & Cohen, 2004).
	ADHD self-rating behaviour	ADHD-SR	German self-rating behavior questionnaire covering aADHD symptoms according to *DSM-IV* and *ICD-10* research criteria

questionnaire		(Rosler et al., 2004).
Adult Behavior Checklist	ABC	An 18-item checklist divided in two subscales, Attention and Hyperactivity, assessing ADHD symptoms according to *DSM-IV* criteria based on self-report data (Kass, Wallace, & Vodanovich, 2003).
ADHD Rating Scale	ADHDRS–IV	Check list for parents and teachers covering ADHD symptoms to closely approximate the *DSM-IV* diagnostic criteria in children and adolescence from 4-20 years. The purpose is to provide clinicians information on ADHD (DuPaul, 1998).
Adult ADHD Self-Report Scale / Adult ADHD Self-Report Scale Screener	ASRS<br>ASRS-S	Developed in conjunction with the World Health Organization and is designed to measure current ADHD symptoms. Consists of 18 items covering the *DSM-IV-TR* criteria for ADHD and the core symptoms of ADHD: inattention, impulsivity and hyperactivity. A high symptom score on ASRS is not sufficient to clinically diagnose ADHD in adults, but is frequently used both clinically and in research to define study populations with possible ADHD (Kessler et al., 2005).
The Assessment of Adult Attention Deficit Hyperactivity Disorder		In longitudinal studies, a questionnaire designed to follow-up ADHD symptoms in adults diagnosed with childhood ADHD (Mannuzza et al., 2011)
Barkley Adult ADHD Rating Scale-IV	BAARS-IV	Based on the *DSM* diagnostic ADHD criteria, it covers both childhood and adult ADHD symptoms (Barkley, 2011)
Barrat Impulsivity Scale	BIS	Self-report measure designed to evaluate impulsivity at the time of assessment (Patton, Stanford, & Barratt, 1995).
Brown Attention	BADDS	Covers a wide range of symptoms focusing on inattention (Thomas

	Deficit Disorder Scale		E Brown, 1996). Hyperactivity and impulsivity are not sufficiently addressed (Kooij et al., 2008).
	Conners' Adult ADHD Rating Scale	CAARS	Covers inattention, hyperactivity, impulsivity, as well as emotional lability (Conners, Erhardt, & Sparrow, 1999).
	Wender Utah Rating Scale	WURS	Retrospectively assesses symptoms of ADHD in childhood (Ward, Wender, & Reimherr, 1993).
Interviews			
	Adult Attention Deficit Disorders Evaluation Scale	A-ADDES	Provides clinicians information on aADHD symptoms. It is available in three versions, one self-report, one reporting from close relation/friend, and one from co-workers (McCarney S., 1996).
	Hyperactivity disorder (ADHD) Screening List		A short questionnaire developed to distinguish adults with ADHD from community controls and people with clinical disorders other than ADHD. It has shown good validity when used in older individuals > 60 years (Semeijn et al., 2013).
	The Diagnostic Interview for ADHD in adults	DIVA	A semi-structured interviewed shown to be reliable in diagnosing ADHD in adults (Ramos-Quiroga et al., 2016).
	Structured interview Conners' adult ADHD diagnostic interview for the DSM-IV	CAADID	Assesses current and childhood symptoms, impairment and pervasiveness of symptoms over time (Conners, Epstein, & Johnson, 2001).
	The QUEST method	QUEST	A semi structured clinical interview assessing adult ADHD symptoms according to *DSM-IV*, providing age-appropriate probes. Queries about current problems, symptoms and comorbidities are

			included (Wigal et al., 2007).
	The Schedule for Affective Disorders and Schizophrenia for School-Age Children	Kiddie-SADS	A semi structured diagnostic interview used to assess current and lifetime psychiatric history, and can be adapted to be used in adults. One module assesses ADHD symptoms (Kaufman et al., 1997).
		SGIK	Dutch semi structured diagnostic interview assessing current and childhood ADHD symptoms (Bekker et al., 2005)
**Psychiatric comorbidity**			
	Beck Depression Inventory	BDI	Measuring severity of depressive symptoms, consisting of 21 questions assessing depressive symptoms the last two weeks. It is not intended to serve as a sole diagnostic instrument for depression. (Beck & Beamesderfer, 1974).
Self-report questionnaire	The Hospital Anxiety and Depression scale.	HAD	The Hospital Anxiety- Depression Scale (HAD) is designed to recognize symptoms of anxiety and depression in patients with physical illness. It also measures the severity of emotional disorder (Zigmond & Snaith, 1983).
	Hamilton Anxiety Rating Scale	HAM-A	Rating scale used by clinicians to rate the severity of anxiety symptoms (Hamilton, 1959)
	Hamilton Depression Rating Scale	HAM-D	Rating scale used by clinicians to rate the severity of depression symptoms (Hamilton, 1980)
	Mood Disorder Questionnaire	MDQ	Short screening questionnaire for bipolar spectrum disorders validated for use in the general population and in psychiatric patient populations (Hirschfeld et al., 2003; Hirschfeld et al., 2000).
	The Symptom	(SCL-90-R)	A multidimensional inventory assessing psychiatric symptoms and

	Checklist-90 (-R)		psychological distress the preceding seven days. It can be used in both clinical and community samples and gives a severity index of general mental distress as well as assessing nine psychiatric symptoms dimensions (Derogatis, 1996).
Interview.			
	The World Health Organization World Mental Health Composite International Diagnostic Interview	CIDI	A comprehensive, fully-structured standardized interview designed to be used by trained lay interviewers for the assessment of mental disorders consistent with *DSM-IV* and *ICD-10* (Robins et al., 1988).
	Diagnostic Interview Schedule for DSM-IV	DIS-IV	A fully-structured interview designed to diagnose major psychiatric disorders according to *DSM-IV* that can be used by non-clinican interviewers (Segal, 2010).
	The Mini-International Neuropsychiatric Interview	M.I.N.I	A short structured diagnostic interview developed to investigate major psychiatric disorders as described in *DSM-IV* (Axis 1) and *ICD-10*. It was designed to capture routine and repetitive information to be used in clinical trials and epidemiology studies, and as a first step in a clinical evaluation of a patient (Sheehan et al., 1998).
	Mini International Neuropsychiatric Interview Plus	M.I.N.I. Plus	Similar to M.I.N.I., but with a more extensive interview, also including a module for ADHD (Sheehan et al., 1998).
	Psychiatric Research Interview for Substance and Mental	PRISM	A diagnostic interview to assess affective disorders, anxiety disorders, psychotic symptoms, eating disorders and personality disorders in individuals who drink heavily or use drugs. (Hasin et

	Disorders		al., 1996)
	The Structured Clinical Interview for the Diagnostic and Statistical Manual of Mental Disorders-IV (DSM-IV) Axis I disorders	SCID I	A diagnostic semi-structured interview assessing major *DSM-IV* Axis I (clinical) diagnoses (First, Spitzer, Gibbon, & Williams, 1997)
**Somatic comorbidity**			
Nutritional disorder, Obesity			
Can be both self-reported and objectively measured.	Body mass index	BMI	Used to identify overweight and obesity, and is defined as weight in kilograms divided by height in meters squared. In adults, < 18.5 kg/m^2 is defined as underweight, 18.5 to <25 kg/m^2 defined as normal, 25.0 to <30 kg/m^2 is defined as overweight and a BMI of ≥30 kg/m^2 is defined as obese (World Health Organization, 1992). BMI is a simple and easy way to evaluate obesity and is useful to evaluate obesity trends in the general population. However, BMI does not provide an accurate measurement of body fat on the individual level, nor does it take sex, age and ethnicity into account (Bhurosy & Jeewon, 2013).
Self-reported questionnaire			
	The Dutch Eating	DEBQ	Self-reported questionnaire measuring emotional, external and

	Behaviour Questionnaire		restrained eating, eating styles likely to be associated with the development of overweight (van Strien, Frijters, Bergers, & Defares, 1986).
**Sleep disorders**			
Self-report questionnaire			
	The Composite Scale of Morningness	CMQ	Determines circardian typology (morning activities, morning affect, and eveningness) (Smith, Reilly, & Midkiff, 1989). It is developed from a combination of some items from the MEQ (Horne & Ostberg, 1976) and a diurnal scale by Torsvall and Akerstedt (Torsvall & Akerstedt, 1980).
	Epworth Sleepiness Scale	ESS	Measures daytime sleepiness and can be used to differentiate between different sleep disorders, such as central hypersomnias and sleep-disordered breathing from insomnia (Johns, 1991)
	Horne-Ostberg Morningness and Eveningness Scale/Morningness–Eveningness Questionnaire	MEQ	Suited to measure circadian sleep-phase, and is an indicator of natural sleep cycle (Horne & Ostberg, 1976)
	The Landecker Inventar sur Erfassung von Schlafstörungen	LISST	A screening instrument to detect different sleep disorders, like insomnia, nocturnal breathing disorders, restless legs, parasomnias and sleep/wake rhythm disorders (Weeß, Schürmann, & Steinberg, 2002).
	Pittsburgh sleep	PSQI	Subjectively measures sleep quality and disturbances over a 1-

	quality index		month time. It is a screening tool identifying patients that may require further sleep testing and is accurate in distinguishing good versus bad sleep patterns (Buysse, Reynolds, Monk, Berman, & Kupfer, 1989). It is not designed to define the presence of insomnia, but has been useful to differentiate people with and without insomnia (Backhaus, Junghanns, Broocks, Riemann, & Hohagen, 2002).
	The Schlaffragebogen A		German sleep questionnaire measuring sleep quality the preceding night (Görtelmeyer, 1985, 2011).
	The Schlaffragebogen B		German sleep questionnaire measuring sleep quality and the feeling of being refreshed in the morning the previous 2 weeks (Görtelmeyer, 2011).
	The Sleep Disorders Questionnaire	SDQ	Self-report questionnaire evaluating the presence of insomnia according to the *DSM-IV* and International Classification of Sleep Disorders-Revised (Violani, Devoto, Lucidi, Lombardo, & Russo, 2004).
	The Dutch Sleep Disorder Questionnaire	SDQ (Dutch)	(SDQ) is a questionnaire used to evaluate symptoms of common sleep disorders including insomnia, sleep apnea and restless legs syndrome (Sweere et al., 1998).
	Sleep log/sleep diary		A simple and convenient way to self- report sleep patterns at a daily basis, and is used to diagnose sleep disorders such as insomnia, delayed sleep phase syndrome and narcolepsy (Ramar & Olson, 2013). The information provided can also be used to assess the effect of the treatment of sleep disorder.
Objective measurement			

Supplementary 3.

Actigraphy	Used to assess sleep patterns and circadian rhytms. Actigraphy is a non-invasive objective method performed by an actigraph, traditionally records motor activity and sleep parameters. It as an electronic device worn on the body, often like a small watch-like device. Later year actigraphs have developed and can include features such as light- and temperature measurement and pulse recording. The sleep patterns are derived from nightly activity scores (De Crescenzo et al., 2016). Compared to PSG, actigraphy can assess sleep in a natural environment and can easily record sleep patterns over week's duration. Compared to sleep logs, it is more reliable as it does not depend on the patient´s recall. On the other hand, polysomnography collects more comprehensive information from different data sources (Ancoli-Israel et al., 2003).
Polysomnography (PSG).	Used to record several physiologic parameters relevant to sleep, such as electroencephalography (EEG), electrooculography (EOG), electrocardiography (ECG), chin- and anterio tibilais electromyography (EMG), respiratory effort, airflow and oximetry (Chesson et al., 1997). Polysomnography is used assessing a number of different sleep related disorders, such as restless legs syndrome, periodic limb movements during sleep, central hypersomnias, circadian rhythm sleep disorder and sleep-disordered breathing (Kushida et al., 2005).

References:

Adler, L., & Cohen, J. (2004). Diagnosis and evaluation of adults with attention-deficit/hyperactivity disorder. *Psychiatric Clinics of North America, 27*(2), 187-201.

Backhaus, J., Junghanns, K., Broocks, A., Riemann, D., & Hohagen, F. (2002). Test-retest reliability and validity of the Pittsburgh Sleep Quality Index in primary insomnia. *Journal of Psychosomatic Research, 53*(3), 737-740.

Barkley, R. A. (2011). *Barkley Adult ADHD Rating Scale-IV (BAARS-IV).* New York: Guilford Press.

Beck, A. T., & Beamesderfer, A. (1974). Assessment of depression: the depression inventory. *Modern Problems of Pharmacopsychiatry, 7*(0), 151-169.

Bekker, E. M., Overtoom, C. E., Kooij, J., Buitelaar, J. K., Verbaten, M. N., & Kenemans, J. (2005). Disentangling deficits in adults with attention-deficit/hyperactivity disorder. *Archives of General Psychiatry, 62*(10), 1129-1136.

Bhurosy, T., & Jeewon, R. (2013). Pitfalls of using body mass index (BMI) in assessment of obesity risk. *Current Research in Nutrition and Food Science Journal, 1*(1), 71-76.

Buysse, D. J., Reynolds, C. F., 3rd, Monk, T. H., Berman, S. R., & Kupfer, D. J. (1989). The Pittsburgh Sleep Quality Index: a new instrument for psychiatric practice and research. *Psychiatry Research, 28*(2), 193-213.

Chesson, A. L., Jr., Ferber, R. A., Fry, J. M., Grigg-Damberger, M., Hartse, K. M., Hurwitz, T. D., . . . Sher, A. (1997). The indications for polysomnography and related procedures. *Sleep, 20*(6), 423-487.

Conners, C. K., Epstein, J., & Johnson, D. E. (2001). *Conner's Adult ADHD Diagonostic Interview for DSM-IV: CAADID: Technical Manual.* Toronto: Multi-Health Systems Inc.

Conners, C. K., Erhardt, D., & Sparrow, E. P. (1999). *Conners' adult ADHD rating scales (CAARS): technical manual*. Toronto: Multi-Health Systems Inc.

Derogatis, L. R. (1996). *SCL-90-R: Symptom Checklist-90-R: administration, scoring, and procedures manual*. Bloomington, MN: NCS Pearson.

DuPaul, G., Power TJ, Anastopoulos AD, Reid R. (1998). *ADHD Rating Scale-IV: Checklists, Norms, and Clinical Interpretation.* New York, NY: Guilford.

First, M. B., Spitzer, R. L., Gibbon, M., & Williams, J. B. (1997). *User's guide for the Structured clinical interview for DSM-IV axis I disorders SCID-I: clinician version*. Arlington, IA: American Psychiatric Pub.

Görtelmeyer, R. (1985). On the development of a standardized sleep inventory for the assessment of sleep. *Methods of sleep research*, 93-98.

Görtelmeyer, R. (2011). *Schlaffragebogen* A und B-Revidierte Fassung Assessment of Sleep [Standardized Sleep Inventory for the SF-A/R and SF-B/R—revised version]. Göttingen: Hogrefe.

Hamilton, M. (1959). The assessment of anxiety states by rating. *British Journal of Medical Psychology, 32*(1), 50-55.

Hamilton, M. (1980). Rating depressive patients. *Journal of Clinical Psychiatry, 41*(12, Sec 2), 21-24.

Hasin, D. S., Trautman, K. D., Miele, G. M., Samet, S., Smith, M., & Endicott, J. (1996). Psychiatric Research Interview for Substance and Mental Disorders (PRISM): reliability for substance abusers. *American Journal of Psychiatry, 153*(9), 1195-1201.

Hirschfeld, R. M., Holzer, C., Calabrese, J. R., Weissman, M., Reed, M., Davies, M., . . . Hazard, E. (2003). Validity of the mood disorder questionnaire: a general population study. *American Journal of Psychiatry, 160*(1), 178-180.

Hirschfeld, R. M., Williams, J. B., Spitzer, R. L., Calabrese, J. R., Flynn, L., Keck, P. E., Jr., . . . Zajecka, J. (2000). Development and validation of a screening instrument for bipolar spectrum disorder: the Mood Disorder Questionnaire. *American Journal of Psychiatry, 157*(11), 1873-1875.

Horne, J. A., & Ostberg, O. (1976). A self-assessment questionnaire to determine morningness-eveningness in human circadian rhythms. *International journal of chronobiology, 4*(2), 97-110.

Johns, M. W. (1991). A new method for measuring daytime sleepiness: the Epworth sleepiness scale. *Sleep, 14*(6), 540-545.

Kass, S. J., Wallace, J. C., & Vodanovich, S. J. (2003). Boredom proneness and sleep disorders as predictors of adult attention deficit scores. *Journal of Attention Disorders, 7*(2), 83-91.

Kaufman, J., Birmaher, B., Brent, D., Rao, U. M. A., Flynn, C., Moreci, P., . . . Ryan, N. (1997). Schedule for Affective Disorders and Schizophrenia for School-Age Children-Present and Lifetime Version (K-SADS-PL): Initial Reliability and Validity Data. *Journal of the American Academy of Child & Adolescent Psychiatry, 36*(7), 980-988.

Kessler, R. C., Adler, L., Ames, M., Demler, O., Faraone, S., Hiripi, E., . . . Walters, E. E. (2005). The World Health Organization Adult ADHD Self-Report Scale (ASRS): a short screening scale for use in the general population. *Psychol Med, 35*(2), 245-256.

Kooij, J. J., Boonstra, A. M., Swinkels, S. H., Bekker, E. M., de Noord, I., & Buitelaar, J. K. (2008). Reliability, validity, and utility of instruments for self-report and informant report concerning symptoms of ADHD in adult patients. *Journal of Attention Disorders, 11*(4), 445-458.

Kushida, C. A., Littner, M. R., Morgenthaler, T., Alessi, C. A., Bailey, D., Coleman, J., Jr., . . . Wise, M. (2005). Practice parameters for the indications for polysomnography and related procedures: an update for 2005. *Sleep, 28*(4), 499-521.

Mannuzza, S., Castellanos, F. X., Roizen, E. R., Hutchison, J. A., Lashua, E. C., & Klein, R. G. (2011). Impact of the impairment criterion in the diagnosis of adult ADHD: 33-year follow-up study of boys with ADHD. *Journal of Attention Disorders, 15*(2), 122-129.

McCarney S., A. P. D., Jackson M. T. . (1996). *Adult Attention Deficit Disorders Evaluation Scale: Self-report version technical manual.*: Columbia, MO: Hawthorne Educational Services.

Patton, J. H., Stanford, M. S., & Barratt, E. S. (1995). Factor structure of the barratt impulsiveness scale. *Journal of Clinical Psychology, 51*(6), 768-774.

Ramar, K., & Olson, E. J. (2013). Management of common sleep disorders. *American family physician, 88*(4), 231-238.

Robins, L. N., Wing, J., Wittchen, H. U., Helzer, J. E., Babor, T. F., Burke, J., . . . et al. (1988). The Composite International Diagnostic Interview. An epidemiologic Instrument suitable for use in conjunction with different diagnostic systems and in different cultures. *Archives of General Psychiatry*, *45*(12), 1069-1077.

Rosler, M., Retz, W., Retz-Junginger, P., Thome, J., Supprian, T., Nissen, T., . . . Trott, G. E. (2004). [Tools for the diagnosis of attention-deficit/hyperactivity disorder in adults. Self-rating behaviour questionnaire and diagnostic checklist]. *Nervenarzt*, *75*(9), 888-895.

Segal, D. L. (2010). *Diagnostic Interview Schedule for DSM-IV (DIS-IV). The Corsini Encyclopedia of Psychology*: John Wiley & Sons, Inc.

Semeijn, E. J., Michielsen, M., Comijs, H. C., Deeg, D. J. H., Beekman, A. T. F., & Kooij, J. J. S. (2013). Criterion Validity of an Attention Deficit Hyperactivity Disorder (ADHD) Screening List for Screening ADHD in Older Adults Aged 60–94 years. *The American Journal of Geriatric Psychiatry, 21*(7), 631-635.

Sheehan, D. V., Lecrubier, Y., Sheehan, K. H., Amorim, P., Janavs, J., Weiller, E., . . . Dunbar, G. C. (1998). The Mini-International Neuropsychiatric Interview (M.I.N.I.): the development and validation of a structured diagnostic psychiatric interview for DSM-IV and ICD-10. *Journal of Clinical Psychiatry, 59 Suppl 20*, 22-33;quiz 34-57.

Smith, C. S., Reilly, C., & Midkiff, K. (1989). Evaluation of three circadian rhythm questionnaires with suggestions for an improved measure of morningness. *Journal of Applied Psychology*, *74*(5), 728-738.

Sweere, Y., Kerkhof, G. A., De Weerd, A. W., Kamphuisen, H. A., Kemp, B., & Schimsheimer, R. J. (1998). The validity of the Dutch Sleep Disorders Questionnaire (SDQ). *Journal of Psychosomatic Research, 45*(6), 549-555.

Torsvall, L., & Akerstedt, T. (1980). A diurnal type scale. Construction, consistency and validation in shift work. *Scandinavian Journal of Work, Environment & Health, 6*(4), 283-290.

van Strien, T., Frijters, J. E. R., Bergers, G. P. A., & Defares, P. B. (1986). The Dutch Eating Behavior Questionnaire (DEBQ) for assessment of restrained, emotional, and external eating behavior. *International Journal of Eating Disorders, 5*(2), 295-315.

Violani, C., Devoto, A., Lucidi, F., Lombardo, C., & Russo, P. M. (2004). Validity of a Short Insomnia Questionnaire: the SDQ. *Brain Research Bulletin, 63*(5), 415-421.

Ward, M. F., Wender, P. H., & Reimherr, F. W. (1993). The Wender Utah Rating Scale: an aid in the retrospective diagnosis of childhood attention deficit hyperactivity disorder. *American Journal of Psychiatry, 150*(6), 885-890.

Weeß, H., Schürmann, T., & Steinberg, R. (2002). Das Landecker Inventar zur Erfassung von Schlafstörungen (LISST). *Kompendium Schlafmedizin. Landsberg :Ecomed.*

Wigal, T., Wigal, S., Steinhoff, K., Kollins, S., Newcorn, J., Steinberg-Epstein, R., . . . Swanson, J. (2007). Establishing a clinical diagnosis of ADHD in adults: the QUEST method. *Advances in ADHD, 2*, 17-24.

World Health Organization. (1992). *The ICD-10 Classification of Mental and Behavioural Disorders: Clinical Descriptions and Diagnostic Guidelines*: Royal College of Psychiatrists.

Zigmond, A. S., & Snaith, R. P. (1983). The Hospital Anxiety and Depression Scale. *Acta Psychiatrica Scandinavica, 67*(6), 361-370.
